# Need or opportunity? A study of innovations in equids

**DOI:** 10.1371/journal.pone.0257730

**Published:** 2021-09-27

**Authors:** Konstanze Krueger, Laureen Esch, Richard Byrne

**Affiliations:** 1 Department Equine Economics, Faculty Agriculture, Economics and Management, Nuertingen-Geislingen University, Nürtingen, Germany; 2 Zoology/Evolutionary Biology, University of Regensburg, Regensburg, Germany; 3 Department of Veterinary Sciences, Faculty of Veterinary Medicine, Animal Hygiene and Animal Husbandry, Chair of Animal Welfare, Ethology, Ludwig Maximilian University Munich, Munich, Germany; 4 Centre for Social Learning & Cognitive Evolution, School of Psychology & Neuroscience, University of St Andrews, St Andrews, Scotland, United Kingdom; Institute of Animal Science, CZECH REPUBLIC

## Abstract

Debate persists over whether animals develop innovative solutions primarily in response to needs or conversely whether they innovate more when basic needs are covered and opportunity to develop novel behaviour is offered. We sourced 746 cases of “unusual” behaviour in equids by contacting equid owners and caretakers directly and via a website (https://innovative-behaviour.org), and by searching the internet platforms YouTube and Facebook for videos. The study investigated whether differences in need or opportunity for innovation were reflected in the numbers of different types of innovations and in the frequencies of repeating a once-innovative behaviour (i) with respect to the equids’ sex, age, and breed type, (ii) across behavioural categories, and whether (iii) they were affected by the equids’ management (single vs group housing, access to roughage feed, access to pasture, and social contact). We found that the numbers of different types of innovation and the frequency of displaying specific innovations were not affected by individual characteristics (sex, age, breed or equid species). Few types of innovation in escape and foraging contexts were observed, whilst the comfort, play, and social contexts elicited the greatest variety of innovations. We also found higher numbers of different types of innovations in horses kept in groups rather than in individual housing, and with unlimited rather than with restricted access to pasture and roughage. Equids in permanent social contact performed high rates of once-innovative behaviour. We suggest that equids produce goal-directed innovations and repeat the behaviour at high frequency in response to urgent needs for food and free movement or when kept in conditions with social conflict. However, equids devise the greatest variety of innovations when opportunity to play and to develop comfort behaviour arises and when kept in good conditions.

## Introduction

Innovation is defined as producing a novel solution to environmental or social challenges [1] and animals that find solutions to a novel problem or a novel solution to an old problem can be considered innovative [2]. Debate persists about the animals’ motivation to innovate [3, 4].

One hypothesis is that *need* is the driving force behind animal innovation [1, 5, 6]. From this perspective, individuals with poor access to resources, potentially caused by high competition for resources, by poor competitive abilities or simply by resource shortage, are forced to develop novel solutions [5]. This hypothesis is supported by the notion that, in frequently changing environments, food availability, access to mating partners and social contact may be problematic and induce the development of novel goal-directed behaviours [1, 3, 7–9]. In some species, younger animals may have less access to resources and consequently be driven to innovate more [2, 10]. Also, sex differences in innovation may reflect differences in needs [4]. For instance, when female chimpanzees are suckling offspring, they are in higher need for food than males and have been found to be more engaged than males in acquiring food and more persistent in the use of tools and the development of new food sources [11]. Male primates, however, experience higher competition for mating partners than females and are consistently more innovative than females in accessing new sexual partners and in improving their rank [10, 12, 13].

Conversely, *opportunity* can be a source of innovative behaviour [3–6, 14]. Opportunity to develop novel behaviours may be provided by superior abilities of individuals caused by differences in sex, age, social rank or temperament [12, 15], by frequent social contact permitting information transfer when animals live in large groups [16–18] or by rich and stable environments, allowing leisure to develop innovative behaviour [2, 4, 19, 20]. For example, in some species, older animals have more opportunity to innovate than younger animals, because they can rely on their greater experience, size and strength [12, 15]. Furthermore, birds in frequent social contact [16, 17] and living in stable social structures [18, 21] may have greater opportunity to innovate through information transfer. And finally, individuals living in appropriate environmental conditions, such as animals in suitable captive conditions, may develop innovative behaviour because their needs are covered and they have an excess of leisure [2, 4, 19, 20]. When all basic needs are covered, animals may find it rewarding to have the challenge of a new problem [22]. For instance, rhesus monkeys have been found to have a great interest in improving their puzzle-solving skills [23]. Furthermore, animals play more frequently when their basic needs are covered, and play can scaffold innovation [24]. Behaviours that were previously developed in play can be used—innovatively—to solve problems in a new context [25]. For instance, chimpanzees who previously had the opportunity to play with sticks were observed to be more innovative when they had to fish termites with a stick than those without such play experience [26].

A suitable range of conditions for studying whether animals innovate out of need or out of opportunity is offered by captive mules, donkeys and horses, just because their environment is strongly influenced by human management. For instance, the needs of domestic equids [27] for movement of up to 30 km a day [28], for foraging for 12 to 16 hours a day [28–30], and for social contact [29, 31–35] are not covered in many management systems, which potentially causes stress, reduced physical welfare [36] and mental imbalance [37]. Abnormal [38, 39] and depressive-like behaviour [27, 40] may occur. Individuals’ attempts to mitigate such problems may stimulate innovations associated with movement, foraging, and social contact [11, 41–43]. Animals may innovate out of need when the social environment is unstable and out of opportunity when it is stable [16–18, 21]. Finally, animals may innovate because of the opportunities from play and when human management covers their basic needs [24, 25, 27].

Furthermore, equids are a good model system to address the question of whether innovations are affected by individual characteristics. In horses, studies have found that younger animals learn faster, are more interested in new stimuli and more successful in learning than older animals [44–48]. Female horses were found to learn faster than males in an operant learning task [49], but not in social learning tasks [48]. Equid species and breed types differ in their learning abilities [46, 50–53]. Mules and donkeys appear to be more plastic in their learning ability than horses, with a superior performance found in a spatial cognition task [53, 54]. Mules were better than horses in learning to discriminate previously reinforced pairs of patterns among those that were not paired in the training phase [52]. Mules, donkeys and horses are highly social species [28, 29, 31–35] and have been found to learn faster when kept in groups than in individual housing [55, 56].

When studying innovative behaviour, most research to-date has elicited innovation by presenting novel problems to animals in captivity, often combined with rewarding novel solutions with food [4, 57], such as operating a novel feeding apparatus in horses [58]. However, experimental evidence may not reflect innovation seen in non-experimental contexts and typically experiments do not provide large datasets for studying individual and environmental effects on innovative behaviour. For this reason, we used crowd-sourcing methods when searching for cases where equids developed innovative behaviour in human management [41, 59].

Previous crowd sourcing studies have analysed the range of flexibility of animal problem-solving abilities [60], cognitive capacities in goats [61], play behaviour in dogs and horses [62], door opening techniques in horses [41], and the impact of training in dogs [63]. Several methods have been used. Some amassed reports written by bird [64, 65], primate [42, 66, 67], elephant [59], dog [63], horse [41, 68] and general wildlife [69] enthusiasts. Others searched journals for keywords such as “unusual” or “novel” [12, 42, 64–67]. A third approach is to ask trained personnel and researchers for contemporary reports. A fourth is to search the internet platform YouTube and Facebook for video material about rare animal behaviour, as applied in a study on human responses on tail chasing in dogs [70], play behaviour in dogs and horses [62], and door opening in horses [41]. If videos with unclear or manipulated content are excluded and lay person documentations are available which clearly demonstrate that films have not undergone any professional, postproduction editing, YouTube and Facebook videos can provide high quality, raw footage [62, 71].

Data collection of this kind runs the risk of compiling false or unrepresentative reports, thereby generating a biased dataset [65, 72, 73]. Responses may be biased by over representation of reports from highly motivated respondents [74], reports about socially desirable behaviour (implying a “clever animal”) or even the respondents’ moods [73]. However, the approach has advantages that may offset its deficiencies. It potentially provides a large data set of rare observations, which could not possibly be collected by a single research team engaged in experimentation [59, 62, 64, 70, 75]. A large sample size of independent observations increases the credibility of reports [59, 72], and the power of statistical analyses, especially when data such as pictures or videos are available [41, 62, 67, 70, 75], and if efforts are made to exclude reports that do not meet suitable standards as described in the method section [62, 65].

In the present study, we contacted equid owners and caretakers directly and via internet at the website https://innovative-behaviour.org and searched the internet platforms YouTube and Facebook for videos of equids showing novel behaviours [62, 70]. The resulting data were used to investigated (i) whether innovations differed across the contextual categories foraging, movement, sociality, play and comfort [11, 24, 25, 41–43]. We also investigated whether innovations (ii) differed with respect to the equids’ sex [10–13], age [2, 10, 12, 15], breed type and species [46, 50–54] and (iii) was affected by the equids’ management (single vs group housing, access to roughage feed, access to pasture, and social contact) [16–18, 20–23].

Our primary aim was to study whether innovations may be promoted by need or by opportunity [2, 5, 6, 12, 14]. In restricted conditions, we expected animals to innovate out of need, with relatively few goal-directed innovations, perhaps repeating these behaviours at high frequency [1, 3, 5, 7–13]. In unrestricted conditions, we expected innovation to arise out of opportunity, perhaps generating a high number of different types of innovative behaviour [20, 22, 24–26].

## Materials and methods

### Website and videos

Owners and caretakers were invited to report on “unusual”, novel behaviour in horses, mules, and donkeys, by means of a website we set up (https://innovative-behaviour.org), contacting potential responders via horse journals, Facebook, various private websites, and at conferences and public talks in Germany, Austria, France, Hungary, Switzerland, the U.K., and the U.S.A. Reports could be submitted in either English, German, or French. In the first phase (from July 2012 to April 2016), a questionnaire asked for reports of any kind of “unusual” behaviour, with no particular focal behaviour (https://innovative-behaviour.org/en/questionary_innovative_behaviour_in_horses). Subsequently, as in the original questionnaire a lot of horses were reported to be innovative by opening doors or gates, a more specific questionnaire [72] on door and gate opening behaviour in horses was developed, which invited a new group of people to report their animals’ behaviour using the same website. From May 2016 to February 2017, based on preliminary analysis of the reports submitted, we amplified the original questionnaire with more focussed questions on door and gate opening in equids (https://innovative-behaviour.org/en/Questionnaire_horses_that_open_doors_or_gates) (for full questionnaires see, S1 and S2 Appendices). The data collection was closed in April 2018. In collecting the reports on the effects of environmental and individual-level factors on “unusual” behaviour for the present study we used both questionnaires, ensuring that none of the reports in the general questionnaire were duplicated in the door and gate opening questionnaire. Detailed behaviour descriptions on door and gate opening behaviour in horses were published in a previous study [41]. In the present study we included door and gate opening behaviour for analysing causes for innovative behaviour in more detail.

In addition, “unusual” behaviours and environmental and individual specific aspects visible at video material were collected from the internet platforms YouTube and Facebook (list of links see, S1 Table) with the key words “clever”, “smart”, “unusual”, “play”, “open door”, “open gate”, “escape”, “run-away”, “horse”, “pony”, “donkey”, and “mule”. The complete dataset is available in the S1 Table)

### Cases and case selection

We found 632 reports, which collectively described or depicted 1011 innovative behaviours. Of these reports 254 came from the general questionnaire, 269 from the door opening questionnaire and 109 from the videos. Three independent observers, one professor and two bachelors in equine science, rated the 1011 described behaviours on whether they were “novel” and agreed in 89% (inter observer agreement: Cohen’s Kappa κ = 0.84). Contentious cases (N = 265) were excluded:

1. Behaviour (N = 4) not clearly visible at the videos.
2. Novel actions (N = 17) which were shown only once (i.e. when novel behaviour is observed only once it cannot be discerned whether the behaviours may have been displayed by chance rather than being a product of learning processes [76]).
3. Novel actions (N = 135) that may possibly have been learned through observing other equids (i.e. people reported that other equids in the same stable showed the same behaviour).
4. Actions (N = 109) which were not innovative because they were:
  - either reported to be about trained behaviour: people confirmed our questions of whether they trained the behaviour or reinforced the behaviour verbally or with food; or they were
  - behaviours frequently shown in equids [29, 30]: for example, horses defecating on piles, or horses jumping over fences and feeding on the grass on the other side of the fence;
  - possibly the result of reduced welfare, but useless for finding a solution for the underlying deficiency [27, 30]: for example, a horse showing repetitive, stereotypic behaviour when scraping the ground with a toy.

Finally, we analysed the remaining 746 cases, which derived from 434 sources: 141 from the general questionnaire (median = 1 behaviours/case, min. = 1, max. = 12), 190 from the door opening questionnaire (median = 2 behaviours/case, min. = 1, max. = 12) and 103 from videos (median = 1 behaviours/case, min. = 1, max. = 4).

### Animals

The animals were 434 domestic equids, comprising 3 mules, 4 donkey and 427 horses. The animals were 113 females, 242 castrated males, 24 uncastrated males, and 52 equids for which the sex could not discerned. The mean age at which equids were reported to have started showing the behaviour was 9 years (median, min = 0.5, max. 31). The horses were of various breeds which were summarised according to the breed types deployed in genetic studies [77, 78]: Thoroughbred horses (N = 5), Draught horses (N = 18), Arabian horses (N = 43), ponies (N = 59), and Warmblood horses (N = 280). In 22 cases the breed type was not reported or was not obviously visible in the videos. Animal characteristics which were not reported or clearly visible in the video were not considered for the analysis (S1 Table).

### Questionnaires

We used a quantitative–qualitative mixed questionnaire approach [79]. We asked three open questions, two semi-closed questions, and 24 closed questions in a semi-random order to prevent order biases in the responses [79]. Catch questions, which repeat issues demanded in the questionnaire in different wording and placed in unexpected sectors of the questionnaire, were included in both questionnaires to test the reliability of the reports [68]. Five questions on what equids did after opening doors and gates were not used for the present study.

One semi-open and three open questions were used to distinguish whether the reported behaviour was novel, and to assign behaviours designated as novel to behavioural categories (described below). We asked for the upload of pictures and videos of the behaviour and for descriptions, drawings or pictures of the behaviours and for further suggestions or questions regarding the project.

Eight further questions were used for filtering unsuitable or unreliable responses. Four direct and two catch questions asked whether the behaviour was demonstrated or reinforced by humans, and one question whether other individuals in the same stable showed the same behaviour before the focus animal. One question asked whether other individuals in the same stable showed the same behaviour after the focal animal demonstrated it the first time. Reports that contained positive responses to these questions were removed.

The following questions were used to answer the research questions of the present study. Four questions related to the management of the animals: a) were animals kept in single or group housing; b) did they receive limited or unlimited roughage; c) did the animals have daily access to pasture or only on a limited number of days per week; and d) were the animals in permanent or temporary contact with other equids. Three closed questions requested information on the number of behaviours the animals showed, the frequency of the reported behaviours, and whether the process of developing the behaviour was observed. Three questions asked the breed, sex and age of the particular equid. Finally, we asked the reporting persons for agreement with the use of their reports for scientific purposes and publications, for voluntary furnishing of their email address and for permission to use their email address for further enquiries.

The variables group/individual housing and permanent/temporary social contact can be disassociated in our dataset and, therefore, evaluated as separate factors, because: (i) in some group housing systems there are retreat areas for individual animals, whilst some owners offer otherwise group-housed horses individual sleeping areas, and (ii) some otherwise individually housed horses have permanent contact with other horses through fences or low barriers (for full questionnaires see S1 and S2 Appendices).

### Ethics statement

We obtained written informed consent from all persons who answered the questionnaire. On the website, all responders agreed to the anonymous publication of their written data, pictures, and videos for scientific purposes: reasonable requests for access to anonymous agreements can be obtained from the corresponding author. Only this respondent-agreed information on the equids was used for the present study.

Some videos were published on YouTube with a Creative Commons CC BY licence (https://support.google.com/youtube/answer/2797468?hl=en&ref_topic=2778546). They are available and can be used without any restriction. Other videos were published with the standard YouTube or Facebook license. These can be looked at and links can be forwarded without any restriction, which was the default setting for all uploads (see YouTube and Facebook Terms of Service: https://www.youtube.com/t/terms, Facebookhttps://www.facebook.com/legal/terms).

Videos from You tube and Facebook are shown in the study. Links are given for viewing the videos at the providers own web page, which is in line with the copy right terms of the providers. No human data is given in the study, and all procedures performed in the study involving human participants were approved by the institutional research committee at Nuertingen-Geislingen University and with the 1964 Helsinki declaration and its later amendments or comparable ethical standards.

### Behavioural categories

Innovations were assigned to five behavioural categories developed from the immediate, observable context in which the behaviour was shown. We classified the innovations as foraging, escape, social, comfort and play (see Table 1 for examples and frequencies).

**Table 1:** Numbers and examples for innovative behaviour in horses, of the behavioural categories foraging, escape, social, comfort and play, which were evaluated in the present study.

Categories innovative behaviour	foraging	escape	social	comfort	play
nos. cases	166	392	99	24	65
example 1	kick fruit tree to forage on fruits on the ground	untie ropes or halters which restrict movement	foal grooms its mother with a brush	pile straw in the middle of the stall and lay down on it	play fetch with sticks by throwing the stick themselves
example 2	open doors or containers for getting access to food	open locked doors and gates to run free	realse others by opening doors or gates and joining conspecifics thereafter	remove rags, leg protections, and saddles from their body by opening various fasteners	open doors, gates, halters and ropes without leaving afterwards
example 3	kick feeder so that food falls out	crawl through or underneath fences without destroying the fence or being hurt	throwing a feeding bucket in front of a person at feeding times	step on the edge of a tub and turn it upright for scratching at it	approach light switch and turn light on or off

Three examples are given for each category.

Behaviours were categorized as falling into four frequency classes, designated 1–4 as follows: 1 = 2–10 times (N = 161), 2 = 11–20 times (N = 70), 3 = more than 20 times (N = 216), and 4 = daily (N = 154). The frequency of occurrence was not reported for N = 145 behaviours.

### Data analysis

For statistical analysis and the depiction of the data R Studio (version 0.99.484, Boston MA, USA) of the R-Project statistical environment (R Development Core Team, 2018) and the package lme4 was used. Most of the data were not normally distributed (K-S test). We applied Generalised Linear Models (GLMs) to analysing the effect of the covariates and fixed effects ID of the equids (because some equids were repeated in the data set when displaying several novel behaviours), data source, behaviour category, sex, age, breed type or equid species, stabling, access to roughage feed, social contact, and access to pasture on the dependent variables “behaviour frequency” and “number of novel actions”. Random effects were not applicable as all the factors under consideration were not truly independent from the experimental design, i.e. they were collected for the questionnaire and did not arise by chance. The model with the best fit (the model with the lowest information loss versus the lowest clustering, quoted with the lowest Akaike information criterion, AIC) was chosen after stepwise removal of factors. Complete and reduced models are listed at the S3 Appendix.

## Results

### Innovators’ management conditions

Fifty-two percent of the equids showing novel behaviours (henceforth ‘innovative equids’) were kept in single box stabling, with the remainder (48%) in group housing. Comparable numbers of innovative equids received limited (51%) and unlimited roughage (49%) for food. Sixty-six percent of the innovative equids had daily access to pasture and thirty-four percent access restricted to a limited number of days. Sixty percent of the innovative equids were in permanent contact with other equids and forty percent in temporary contact.

### Frequency of repeating once-innovative behaviour

After the animals showed the innovation the first time, the frequency of repeating this behaviour was not affected by the data source the behaviours were collected from, by the equids’ sex, age, breed type or species, or whether they were stabled in single or group housing, had limited or unlimited access to roughage feed, or daily access to pasture or only on a limited number of days (GLM: N = 746, all p > 0.05).

Equids in unrestricted contact with others tended to repeat the behaviour at a higher frequency (mostly more than 20 times, frequency categories: median = 3, min. = 1 (2–10 times), max. = 4 (daily)) than those kept with restricted contact to other equids (mostly between 11 and 20 times, frequency category: median = 2, min. = 1 (2–10 times), max. = 4 (daily)).

### The number of different types of innovation reported per equid

**Fig 1:**
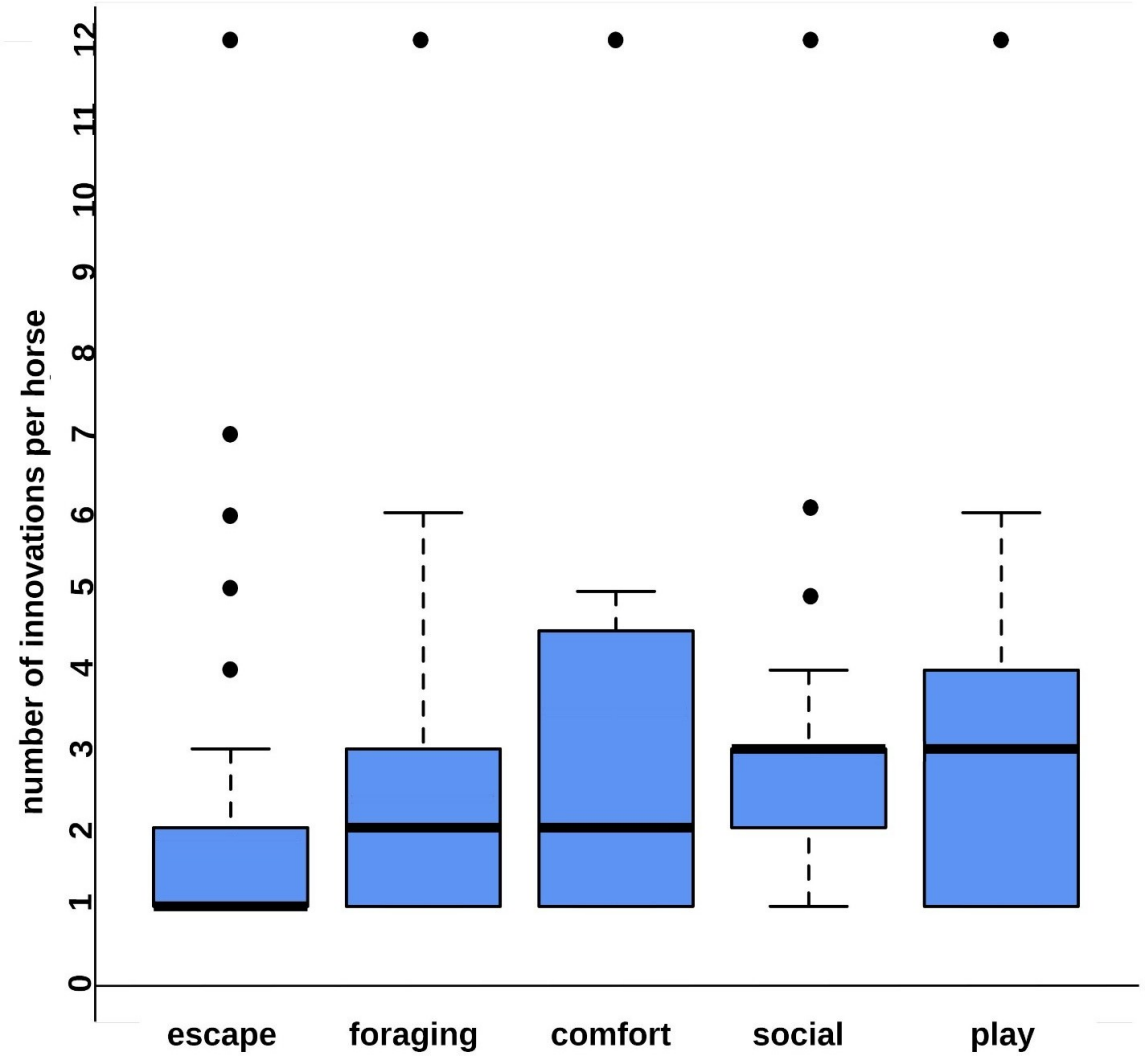
Number of types of innovations displayed per equid listed in the behaviour categories foraging, escape, social, comfort, and play. Each boxplot shows the quartiles of the number of innovations displayed per horse in each category. The box comprises 50% and the lower and upper whisker 25% of the variability each. The bold line provides the median, and the dots visualize outliers. Some equids displayed more than one innovation in a category but were considered in each behaviour category only once to avoid a bias in the median through replications.

The number of different types of novel behaviour shown per equid did not differ depending on the equids’ sex, age, breed type or species (GLM, N = 746, all p > 0.05). As expected, more different innovations per equid were reported in the general questionnaire than the door opening questionnaire and the videos (data see Method section; GLM: N = 746, SE = 0.25, z = -2.48, p = 0.01). The highest median number of innovations per equid was reported in the category play (N = 65 in 59 equids), followed by the category social (N = 99 in 91 equids), the category comfort (N = 24 in 19 equids), the category foraging (N = 166 in 151 equids), and the category escape (N = 392 in 328 equids) (GLM, N = 746, SE = 0.08, z = 2.89, p = 0.004; Fig 1).

### The number of reported innovations per equid and management condition

**Fig 2:**
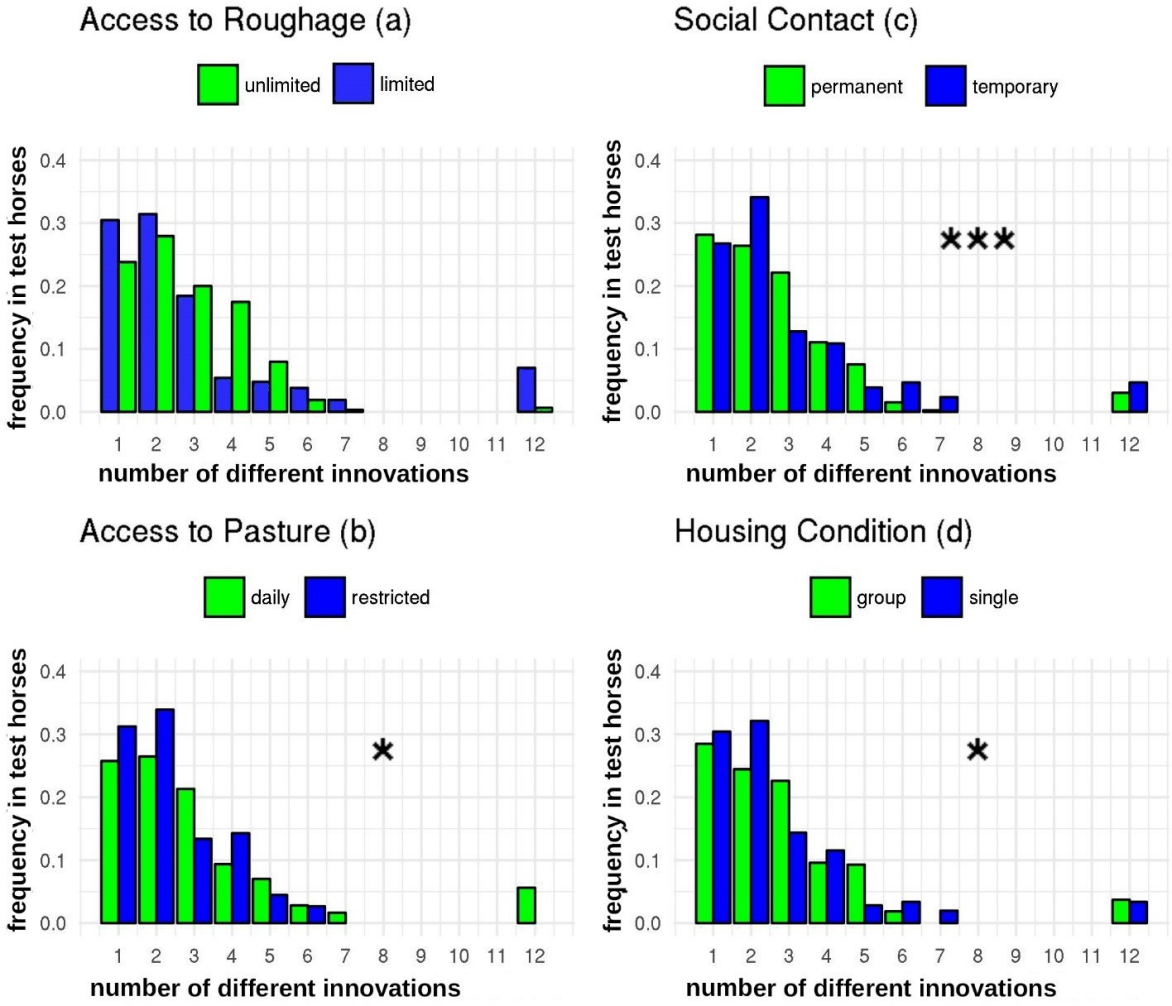
Diversity of innovative behaviour in four management conditions in equids. The x-axis shows the numbers of different types of innovations displayed by the equids and the y-axis the frequency of each number in the population, with 0 = 0% and 1 = 100% of the test animals. The green bars show unrestricted management conditions and the blue bars restricted management conditions for a) access to limited or unlimited roughage, b) access to pasture on a limited number of days per week or daily, c) permanent or temporary contact with other equids, and d) single or group housing. Statistical differences between the green and the blue bars are symbolised with * = p < 0.05 and *** = p < 0.001 in graph b, c, and d.

Equids displayed a greater number of different types of innovation when they were kept in group housing than in single housing (GLM: N = 746, SE = 0.22, z = 2.13, p = 0.03; Fig 2).

Amongst innovative equids, those that had daily access to pasture exhibited more types of novel behaviour than those with access to pasture restricted to a limited number of days (GLM: N = 746, SE = 0.25, z = 2.12, p = 0.03; Fig 2).

Amongst innovative equids, those fed with unlimited roughage tended to display more types of novel behaviour than those with limited access to roughage, but this was only a trend and not significant (GLM: N = 746, SE = 0.17, z = 0.25, p = 0.8; Fig 2).

Finally, those innovative equids that had permanent social contact exhibited fewer distinct types of innovations than those restricted to temporary social contact (GLM: N = 746, SE = 0.28, z = -5.61, p < 0.001; Fig 2).

## Discussion

Crowd-sourcing methods were used to build a large database of innovation in equids from questionnaires, pictures and videos. The analysis of the resulting data reflected the development of few, goal-directed innovations in equids under human generated restrictions in basic needs for food, free movement and sociality [27, 29, 30, 35]. Conversely, animals that enjoyed rather unrestricted conditions [27] developed a higher number of different types of innovations [2, 19, 20]. Finally, in management conditions that imposed extensive social contact [2, 10, 12, 13] the animals again exhibit specific, goal-directed innovations.

### Does high frequency of repeating behaviours indicate innovation out of need?

In single box housing, equids show frequent repetition of a few goal directed, once-innovative behaviours for escape and foraging. Especially single box housing conditions do not meet the equids basic needs [29, 30, 36]. Therefore, the few innovations may be a manifestation of an unexpressed need for movement and foraging [27].

Equids also displayed a low number of different types of innovations, when they were in permanent social contact (i.e. they performed few innovations repeatedly, Fig 2) compared to temporary social contact, although often they showed these behaviours at high frequency. That this elevated frequency of repeating behaviour is not observed in group housed equids, nor across animals in general, and that social facilitation has not been found to be a driver of innovation [4], imply that it is some aspect of social confrontation rather that housing regime or learning style that is the crucial variable. The most obvious factor is the competition for resources that can arise amongst equids that experience high levels of social interaction. Plausibly, in permanent social contact the animals may have been driven by need to develop innovations out of conflict or competition over resources such as food, water, or mating partners, as has been reported in several other species [5, 7–9].

Innovations in a foraging context have been reported to be more common when animals were in need of food, especially in experimental set ups where goal-directed development of innovations was studied under controlled conditions [3, 57]. In the diverse environments of the present study we found no clear differences in the frequency of goal-directed foraging innovations between animals kept with unlimited or restricted food.

### Does high variety of innovations indicate innovation out of opportunity?

Equids showed a higher number of different types of innovation in behavioural contexts of comfort, social, and play than in escape and foraging. All basic needs [27] may have been covered in the former, and opportunity may have been the driving force for developing a variety of non-goal directed innovations [3–6, 14]. The opportunity to play may have promoted innovation [24, 25], as in chimpanzees who used sticks for play and termite fishing [26]. Furthermore, opportunity in stable social structures [18, 21] of highly social equids [28, 32–34, 54] and a surplus of leisure, in which the mind is not occupied by finding solutions to an undesirable situation, may have promoted a variety of innovations [2–6, 14, 16, 17, 19, 20].

The greatest number of different types of innovation was shown by animals kept in the unrestricted conditions, with unlimited access to pasture, access to roughage and group housing. This observation meshes with findings that housing and handling qualities can affect horses’ behavioural responses and emotionality. In a previous study [37], horses kept in group housing with low workloads and unlimited feed were found to respond to human voice recordings more positive compared to horses kept in individual boxes with a high work load and limited feed [37, 80–82]. A positive emotional state in group housed horses was reflected in enhanced electrophysical activity in the left brain hemisphere—a brain region thought to be activated when animals respond to positive emotions—and by the horses’ ear positions which are said to reflect positive emotions, such as holding ears forward [37]. In contrast, horses in individual boxes with a high work load and limited feed showed enhanced right brain hemisphere activation—a brain region thought to be activated when animals respond to negative emotions—and they held their ears backwards, believed to reflect negative emotions [37].

### Effect of sex, age, breed type and equid species on the variety of innovations and the frequency of repetition

In contrast to previous studies [4], we observed no effect of the equid’s sex on the number of different types of innovation or on the frequency of repeating these behaviours. The mix of equids coming from conditions in which basic needs were not fulfilled [27] and those coming from conditions in which they were fulfilled may have inflated the variance of the data in such a way that it obscured any weak effects of sex in the present study. Sex was found to affect individual learning in several species including horses [49], such as for food acquisition [11, 61], the acquisition of mating partners, and for rank improvement [10, 12, 13]. However, no sex differences were reported in a social learning study in which horses were kept in open stabling, in long-lived, stable social structures [48], which may promote innovation out of opportunity [18, 21].

Any effect of age may plausibly have been negated in the present study by the conflicting tendencies of young horses to display high learning speed and interest in new stimuli [44–48], and the enhanced innovativeness of old animals that arises through experience [12]. However, in common with the general pattern in the innovation literature [4], innovative equids in the present study tended to be older, with a maximum age of 31 years, for example, when compared to horses, which learned socially, with a maximum age of 14 years [48].

We observed no effect of the equid’s breed or species on the frequency or the number of different types of innovation. Various breed or species effects on learning and innovativeness reported in previous studies may have counteracted each other [44, 38, 83]. For example, Warmblood horses, were found to be more successful in finding innovative solutions in an operant feeding task than other horses breed types [58]. However, donkeys and mules displayed higher plasticity in their cognitive abilities than horses [52–54], but showed poorer motivation in learning tasks [52].

Testing the effect of further individual factors on animals’ decisions to innovate was out of the scope of this crowd-sourcing study but should be considered for follow up studies. Especially, the animals’ personality is likely to impact innovativeness, as more neophilic, explorative, and active animals displayed more innovative behaviour all through animal innovation literature [4, 84] and in an experimental study in horses [58].

### Potential biases in the data

However, it is important to acknowledge potential biases in our data. Raising data by crowd sourcing potentially causes biases in the dataset. We took care to exclude single cases and unreliable or biased reports, especially reports on demonstrated or reinforced (i.e. trained) behaviour [62, 65], and supported the reports with pictures and videos [62, 67, 70, 75]. This allowed us to amass a large data set of sufficiently replicated, rare observations [72] and for analysing the influence of several factors on innovations [41, 59, 62, 64, 69, 70, 75]. A major contribution of this study is to set up this database, which can then be subject to more fine-grained analyses and attempts to control for potential confounds in future studies.

Furthermore, it is important to acknowledge the limitations of these methods, as they may affect interpretations. Where we find that a factor covaries with innovation frequency or the number of different innovation types, that could be a genuine finding, or an artefact generated by bias in the respondent’s behaviour or reporting. For instance, the greater apparent number of innovations observed in the context of comfort than escape may be a genuine finding. It may also reflect a biased tendency of respondents keeping equids in unrestricted management conditions where comfort can be increased by innovation. Furthermore, bias may arise because respondents were more enthusiastic to detect and report innovations causing increases in the equids comfort, relative to equid owners keeping their horses that promoted their escape. In all behaviour categories, equid owners may unintentionally have reinforced (i.e. trained) the behaviour by rewarding the animal with enhanced affection [73]. Therefore, the present study applied two direct and two catch questions [68] for filtering reports of trained behaviour, but unintentional reinforcement might not have been obvious in all cases. In addition, any such potential bias may have been generated by equid owners reporting door and gate opening: behaviour in an escape context that may exhibit low diversity in form.

This is particularly relevant to variables such as the reported management conditions. We expected a disproportionate number of equids reported at the web site to be kept under particularly good conditions. Because owners interested in the mental welfare of their horses (i.e. in keeping a socially desirable item, a “clever animal” [73]) are presumed to be more aware of good management conditions and may have been more motivated to respond to our request [74]. Access to data on management conditions amongst all equid owners would have been ideal. This would allow statistical analyses that quantified the level of bias. While that was not possible for the current study, it remains a possibility for future studies.

Lastly, we note that our request for reports was more open in the general questionnaire and more precise in the questionnaire asking for door and gate opening [41]. Inevitably, this resulted in a greater variety of reported unusual behaviour in the general questionnaire than in the door and gate opening questionnaire and the videos [72], which mostly only documented a single innovation. Naturally, equids may have been capable of further innovations that were not documented because owners responded to a specific request at the door and gate opening questionnaire [41] or documented a single case of behaviour via video.

Whether such biases exist, and their magnitude, is currently unknown; we therefore take our findings at face value and provide the interpretations that would be appropriate were the dataset to be unbiased. However, we stress that, until such a time as the level of bias can be quantified, our findings should be regarded as provisional and suggestive rather than definitive.

## Conclusion

Our study reveals an interesting disconnect between the frequency of repeating once-innovative behaviour and the numbers in types of innovation exhibited by equids: with the former potentially indicative of innovation in response to need, and the latter indicative of innovation resulting from opportunity. Equids displayed a restricted range of apparently goal directed innovations and repeated them at high frequency in circumstances of need, such as for escape and foraging and when the management was restricted or imposed conflict. Conversely, equids showed a greater number of innovation types when they had the opportunity to display behaviour related to comfort, play or social behaviour, and when kept in unrestricted management conditions with little conflict. It remains to be shown whether this variety of innovative behaviour arises in favourable environments in other species.

## Supporting information

S1 Appendix Questionnaire innovative behaviour. (PDF)

S2 Appendix Questionnaire: Horses that open doors or gates. (PDF)

S3 Appendix Statistical data. Complete GLM models and reduced GLM models with lowest AIC.
(PDF)

S1 Table Data per horse. (PDF)
