# Supplementary material for: Need or opportunity? A study of innovations in equids
Source: PLoS One. 2021 Sep 27;16(9):e0257730. doi: 10.1371/journal.pone.0257730 (PMC8476013; doi:10.1371/journal.pone.0257730)
Supplement: S2 Appendix — (PDF) [file pone.0257730.s002.pdf]

Questionnaire: horses that open doors or gates

Language
English

Thank you for you interest in our online survey.

You can take part in this survey anonymously or under your name.

If you choose to complete the questionnaire anonymously, you will not be able to make changes after you have submitted it.

If you would like to have the option to make additions or changes later, please create a log-in and an account in the block on the right of this page.

With the general questionnaire on innovative behaviour 85 descriptions of horses opening doors or gates have already reached us. We would like to know as much about door or gate opening abilities as possible. We would love to find out whether horses show similarities in their techniques, or whether they develop individual methods. Could you do us a great favour and answer the following questions?

For horses that show other clever behaviours but do not open doors, please tell us about these horses at the “innovative behavior questionnaire”.

Thank you for participating in our online survey.

- The term "horse" is used for all equids (mules, donkeys and zebras) in the following questionnaire. Details of the breed can be given in the breed question or comment field.
- Please describe the behaviour of **one** horse. If the behaviour is shown by several horses, it can be specified in a later section of the questionnaire.
- Please note that all questions marked with a * need to be answered to proceed.

Which style of management does the horse have? *

- ○ group housing
- ○ single box stabling
- ○ unknown

Is the horse turned out so it can move freely on a pasture or paddock? *

- ○ no
- ○ Access to pasture / paddock is restricted
- ○ Permanent access to pasture / paddock
- ○ unknown

Is the horse turned out with at least one other horse? *

- ○ yes
- ○ no
- ○ unknown

How much roughage does your horse get daily? *

- Access to roughage is restricted
- Permanent access to roughage
- unknown

How often have you observed your horse opening doors or gates? *

- once
- 2-10 times
- 11-20 times
- more than 20 times
- daily
- unknown

Do other horses in the stable open doors or gates similarly to your horse? *

- no
- yes, one other horse
- yes, more than one other horse
- unknown

Did you show the horse how to open door or gate mechanisms? *

- yes
- no
- unknown

Did other horses start opening doors or gates after observing your horse? *

- no
- yes, one other horse
- yes, more than one other horse
- unknown

Are you aware of anyonebody else who mightay have shown the horse how to open doors or gates? *

- (x) yes
- no
- unknown

Can you describe how you or another person showed it to the horse?

Have you or anyone else given the horse food or any other reward after it opened a door/gate? *

- yes
- no
- unknown

Have you watched the whole process of trying to open doors or gates until the horse was finally successful *

- yes
- no
- unknown

Do you enjoy watching your horse opening doors or gates? *

- yes
- no
- unknown

Does your horse open one door or gate mechanism, or several different types?? *

- one mechanism
- several mechanisms
- unknown

Do you secure the mechanisms? *

- yes
- no
- unknown

Could you describe, draw or upload pictures of the gate or door mechanism or mechanisms the horse is able to open? *

Please feel free to attach pictures or clips

Add a new file

Browse no file selected Upload

What happens when your horse opens locks?

Does it stay in the stable? *

- yes
- no
- unknown

Does it visit other horses? *

- yes
- no
- unknown

Does it move around freely? *

- yes
- no
- unknown

Does it break in into other places? *

- yes
- no
- unknown

Does it free other horses? *

- yes
- no
- unknown

Sex *

- mare
- gelding
- stallion
- unknown

Can you tell the age of the horse (when observed)? *

- Select -

Which breed is the horse? *

- Select -

e-mail *

Do you have any suggestions or questions regarding our project, innovative behaviour in general, or your observations?

I agree *

- to the use of my the information provided on this questionnaire for the project on observation and analysis of innovative behaviour in horses at the Nürtingen University (HfWU Nürtingen-Geislingen). I agree to, the publication of this information in journals in anonymized form
- to the use of the information for the project on observation and analysis of innovative behaviour in horses at the Nürtingen University (HfWU Nürtingen-Geislingen).But the Information should not be given to others.

The Information will not be given to others.

Use of e-mail address *

- I permit my e-mail address to be used for further inquiries concerning this project.
- I do not permit my e-mail address to be used for further inquiries concerning this project.

None of your information will be given to others.
