# Supplementary material for: Need or opportunity? A study of innovations in equids
Source: PLoS One. 2021 Sep 27;16(9):e0257730. doi: 10.1371/journal.pone.0257730 (PMC8476013; doi:10.1371/journal.pone.0257730)
Supplement: S3 Appendix — Complete GLM models and reduced GLM models with lowest AIC. (PDF) [file pone.0257730.s003.pdf]

Supporting Information S3 Appendix

Need or opportunity? A study of innovative behaviour in equids.

Konstanze Krueger[Δ*], Laureen Esch[+Δ], Richard Byrne[•], Kevin Laland[⌂]

[Δ] Nuertingen-Geislingen University, Faculty Agriculture, Economics and Management, Department Equine Economics, Neckarsteige 6-10, 72622 Nürtingen, Germany

* University of Regensburg, Zoology / Evolutionary Biology, Universitätsstraße 31, 93053 Regensburg, Germany

[+] Ludwig Maximilian University Munich, Veterinarian Medicine, Department of animal welfare, ethology, animal hygiene and animal husbandry, Veterinärstr. 13/R, 80539 München, Germany

[•] Centre for Social Learning & Cognitive Evolution, School of Psychology, University of St Andrews, St Andrews, Fife, KY16 9JP, Scotland, UK

[⌂] School of Biology, University of St Andrews, St Andrews, Fife, KY16 9TF, Scotland, UK

* Corresponding author:

Konstanze Krueger,

Konstanze.Krueger@hfwu.de

tel: 0049 7022 201 331

Statistical Data, complete GLM models + reduced models (lowest AIC)

Full GLM: dependent variable behavior frequency, sheet behaviours

```
Call:
glm(formula = behaviour.frequency ~ video_quest_num + sID + age. +
    behaviour_category + breed.type.num + purpose.of.domestication +
    restricted...unrestricted..roughage + restricted...unrestricted.contact.with.horses +
    restricted...unrestricted.Pasture + single.group.stabling +
    sex_num, family = poisson(identity), data = Dataset1)

Deviance Residuals:
     Min        1Q    Median        3Q       Max
-1.30400  -0.55407   0.09546   0.38707   1.13709

Coefficients:
                                              Estimate Std. Error z value  Pr(>|z|)
(Intercept)                                  7.5776465  1.8634712   4.066 0.0000477 ***
video_quest_num                              0.1965641  0.2947378   0.667   0.50483
sID                                         -0.0017541  0.0008991  -1.951   0.05106 .
age.                                         0.0075429  0.0152756   0.494   0.62146
behaviour_category                           0.0022544  0.0899545   0.025   0.98001
breed.type.num                               0.0449593  0.0988250   0.455   0.64915
purpose.of.domestication                    -0.2820275  0.1019212  -2.767   0.00566 **
restricted...unrestricted..roughage         -0.1855316  0.2017316  -0.920   0.35773
restricted...unrestricted.contact.with.horses  0.7392814  0.3058808   2.417   0.01565 *
restricted...unrestricted.Pasture            0.1604589  0.2993294   0.536   0.59192
single.group.stabling                       -0.3633528  0.2744061  -1.324   0.18546
sex_num                                     -0.2165500  0.1833697  -1.181   0.23762
---
Signif. codes:  0 '***' 0.001 '**' 0.01 '*' 0.05 '.' 0.1 ' ' 1

(Dispersion parameter for poisson family taken to be 1)

    Null deviance: 153.12  on 389  degrees of freedom
Residual deviance: 123.14  on 378  degrees of freedom
  (124 observations deleted due to missingness)
AIC: 1359.5
```

Reduced GLM - lowest AIC, dependent variable behavior frequency, sheet behaviours

```
Call:
glm(formula = behaviour.frequency ~ sID + age. + purpose.of.domestication +
    restricted...unrestricted..roughage + restricted...unrestricted.contact.with.horses +
    restricted...unrestricted.Pasture + sex_num, family = poisson(identity),
    data = Dataset1)

Deviance Residuals:
    Min       1Q   Median       3Q      Max
-1.3138  -0.5256   0.1100   0.4109   1.1713

Coefficients:
                                              Estimate Std. Error z value   Pr(>|z|)
(Intercept)                                  7.5187740  1.6369665   4.593 0.00000437 ***
sID                                         -0.0015288  0.0006619  -2.310    0.02091 *
age.                                         0.0084962  0.0152692   0.556    0.57792
purpose.of.domestication                    -0.2753410  0.1000149  -2.753    0.00591 **
restricted...unrestricted..roughage         -0.2348661  0.1976762  -1.188    0.23478
restricted...unrestricted.contact.with.horses  0.5125995  0.2529876   2.026    0.04275 *
restricted...unrestricted.Pasture            0.0660098  0.2859036   0.231    0.81741
sex_num                                     -0.2367565  0.1827523  -1.296    0.19515
---
Signif. codes:  0 '***' 0.001 '**' 0.01 '*' 0.05 '.' 0.1 ' ' 1

(Dispersion parameter for poisson family taken to be 1)

    Null deviance: 153.12  on 389  degrees of freedom
Residual deviance: 125.11  on 382  degrees of freedom
  (124 observations deleted due to missingness)
AIC: 1353.4

Number of Fisher Scoring iterations: 5
```

Full GLM: dependent variable Nr. reported behaviours, sheet behaviours

```
Call:
glm(formula = nr_reported_behaviours ~ video_quest_num + sID +
    age. + behaviour_category + breed.type.num + purpose.of.domestication +
    restricted...unrestricted..roughage + restricted...unrestricted.contact.with.horses +
    restricted...unrestricted.Pasture + single.group.stabling +
    sex_num, family = poisson(identity), data = Dataset1)

Deviance Residuals:
    Min       1Q   Median       3Q      Max
-2.0458  -0.9079  -0.1511   0.5381   3.1380

Coefficients:
                                              Estimate Std. Error z value     Pr(>|z|)
(Intercept)                                   7.324535   1.530249   4.786 0.0000016972 ***
video_quest_num                              -0.596111   0.246206  -2.421      0.01547 *
sID                                          -0.001759   0.000727  -2.420      0.01551 *
age.                                         -0.004099   0.013550  -0.302      0.76227
behaviour_category                            0.228412   0.079831   2.861      0.00422 **
breed.type.num                               -0.046275   0.075588  -0.612      0.54040
purpose.of.domestication                      0.129731   0.086055   1.508      0.13167
restricted...unrestricted..roughage           0.029581   0.173211   0.171      0.86440
restricted...unrestricted.contact.with.horses -1.557528   0.276533  -5.632 0.0000000178 ***
restricted...unrestricted.Pasture             0.544131   0.248024   2.194      0.02825 *
single.group.stabling                         0.499904   0.226353   2.209      0.02721 *
sex_num                                       0.189173   0.160745   1.177      0.23925
---
Signif. codes:  0 '***' 0.001 '**' 0.01 '*' 0.05 '.' 0.1 ' ' 1

(Dispersion parameter for poisson family taken to be 1)

    Null deviance: 539.80  on 410  degrees of freedom
Residual deviance: 432.87  on 399  degrees of freedom
  (103 observations deleted due to missingness)
AIC: 1594.4

Number of Fisher Scoring iterations: 12
```

reduced GLM - lowest AIC: dependent variable Nr. reported behaviours, sheet behaviours

```
Call:
glm(formula = nr_reported_behaviours ~ video_quest_num + sID +
    age. + behaviour_category + purpose.of.domestication + restricted...unrestricted.contact.with.horses +
    restricted...unrestricted..roughage + restricted...unrestricted.Pasture +
    single.group.stabling + sex_num, family = poisson(identity),
    data = Dataset1)

Deviance Residuals:
    Min       1Q   Median       3Q      Max
-2.0556  -0.9110  -0.1430   0.5388   3.1481

Coefficients:
                                               Estimate Std. Error z value     Pr(>|z|)
(Intercept)                                   7.3413486  1.5309505   4.795 0.0000016244 ***
video_quest_num                              -0.6095195  0.2459738  -2.478      0.01321 *
sID                                          -0.0017736  0.0007251  -2.446      0.01444 *
age.                                         -0.0048097  0.0135181  -0.356      0.72199
behaviour_category                            0.2309408  0.0798652   2.892      0.00383 **
purpose.of.domestication                      0.1298351  0.0866419   1.499      0.13400
restricted...unrestricted.contact.with.horses -1.5514752  0.2765572  -5.610 0.0000000202 ***
restricted...unrestricted..roughage           0.0430476  0.1721454   0.250      0.80254
restricted...unrestricted.Pasture             0.5223906  0.2469190   2.116      0.03438 *
single.group.stabling                         0.4794488  0.2249118   2.132      0.03303 *
sex_num                                       0.1898768  0.1605780   1.182      0.23702
---
Signif. codes:  0 '***' 0.001 '**' 0.01 '*' 0.05 '.' 0.1 ' ' 1

(Dispersion parameter for poisson family taken to be 1)

    Null deviance: 539.80  on 410  degrees of freedom
Residual deviance: 433.26  on 400  degrees of freedom
  (103 observations deleted due to missingness)
AIC: 1592.8

Number of Fisher Scoring iterations: 12
```

reduced GLM – lowest AIC: dependent variable Nr. reported behaviours, nested in data source (video … quest), sheet behaviours

```
Call:
glm(formula = nr_reported_behaviours ~ (sID + age. + behaviour_category +
    purpose.of.domestication + restricted...unrestricted.contact.with.horses +
    restricted...unrestricted..roughage + restricted...unrestricted.Pasture +
    single.group.stabling + sex_num) %in% video...quest, family = poisson(identity),
    data = Dataset1)

Deviance Residuals:
    Min       1Q   Median       3Q      Max
-2.3331  -0.7815  -0.1082   0.5112   2.4722

Coefficients:
                                                                    Estimate Std. Error z value Pr(>|z|)
(Intercept)                                                       10.5903284  1.6575703   6.389 1.67e-10 ***
sID:video...questquest                                            -0.0020485  0.0007356  -2.785  0.00535 **
sID:video...questquest_door                                       -0.0044841  0.0007886  -5.686 1.30e-08 ***
age.:video...questquest                                           -0.0428491  0.0168767  -2.539  0.01112 *
age.:video...questquest_door                                       0.0171147  0.0201670   0.849  0.39608
behaviour_category:video...questquest                              0.1502188  0.1018770   1.475  0.14034
behaviour_category:video...questquest_door                         0.1241687  0.1125211   1.104  0.26980
purpose.of.domestication:video...questquest                       -0.2918442  0.1090708  -2.676  0.00746 **
purpose.of.domestication:video...questquest_door                   0.3972042  0.1387394   2.863  0.00420 **
restricted...unrestricted.contact.with.horses:video...questquest      -2.6753117  0.4142342  -6.458 1.06e-10 ***
restricted...unrestricted.contact.with.horses:video...questquest_door -1.4849299  0.4773097  -3.111  0.00186 **
restricted...unrestricted..roughage:video...questquest            -0.1915179  0.2469935  -0.775  0.43811
restricted...unrestricted..roughage:video...questquest_door        0.7891742  0.2544358   3.102  0.00192 **
restricted...unrestricted.Pasture:video...questquest               1.7980262  0.4347830   4.135 3.54e-05 ***
restricted...unrestricted.Pasture:video...questquest_door         -0.5130901  0.2892747  -1.774  0.07611 .
single.group.stabling:video...questquest                          -0.3139499  0.2543837  -1.234  0.21714
single.group.stabling:video...questquest_door                      1.9376424  0.4727323   4.099 4.15e-05 ***
sex_num:video...questquest                                         0.0273719  0.2029780   0.135  0.89273
sex_num:video...questquest_door                                   -0.4011968  0.2495297  -1.608  0.10788
---
Signif. codes:  0 ‘***’ 0.001 ‘**’ 0.01 ‘*’ 0.05 ‘.’ 0.1 ‘ ’ 1
```

```
(Dispersion parameter for poisson family taken to be 1)

    Null deviance: 539.80  on 410  degrees of freedom
Residual deviance: 349.05  on 392  degrees of freedom
  (103 observations deleted due to missingness)
AIC: 1524.5
```

reduced GLM - lowest AIC: dependent variable Nr. reported behaviours, nested in behavour category, sheet behaviours

```
Call:
glm(formula = nr_reported_behaviours ~ (video_quest_num + sID +
    age. + purpose.of.domestication + restricted...unrestricted.contact.with.horses +
    restricted...unrestricted..roughage + restricted...unrestricted.Pasture +
    single.group.stabling + sex_num) %in% behaviour_category,
    family = poisson(identity), data = Dataset1)

Deviance Residuals:
    Min       1Q   Median       3Q      Max
-2.0396  -0.7913  -0.1132   0.4974   3.3824

Coefficients:
                                                              Estimate Std. Error z value Pr(>|z|)
(Intercept)                                                  7.6923812  1.6059822   4.790 1.67e-06 ***
video_quest_num:behaviour_categorycomfort                   10.6428149  3.1262236   3.404 0.000663 ***
video_quest_num:behaviour_categoryescape                    -1.0266189  0.3169467  -3.239 0.001199 **
video_quest_num:behaviour_categoryfeeding                    0.6231075  0.4927509   1.265 0.206033
video_quest_num:behaviour_categoryplay                      -1.5122243  0.9252456  -1.634 0.102174
video_quest_num:behaviour_categorysocial                    -0.3902221  0.8071824  -0.483 0.628785
sID:behaviour_categorycomfort                               -0.0066749  0.0020989  -3.180 0.001471 **
sID:behaviour_categoryescape                                -0.0015139  0.0008123  -1.864 0.062376 .
sID:behaviour_categoryfeeding                               -0.0020589  0.0009406  -2.189 0.028610 *
sID:behaviour_categoryplay                                  -0.0022209  0.0013778  -1.612 0.106973
sID:behaviour_categorysocial                                -0.0019168  0.0014509  -1.321 0.186444
age.:behaviour_categorycomfort                              -0.0975195  0.1378480  -0.707 0.479292
age.:behaviour_categoryescape                               -0.0115431  0.0177494  -0.650 0.515474
age.:behaviour_categoryfeeding                              -0.0307194  0.0245626  -1.251 0.211059
age.:behaviour_categoryplay                                  0.1278228  0.0711877   1.796 0.072562 .
age.:behaviour_categorysocial                                0.0088841  0.0496427   0.179 0.857968
purpose.of.domestication:behaviour_categorycomfort           0.1366140  0.7162245   0.191 0.848728
```

```
purpose.of.domestication:behaviour_categoryescape                         0.1963969  0.1163338   1.688 0.091369 .
purpose.of.domestication:behaviour_categoryfeeding                       -0.1286774  0.1709093  -0.753 0.451511
purpose.of.domestication:behaviour_categoryplay                           0.6624964  0.3837817   1.726 0.084306 .
purpose.of.domestication:behaviour_categorysocial                         0.1621007  0.2867746   0.565 0.571901
restricted...unrestricted.contact.with.horses:behaviour_categorycomfort -4.0627965  1.5000274  -2.708 0.006759 **
restricted...unrestricted.contact.with.horses:behaviour_categoryescape  -1.1604146  0.3809264  -3.046 0.002317 **
restricted...unrestricted.contact.with.horses:behaviour_categoryfeeding -0.8881785  0.6463646  -1.374 0.169406
restricted...unrestricted.contact.with.horses:behaviour_categoryplay    -2.0337724  1.0183895  -1.997 0.045820 *
restricted...unrestricted.contact.with.horses:behaviour_categorysocial  -1.7023928  0.7395914  -2.302 0.021346 *
restricted...unrestricted..roughage:behaviour_categorycomfort           -1.5754269  1.2206027  -1.291 0.196809
restricted...unrestricted..roughage:behaviour_categoryescape             0.1807202  0.2263612   0.798 0.424655
restricted...unrestricted..roughage:behaviour_categoryfeeding            0.2094799  0.3597500   0.582 0.560369
restricted...unrestricted..roughage:behaviour_categoryplay               0.5564003  0.7478706   0.744 0.456889
restricted...unrestricted..roughage:behaviour_categorysocial            -0.2776571  0.5822727  -0.477 0.633469
restricted...unrestricted.Pasture:behaviour_categorycomfort              5.0089564  1.8597160   2.693 0.007073 **
restricted...unrestricted.Pasture:behaviour_categoryescape               0.2730010  0.3103033   0.880 0.378974
restricted...unrestricted.Pasture:behaviour_categoryfeeding              0.2449627  0.7680798   0.319 0.749781
restricted...unrestricted.Pasture:behaviour_categoryplay                 0.4761120  0.9950268   0.478 0.632300
restricted...unrestricted.Pasture:behaviour_categorysocial              -0.0023600  0.7713149  -0.003 0.997559
single.group.stabling:behaviour_categorycomfort                         -2.5296701  1.3047588  -1.939 0.052525 .
single.group.stabling:behaviour_categoryescape                           0.3865593  0.3098427   1.248 0.212178
single.group.stabling:behaviour_categoryfeeding                         -0.1457524  0.5057278  -0.288 0.773191
single.group.stabling:behaviour_categoryplay                             0.9956029  0.7807344   1.275 0.202234
single.group.stabling:behaviour_categorysocial                           1.1061130  0.6393228   1.730 0.083607 .
sex_num:behaviour_categorycomfort                                        2.2580607  1.3502979   1.672 0.094471 .
sex_num:behaviour_categoryescape                                        -0.0421517  0.2022829  -0.208 0.834932
sex_num:behaviour_categoryfeeding                                        0.2911486  0.3475627   0.838 0.402207
sex_num:behaviour_categoryplay                                          -0.0954294  0.6863423  -0.139 0.889418
sex_num:behaviour_categorysocial                                         0.7455583  0.5364538   1.390 0.164593
---
Signif. codes:  0 ‘***’ 0.001 ‘**’ 0.01 ‘*’ 0.05 ‘.’ 0.1 ‘ ’ 1

(Dispersion parameter for poisson family taken to be 1)

    Null deviance: 539.80  on 410  degrees of freedom
Residual deviance: 354.68  on 365  degrees of freedom
  (103 observations deleted due to missingness)
AIC: 1584.2
```
