# Supplementary material for: Need or opportunity? A study of innovations in equids
Source: PLoS One. 2021 Sep 27;16(9):e0257730. doi: 10.1371/journal.pone.0257730 (PMC8476013; doi:10.1371/journal.pone.0257730)
Supplement: S1 Table — (PDF) [file pone.0257730.s004.pdf]

Supporting Information, S1 Table

Need or opportunity? A study of innovative behaviour in equids.

Konstanze Krueger[Δ]*, Laureen Esch[+Δ], Richard Byrne[•], Kevin Laland[⌂]

[Δ] Nuertingen-Geislingen University, Faculty Agriculture, Economics and Management, Department Equine Economics, Neckarsteige 6-10, 72622 Nürtingen, Germany

* University of Regensburg, Zoology / Evolutionary Biology, Universitätsstraße 31, 93053 Regensburg, Germany

[+] Ludwig Maximilian University Munich, Veterinarian Medicine, Department of animal welfare, ethology, animal hygiene and animal husbandry, Veterinärstr. 13/R, 80539 München, Germany

[•] Centre for Social Learning & Cognitive Evolution, School of Psychology, University of St Andrews, St Andrews, Fife, KY16 9JP, Scotland, UK

[⌂] School of Biology, University of St Andrews, St Andrews, Fife, KY16 9TF, Scotland, UK

* Corresponding author:

Konstanze Krueger,

Konstanze.Krueger@hfwu.de

tel: 0049 7022 201

Data per horses

	sorce	sorce num	video / quest	video_quest_num	sID	nr reported behaviours	type of other behaviours	how often was the behaviour shown	behaviour frequency	social learning (spreading to other horses)	sex	sex num	age	breed	breed-type	breed type num	length of domestication	purpose of domestication	single group stabling	single group stabling	restricted unrestricted pasture	restricted unrestricted pasture	restricted unrestricted contact with horses	restricted unrestricted contact with horses	restricted unrestricted roughage	restricted unrestricted roughage	link
1																											
2	gen.Engl.	1	quest	1	1958,0	1	0	dayly	5		g	2	7,0	Quarter Horse	Warmblood	1	350	3	2	group	2	unrestricted	2	unrestricted	2	unrestricted	
3	gen.Engl.	1	quest	1	1965,0	5	1. unty knot 2. asking	2-10 times	2	0	s	3	1,0	Warmblood	Warmblood	1	350	3	2	group	2	unrestricted	2	unrestricted	2	unrestricted	
4	gen.Engl.	1	quest	1	1978,0	1	0	dayly	5		m	1	18,0	Connemara	Pony	2	100	5	2	group	2	unrestricted	2	unrestricted	2	unrestricted	
5	gen.Engl.	1	quest	1	1980,0	4	knot untying, asking	more than 20	4	0	s	3	0,5	Lusitano	Warmblood	1	350	3	1	single	2	unrestricted	2	unrestricted	2	unrestricted	
6	gen.Engl.	1	quest	1	1981,0	1	Esperia ponys turn	2-10 times	2					Esperia Pony	Warmblood	1	350	3	2	group	1	restricted	1	restricted	1	restricted	
7	gen.Engl.	1	quest	1	2001,0	1	0	2-10 times	2		g	2	4,0	Appaloosa	Warmblood	1	350	3	2	group	2	unrestricted	2	unrestricted	1	restricted	
8	gen.Engl.	1	quest	1	2003,0	2	opening feed tons	dayly	5	1	g	2	8,0	Haflinger	Warmblood	1	350	3	1	single	2	unrestricted	2	unrestricted	2	unrestricted	
9	gen.Engl.	1	quest	1	2004,0	3	0	11-20 times	3	0	g	2	6,0	Shetland pony	Pony	2	100	5	1	single	2	unrestricted	2	unrestricted	1	restricted	
10	gen.Engl.	1	quest	1	2005,0	1	0	dayly	5	0	g	2	10,0	KWPN	Warmblood	1	350	3	1	single	1	restricted	1	restricted	1	restricted	
11	gen.Engl.	1	quest	1	2006,0	1	0	dayly	5	0	g	2	8,0	Swedish Warmblood	Warmblood	1	350	3	1	single	2	unrestricted	2	unrestricted	1	restricted	
12	gen.Engl.	1	quest	1	2008,0	1	0	more than 20	4	0	m	1	6,0	NA	NA				1	single	1	restricted	1	restricted	1	restricted	
13	gen.Engl.	1	quest	1	2010,0	3	1. untie himself, 2. lift	dayly	5	0	g	2	8,0	Welsh D	Warmblood	1	350	3	1	single	2	unrestricted	2	unrestricted	1	restricted	
14	gen.Engl.	1	quest	1	2011,0	1	0	more than 20	4	0	m	1	9,0	Friesian horse	Warmblood	1	350	3	1	single	2	unrestricted	2	unrestricted	1	restricted	
15	gen.Engl.	1	quest	1	2012,0	1	0	dayly	5	0	g	2	13,0	KWPN	Warmblood	1	350	3	1	single	2	unrestricted	2	unrestricted	2	unrestricted	
16	gen.Engl.	1	quest	1	2013,0	1	0	2-10 times	2	0	m	1	4,0	Paint horse	Warmblood	1	350	3	2	group	2	unrestricted	2	unrestricted	2	unrestricted	
17	gen.Engl.	1	quest	1	2014,0	1	0	more than 20	4	0	m	1	6,0	Tinker horse	Draught horse	4	500	4	2	group	2	unrestricted	2	unrestricted	2	unrestricted	
18	gen.Engl.	1	quest	1	2016,0	1	0	2-10 times	2	0	g	2	9,0	KWPN	Warmblood	1	350	3	1	single	2	unrestricted	2	unrestricted	2	unrestricted	
19	gen.Engl.	1	quest	1	2017,0	2	tapping with hindleg against things in her way, looking at owner to remove it	dayly	5		m	1	8,0	Dutch Warmblood	Warmblood	1	350	3	1	single	2	unrestricted	2	unrestricted	2	unrestricted	
20	gen.Engl.	1	quest	1	2018,0	4	unty rope, take off	dayly	5	0	g	2	14,0	KWPN	Warmblood	1	350	3	1	single	1	restricted	1	restricted	2	unrestricted	
21	gen.Engl.	1	quest	1	2019,0	2	0	11-20 times	3	0	m	1	10,0	NA	Warmblood	1	350	3	2	group	2	unrestricted	2	unrestricted	1	restricted	
22	gen.Engl.	1	quest	1	2020,0	1		dayly	5		m	1	15,0	Haflinger	Warmblood	1	350	3	1	single	2	unrestricted	2	unrestricted	1	restricted	
23	gen.Engl.	1	quest	1	2021,0	3	pulling sticks of	2-10 times	2	0	m	1	6,0	Appaloosa	Warmblood	1	350	3	2	group	2	unrestricted	2	unrestricted	2	unrestricted	
24	gen.Engl.	1	quest	1	2022,0	2	open ropes	more than 20	4		g	2	25,0	Connemara	Pony	2	100	5	2	group	2	unrestricted	2	unrestricted	2	unrestricted	
25	gen.Engl.	1	quest	1	2030,0	1		2-10 times	2		g	2	14,0	Arabian	Arabian horse	3	4000	1	2	group	2	unrestricted	2	unrestricted	2	unrestricted	
26	gen.Engl.	1	quest	1	2031,0	12	1. picking up whip an	more than 20	4	0	g	2	15,0	Missouri Fox	Warmblood	1	350	3	1	single	2	unrestricted	1	restricted	1	restricted	
27	gen.Engl.	1	quest	1	2032,0	5	opening box	2-10 times	2		g	2	8,0	Iceland horse	Pony	2	100	5	1	single	2	unrestricted	1	restricted	1	restricted	
28	gen.Engl.	1	quest	1	2033,0	4	take bridl off, after an	2-10 times	2	0	g	2	9,0	Friesian horse	Warmblood	1	350	3	1	single	1	restricted	1	restricted	1	restricted	
29	gen.Engl.	1	quest	1	2034,0	5	untying other,	more than 20	4		g	2	3,0	Palomino	Warmblood	1	350	3	2	group	2	unrestricted	2	unrestricted	2	unrestricted	
30	gen.Engl.	1	quest	1	2036,0	1	0	more than 20	4	0	m	1	12,0	Finhorse	Warmblood	1	350	3	2	group	2	unrestricted	2	unrestricted	1	restricted	
31	gen.Engl.	1	quest	1	2038,0	1		more than 20	4		m	1	19,0	Belgian horse	Draught horse	4	500	4	2	group	2	unrestricted	2	unrestricted	1	restricted	
32	gen.Engl.	1	quest	1	2039,0	2	0	more than 20	4	0	g	2	7,0	Quarter horse	Warmblood	1	350	3	1	single	2	unrestricted	2	unrestricted	2	unrestricted	
33	gen.Engl.	1	quest	1	2040,0	2	0	more than 20	4	0	m	1	7,0	Arabian	Arabian horse	3	4000	1	1	single	1	restricted	1	restricted	1	restricted	
34	gen.Engl.	1	quest	1	2042,0	1		2-10 times	2		g	2	3,0	Arabian	Arabian horse	3	4000	1	1	single	1	restricted	1	restricted	1	restricted	
35	gen.Engl.	1	quest	1	2044,0	5	1. unty knotted ropes	dayly	5	0	g	2	2,0	Quarter horse	Warmblood	1	350	3	2	group	2	unrestricted	2	unrestricted	1	restricted	
36	gen.Engl.	1	quest	1	2046,0	3	open tack room door,	more than 20	4		m	1	13,0	Missouri Fox	Warmblood	1	350	3	2	group	2	unrestricted	2	unrestricted	1	restricted	
37	gen.Engl.	1	quest	1	2051,0	1		more than 20	4		g	2	21,0	Irish hunter	Warmblood	1	350	3	1	single	2	unrestricted	1	restricted	1	restricted	
38	gen.Engl.	1	quest	1	2062,0	2	0	more than 20	4	0	g	2	10,0	Anglo-Arab	Arabian horse	3	4000	1	1	single	2	unrestricted	2	unrestricted	2	unrestricted	
39	gen.Engl.	1	quest	1	2063,0	1	Opens and shuts the	more than 20	4	0	g	2	5,0	Anglo-Arab	Arabian horse	3	4000	1	1	single	2	unrestricted	2	unrestricted	2	unrestricted	

1	sorce	sorce num	video / quest	video_quest_num	sID	nr reported behaviours	type of other behaviours	how often was the behaviour shown	behaviour frequency	social learning (spreading to other horses)	sex	sex num	age	breed	breed-type	breed type num	length of domestication	purpose of domestication	single group stabling	single group stabling	restricted unrestricted pasture	restricted unrestricted pasture	restricted unrestricted contact with horses	restricted unrestricted contact with horses	restricted unrestricted roughage	restricted unrestricted roughage	link
39	gen.Engl.	1	quest	1	2063,0	1	Opens and shuts the	more than 20	4	0	g	2	5,0	Anglo-Arab	Arabian horse	3	4000	1	1	single	2	unrestricted	2	unrestricted	2	unrestricted	
40	gen.Engl.	1	quest	1	2064,0	4	1. opening bins 2. throws a ball at people when they pass	more than 20	4	1	g	2	12,0	Morgan horse	Warmblood	1	350	3	1	single	2	unrestricted	2	unrestricted	2	unrestricted	
41	gen.Engl.	1	quest	1	2066,0	1		11-20 times	3		g	2	20,0	NA	NA				1	single	1	restricted	1	restricted	1	restricted	
42	gen.Germ.	2	quest	1	1947,0	1	bucking and kicking	dayly	5	1	m	1	3,0	NA	Warmblood	1	350	3	2	group	2	unrestricted	2	unrestricted	2	unrestricted	
43	gen.Engl.	1	quest	1	1952,0	2	opening freezer	2-10 times	2		m	1	2,0	Friesian horse	Warmblood	1	350	3	2	group	2	unrestricted	2	unrestricted	2	unrestricted	
44	gen.Engl.	1	quest	1	1957,0	1		dayly	5		s	3	12,0	Selle Francais	Warmblood	1	350	3	2	group	2	unrestricted	2	unrestricted	2	unrestricted	
45	gen.Engl.	1	quest	1	1960,0	3	opening knots ,	2-10 times	2		g	2	7,0	Paint horse	Warmblood	1	350	3	1	single	2	unrestricted	2	unrestricted	1	restricted	
46	gen.Engl.	1	quest	1	1961,0	5	Pulling out hay of the	dayly	5		g	2	9,0	Hispano Arabe	Arabian horse	3	4000	1	2	group	2	unrestricted	2	unrestricted	1	restricted	
47	gen.Germ.	2	quest	1	1963,0	1	NA	dayly	5	0	m	1	14,0	German Riding	Pony	2	100	5	1	single	1	restricted	1	restricted	2	unrestricted	
48	gen.Engl.	1	quest	1	1964,0	2	opening safety-knot	dayly	5		g	2	10,0	Trotter Mix	Thoroughbre	5	250	2	2	group	2	unrestricted	2	unrestricted	1	restricted	
49	gen.Engl.	1	quest	1	1969,0	1	NA	dayly	5		m	1	31,0	Mini Shetland	Pony	2	100	5	1	single	2	unrestricted	2	unrestricted	1	restricted	
50	gen.Germ.	2	quest	1	1969,1	1	NA	dayly	5	0	m	1	13,0	NA	Warmblood	1	350	3	1	single	2	unrestricted	2	unrestricted	1	restricted	
51	gen.Engl.	1	quest	1	1970,0	2	if the horse thinks he has worked enough he goes to the middle of the riding arena and shows a spin?	dayly	5		s	3	4,0	PRE	Warmblood	1	350	3	1	single	2	unrestricted	2	unrestricted	1	restricted	
52	gen.Germ.	2	quest	1	1971,0	2	waters plants	more than 20	4	0	g	2	6,0	Pinto	Warmblood	1	350	3	1	single	2	unrestricted	1	restricted	1	restricted	
53	gen.Engl.	1	quest	1	1972,0	2		more than 20	4		g	2	13,0	Fjordhorse	Warmblood	1	350	3	1	single	2	unrestricted	1	restricted	2	unrestricted	
54	gen.Germ.	2	quest	1	1972,1	2	open feed container	2-10 times	2	0	s	3	30,0	NA	Pony	2	100	5	1	single	2	unrestricted	1	restricted	2	unrestricted	
55	gen.Germ.	2	quest	1	1977,0	2	unty knots	dayly	5	0	g	2	3,0	Paint horse	Warmblood	1	350	3	2	group	2	unrestricted	2	unrestricted	1	restricted	
56	gen.Engl.	1	quest	1	1982,0	1		11-20 times	3		s	3	24,0	NA	Pony	2	100	5	2	group	2	unrestricted	2	unrestricted	2	unrestricted	
57	gen.Germ.	2	quest	1	1982,1	1	0	11-20 times	3	0	s	3	24,0	NA	Pony	2	100	5	2	group	2	unrestricted	2	unrestricted	2	unrestricted	
58	gen.Engl.	1	quest	1	1983,0	1		more than 20	4		g	2	14,0	Württemberger	Warmblood	1	350	3	1	single	2	unrestricted	2	unrestricted	1	restricted	
59	gen.Germ.	2	quest	1	1984,0	4	1. unty knots 2.	NA		0	g	2	9,0	Arab mix	Arabian horse	3	4000	1	2	group	2	unrestricted	2	unrestricted	1	restricted	
60	gen.Engl.	1	quest	1	1961,1	1		NA			m	1	14,0	Haflinger	Warmblood	1	350	3	2	group	2	unrestricted	2	unrestricted	1	restricted	
61	gen.Germ.	2	quest	1	1986,0	3	1.unty knots 3. raking	dayly	5	0	g	2	8,0	Pony mix	Pony	2	100	5	1	single	2	unrestricted	2	unrestricted	1	restricted	
62	gen.Germ.	2	quest	1	1988,0	1	1. open fence	NA			g	2	12,0	Selle Francais	Warmblood	1	350	3									
63	gen.Engl.	1	quest	1	1989,0	1		NA			g	2	18,0	Freiberger	Warmblood	1	350	3	2	group							
64	gen.Engl.	1	quest	1	1990,0	1		dayly	5		g	2		Friesian horse	Warmblood	1	350	3	2	group							
65	gen.Germ.	2	quest	1	1990,1	4	open feed box	dayly	5	0	m	1	3,0	NA	Warmblood	1	350	3	1	single	1	restricted	2	unrestricted	1	restricted	
66	gen.Engl.	1	quest	1	1993,0	1		NA			g	2	3,0	Friesian horse	Warmblood	1	350	3	2	group							
67	gen.Engl.	1	quest	1	1994,0	1		dayly	5		g	2	21,0	Camargue	Warmblood	1	350	3	2	group	2	unrestricted			1	restricted	
68	gen.Engl.	1	video	2	1998,0	1		NA			NA			NA	Warmblood	1	350	3									
69	gen.Engl.	1	video	2	1999,0	1		NA			g	2		NA	Warmblood	1	350	3									
70	gen.Germ.	2	quest	1	2000,0	2	1.takes down electric	more than 20	4	0	g	2	19,0	NA	Warmblood	1	350	3	1	single	2	unrestricted	2	unrestricted			
71	gen.Engl.	1	quest	1	2023,0	2		more than 20	4		g	2	19,0	Oldenburg	Warmblood	1	350	3	1	single	2	unrestricted	2	unrestricted	1	restricted	
72	gen.Engl.	1	video	2	2037,0	1		NA			NA		0,5	NA	Warmblood	1	350	3	1	single							
73	gen.Engl.	1	video	2	2056,0	1		NA			g	2		NA	Warmblood	1	350	3									
74	gen.Engl.	1	video	2	2057,0	1		NA			g	2		NA	Warmblood	1	350	3									
75	gen.Engl.	1	quest	1	2061,0	1		more than 20	4		g	2	3,0	Iceland horse	Pony	2	100	5	2	group	2	unrestricted	1	restricted			
[illegible]	[illegible]	[illegible]	[illegible]	[illegible]	[illegible]	[illegible]	[illegible]	[illegible]	[illegible]	[illegible]	[illegible]	[illegible]	[illegible]	[illegible]	[illegible]	[illegible]	[illegible]	[illegible]	[illegible]	[illegible]	[illegible]	[illegible]	[illegible]	[illegible]	[illegible]	[illegible]	[illegible]

1	sorce	sorce num	video/ quest	video_quest_num	sID	nr reported behaviours	type of other behaviours	how often was the behaviour shown	behaviour frequency	social learning (spreading to other horses)	sex	sex num	age	breed	breed-type	breed type num	length of domestication	purpose of domestication	single group stabling	single group stabling	restricted unrestricted pasture	restricted unrestricted pasture	restricted unrestricted contact with horses	restricted unrestricted contact with horses	restricted unrestricted roughage	restricted unrestricted roughage	link
77	gen.Germ.	2	video	2	2071,0	1	NA	NA			NA			NA	Warmblood	1	350	3	1	single							Ein schlaues Pferd - YouTube [720p].mp4
78	gen.Engl.	1	video	2	2074,0	1		NA			NA			NA	Warmblood	1	350	3									
79	gen.Germ.	2	video	2	2075,0	1	NA	NA			NA			NA	Warmblood	1	350	3									https://www.youtube.com/watch?v=YVMej_ApKMw
80	gen.Engl.	1	quest	1	2076,0	1		dayly	5		g	2	10,0	Paso Peruano	Warmblood	1	350	3	2	group	2	unrestricted	2	unrestricted	2	unrestricted	
81	gen.Engl.	1	quest	1	2077,0	1		dayly	5		g	2	6,0	Swedish	Draught horse	4	500	4	2	group	2	unrestricted	2	unrestricted	1	restricted	
82	gen.Engl.	1	video	2	2079,0	1	NA	NA			g	2		NA	Warmblood	1	350	3	1	single							http://youtu.be/DuleXhBpZKs
83	gen.Germ.	2	video	2	2080,0	1	NA	NA			m	1		NA	Warmblood	1	350	3	1	single							http://youtu.be/LS69RAiOOus
84	gen.Engl.	1	video	2	2081,0	1	NA	NA			m	1		NA	Warmblood	1	350	3	1	single							http://youtu.be/_UIByc0rIyA
85	gen.Engl.	1	video	2	2082,0	1		NA			g	2		NA	Warmblood	1	350	3									
86	gen.Engl.	1	video	2	2083,0	1		NA			m	1		Arabian	Arabian horse	3	4000	1									My horse unlocks her door!_0.mp4
87	gen.Engl.	1	video	2	2084,0	1	NA	NA			m	1		NA	Warmblood	1	350	3	1	single							http://youtu.be/a1IAVrKHtIA
88	gen.Engl.	1	video	2	2085,0	1	NA	NA			NA			NA	Warmblood	1	350	3	1	single							http://youtu.be/1XWFjUjNq3E
89	gen.Engl.	1	video	2	2086,0	1	NA	NA			m	1		NA	Warmblood	1	350	3									http://youtu.be/Ntydqyo6D8o
90	gen.Engl.	1	video	2	2087,0	1	NA	NA			g	2		NA	Warmblood	1	350	3									http://youtu.be/awuHaxptcgc
91	gen.Engl.	1	video	2	2088,0	1	NA	NA			g	2		NA	Warmblood	1	350	3									http://youtu.be/8uJ05KN4rhg
92	gen.Engl.	1	video	2	2089,0	1		NA			NA			NA	Warmblood	1	350	3									
93	gen.Engl.	1	video	2	2090,0	1	NA	NA			m	1		NA	Warmblood	1	350	3									http://youtu.be/BLejxIfZkkA
94	gen.Engl.	1	video	2	2091,0	1	NA	NA			g	2		NA	Warmblood	1	350	3									http://youtu.be/EJDoCMj-WIQ
95	gen.Engl.	1	video	2	2093,0	1	NA	NA			NA			NA	Pony	2	100	5									http://youtu.be/7Zf4IbMe7VM
96	gen.Engl.	1	video	2	2094,0	1		NA			NA			NA	Warmblood	1	350	3									
97	gen.Engl.	1	video	2	2095,0	1		NA			NA			NA	Warmblood	1	350	3									http://youtu.be/OYW5oUQGRLk
98	gen.Engl.	1	video	2	2096,0	2	NA	NA			NA			NA	NA												http://youtu.be/90Xqn0uUnaY
99	gen.Engl.	1	video	2	2097,0	4		NA			m	1		NA	Warmblood	1	350	3									
100	gen.Engl.	1	video	2	2101,0	1		NA			m	1		NA	Warmblood	1	350	3									
101	gen.Engl.	1	video	2	2103,0	1	NA	NA			NA			NA	Arabian horse	3	4000	1									http://youtu.be/vFc9bQ0Vs3Q
102	gen.Engl.	1	video	2	2104,0	1	NA	NA			g	2		NA	Warmblood	1	350	3									http://youtu.be/UnuuukezIUw
103	gen.Engl.	1	video	2	2106,0	1	NA	NA			g	2		NA	Warmblood	1	350	3									http://youtu.be/0SDpaxiIWfI
104	gen.Engl.	1	video	2	2107,0	1	NA	NA			g	2		NA	Warmblood	1	350	3									http://youtu.be/nY2owCKXAr4
105	gen.Engl.	1	video	2	2108,0	1		11-20 times	3		s	3		Iceland horse	Pony	2	100	5									
106	gen.Engl.	1	video	2	2109,0	1	NA	NA			g	2		NA	Warmblood	1	350	3									http://youtu.be/MTg4M5D3fH4
107	gen.Engl.	1	video	2	2110,0	1		NA			NA			Miniature horse	Pony	2	100	5									
108	gen.Engl.	1	video	2	2111,0	1		NA			NA			NA	Warmblood	1	350	3									
109	gen.Engl.	1	video	2	2112,0	1		NA			g	2		NA	Warmblood	1	350	3									
110	gen.Engl.	1	video	2	2113,0	1	NA	NA			NA			NA	Warmblood	1	350	3									http://youtu.be/M-LvDjityTM
111	gen.Engl.	1	video	2	2114,0	1		NA			m	1		NA	Warmblood	1	350	3									
112	gen.Engl.	1	video	2	2115,0	1		NA			g	2		NA	Warmblood	1	350	3									
113	gen.Engl.	1	video	2	2116,0	1		NA			NA			NA	NA												
114	gen.Engl.	1	video	2	2118,0	1		NA			g	2		NA	Mule	6	3000	4									
115	gen.Engl.	1	video	2	2119,0	1		NA			NA		0,5	NA	Warmblood	1	350	3									
116	gen.Engl.	1	video	2	2120,0	1		NA			NA			NA	Warmblood	1	350	3									
117	gen.Engl.	1	video	2	2121,0	1	NA	NA			g	2		NA	Warmblood	1	350	3									http://youtu.be/T7wi6Bt8O3g
118	gen.Engl.	1	video	2	2122,0	1		NA			g	2	12,0	NA	Warmblood	1	350	3									
119	gen.Engl.	1	video	2	2123,0	1	0	more than 20 t	4	2	g	2		NA	NA				1	single							
120	gen.Engl.	1	quest	1	2125,0	1		2-10 times	2		g	2	7,0	Iceland horse	Pony	2	100	5	2	group	2	unrestricted	2	unrestricted	2	unrestricted	

1	sorce	sorce num	video / quest	video_quest_num	sID	nr reported behaviours	type of other behaviours	how often was the behaviour shown	behaviour frequency	social learning (spreading to other horses)	sex	sex num	age	breed	breed-type	breed type num	length of domestication	purpose of domestication	single group stabling	single group stabling	restricted unrestricted pasture	restricted unrestricted pasture	restricted unrestricted contact with horses	restricted unrestricted contact with horses	restricted unrestricted roughage	restricted unrestricted roughage	link
121	gen.Engl.	1	quest	1	2128,0	1		more than 20	4		m	1	5,0	Iceland horse	Pony	2	100	5	2	group	2	unrestricted	2	unrestricted	1	restricted	
122	gen.Engl.	1	quest	1	2078,0	3	0	dayly	5	1	g	2	2,0	Appaloosa	Warmblood	1	350	3	2	group	2	unrestricted	2	unrestricted	2	unrestricted	
123	gen.Engl.	1	quest	1	2117,0	1		11-20 times	3	0	s	3	5,0	Iceland horse	Pony	2	100	5	1	single	2	unrestricted	1	restricted	1	restricted	
124	gen.Engl.	1	quest	1	2124,0	7	1. can untie knots, 2.	2-10 times	2		s	3	5,0	Iceland horse	Pony	2	100	5	1	single	2	unrestricted	1	restricted	1	restricted	
125	gen.Engl.	1	quest	1	2126,0	2	unty lead ropes	more than 20 (	4		g	2	4,0	Paint horse	Warmblood	1	350	3	1	single	2	unrestricted	2	unrestricted	2	unrestricted	
126	gen.Engl.	1	quest	1	2127,0	1	0	dayly	5	0	g	2	9,0	Quarter horse	Warmblood	1	350	3	2	group	2	unrestricted	2	unrestricted	1	restricted	
127	gen.Germ.	2	video	2	2130,0	1		NA			m	1		Arab mix	Arabian horse	3	4000	1									
128	gen.Germ.	2	quest	1	2131,0	1	NA	NA			m	1		NA	Warmblood	1	350	3									http://youtu.be/osbxMbHUNe8
129	gen.Germ.	2	video	2	2132,0	1		NA			g	2		NA	Warmblood	1	350	3									
130	gen.Germ.	2	video	2	2133,0	1	NA	NA			g	2		NA	Arabian horse	3	4000	1									http://youtu.be/m2B3_qwwglM
131	gen.Germ.	2	video	2	2134,0	1	NA	NA			NA			NA	NA												http://youtu.be/jyWR6F-EaeY
132	gen.Germ.	2	video	2	2135,0	1	NA	NA			g	2		NA	Warmblood	1	350	3									http://youtu.be/AzLToOWyXyk
133	gen.Germ.	2	video	2	2136,0	1	NA	NA			m	1		NA	Warmblood	1	350	3									http://youtu.be/1yLYMfwFqis
134	gen.Germ.	2	video	2	2137,0	1	NA	NA			g	2		NA	Pony	2	100	5									http://youtu.be/TipjTiYOgE4
135	gen.Germ.	2	video	2	2138,0	1	NA	NA			NA			NA	NA												http://youtu.be/b0BUqwqLI3wNAt=31s
136	gen.Germ.	2	video	2	2140,0	1		NA			m	1		NA	Pony	2	100	5									
137	gen.Germ.	2	video	2	2141,0	1		NA			NA			NA	Warmblood	1	350	3									
138	gen.Engl.	1	quest	1	2146,0	1	0	more than 20 (	4	0	m	1	1,0	NA	Warmblood	1	350	3	1	single	2	unrestricted	2	unrestricted	1	restricted	
139	gen.Engl.	1	quest	1	2147,0	3	taking of head-gears	dayly	5		g	2	7,0	Paint horse	Warmblood	1	350	3	1	single	2	unrestricted	2	unrestricted	1	restricted	
140	gen.Engl.	1	quest	1	2148,0	1	0	11-20 times	3	0	s	3	3,0	Rocky Mountain	Warmblood	1	350	3	2	group	2	unrestricted	2	unrestricted	2	unrestricted	
141	gen.Engl.	1	quest	1	2150,0	3	unty knots	more than 20 (	4	0	g	2	26,0	Selle Francais	Warmblood	1	350	3	2	group	2	unrestricted	2	unrestricted	2	unrestricted	
142	gen.Engl.	1	quest	1	2152,0	4	1.unty knots,	more than 20 (	4	0	g	2	15,0	Danish	Warmblood	1	350	3	1	single	2	unrestricted	2	unrestricted	2	unrestricted	
143	gen.Germ.	2	quest	1	2151,0	1		dayly	5		g	2	9,0	Hannoveraner	Warmblood	1	350	3	2	group	2	unrestricted	2	unrestricted	2	unrestricted	
144	gen.Germ.	2	quest	1	2155,0	1		2-10 times	2		m	1		Hannoveraner	Warmblood	1	350	3	1	single	2	unrestricted	2	unrestricted	1	restricted	
145	gen.Engl.	1	quest	1	2153,0	2	0	11-20 times	3	0	m	1	17,0	KWPN	Warmblood	1	350	3	1	single	2	unrestricted	2	unrestricted	1	restricted	
146	gen.Germ.	2	quest	1	2156,0	2	bucket so that food falls to one side of the bucket	more than 20 times	4		g	2	6,0	Scottish Highland Pony	Warmblood	1	350	3	2	group	2	unrestricted	2	unrestricted	1	restricted	
147	gen.Germ.	2	quest	1	2157,0	2		NA			g	2	4,0	Hannoveraner	Warmblood	1	350	3	2	group	2	unrestricted	2	unrestricted	2	unrestricted	
148	gen.Germ.	2	quest	1	2158,0	1	0	dayly	5	0	s	3	10,0	NA	NA				1	single	2	unrestricted	2	unrestricted	1	restricted	
149	gen.Germ.	2	quest	1	2159,0	1		11-20 times	3		g	2	8,0	Trakehner	Warmblood	1	350	3	2	group	2	unrestricted	2	unrestricted	2	unrestricted	
150	gen.Germ.	2	quest	1	2161,0	1		11-20 times	3		g	2	5,0	Arab mix	Arabian horse	3	4000	1	2	group	2	unrestricted	2	unrestricted	2	unrestricted	
151	gen.Germ.	2	quest	1	2164,0	1		dayly	5		g	2	8,0	Friesian horse	Warmblood	1	350	3	2	group	2	unrestricted	2	unrestricted	1	restricted	
152	gen.Germ.	2	quest	1	2165,0	1		more than 20 times	4		g	2	9,0	Hannoveraner	Warmblood	1	350	3	2	group	2	unrestricted	2	unrestricted	1	restricted	
153	gen.Germ.	2	quest	1	2166,0	1		dayly	5		m	1	10,0	NA	NA				1	single	1	restricted	1	restricted	1	restricted	
154	gen.Engl.	1	quest	1	2162,0	1		2-10 times	2		g	2	8,0	Trakehner Arabian mix	Warmblood	1	350	3	2	group	2	unrestricted	2	unrestricted	2	unrestricted	
155	gen.Engl.	1	quest	1	2163,0	3	1. unty knots 2. take down hlater	dayly	5	1	s	3	15,0	PRE	Warmblood	1	350	3	2	group	2	unrestricted	2	unrestricted	2	unrestricted	
156	gen.Engl.	1	quest	1	2168,0	5	1. unty knots	NA		0	g	2	10,0	Arabian	Arabian horse	3	4000	1	2	group	2	unrestricted	2	unrestricted	2	unrestricted	
157	gen.Germ.	2	quest	1	2169,0	1	0	NA		0	g	2	7,0	NA	NA				2	group	2	unrestricted	2	unrestricted	2	unrestricted	
158	gen.Engl.	1	quest	1	2173,0	1	0	2-10 times	2	0	g	2	7,0	Iceland horse	Pony	2	100	5	2	group	2	unrestricted	2	unrestricted	2	unrestricted	
159	gen.Germ.	2	quest	1	2174,0	4	remove, take off, halter	2-10 times	2	0	g	2	16,0	Fjordhorse	Warmblood	1	350	3	2	group	2	unrestricted	2	unrestricted	2	unrestricted	
160	gen.Germ.	2	quest	1	2175,0	3	take down rags	dayly	5	0	g	2	7,0	Württemberger	Warmblood	1	350	3	2	group	2	unrestricted	2	unrestricted	2	unrestricted	

1	sorce	sorce num	video / quest	video_quest_num	sID	nr reported behaviours	type of other behaviours	how often was the behaviour shown	behaviour frequency	social learning (spreading to other horses)	sex	sex num	age	breed	breed-type	breed type num	length of domestication	purpose of domestication	single group stabling	single group stabling	restricted unrestricted pasture	restricted unrestricted pasture	restricted unrestricted contact with horses	restricted unrestricted contact with horses	restricted unrestricted roughage	restricted unrestricted roughage	link
162	gen.Germ.	2	quest	1	2191,0	1		11-20 times	3		g	2	5,0	Arab mix	Arabian horse	3	4000	1	1	single	1	restricted	2	unrestricted	1	restricted	
163	gen.Germ.	2	quest	1	2193,0	1		11-20 times	3		g	2	4,0	Haflinger	Warmblood	1	350	3	2	group	2	unrestricted	2	unrestricted	2	unrestricted	
164	gen.Germ.	2	quest	1	2194,0	1		dayly	5		g	2	4,0	Haflinger	Warmblood	1	350	3	2	group	2	unrestricted	2	unrestricted	2	unrestricted	
165	gen.Germ.	2	quest	1	2217,0	1	NA	dayly	5	0	g	2	5,0	Arab mix	Arabian horse	3	4000	1	2	group	2	unrestricted	2	unrestricted	2	unrestricted	
166	gen.Germ.	2	quest	1	2225,0	1		more than 20 times	4		m	1	10,0	Westfahle	Warmblood	1	350	3	1	single	1	restricted	1	restricted	1	restricted	
167	gen.Germ.	2	quest	1	2231,0	2		NA			m	1	9,0	Lewitzer	Draught horse	4	500	4	1	single	2	unrestricted	2	unrestricted	2	unrestricted	
168	gen.Germ.	2	quest	1	2233,0	2	NA	more than 20 (	4	1	m	1	28,0	Haflinger	Warmblood	1	350	3	2	group	2	unrestricted	2	unrestricted	2	unrestricted	
169	gen.Germ.	2	quest	1	2238,0	3	open gates, open feed-box, unty knots,	more than 20 (	4	0	m	1	6,0	Freiberger	Warmblood	1	350	3	2	group	2	unrestricted	2	unrestricted	2	unrestricted	
170	gen.Germ.	2	quest	1	2251,0	2		2-10 times	2		g	2	7,0	Haflinger	Warmblood	1	350	3	1	single	2	unrestricted	2	unrestricted	2	unrestricted	
171	gen.Germ.	2	quest	1	2282,0	1		2-10 times	2		g	2	19,0	Paint horse	Warmblood	1	350	3	1	single	2	unrestricted	2	unrestricted	2	unrestricted	
172	gen.Germ.	2	quest	1	2297,0	6	water tap, turn light on, take halter down, unty knots, open electric fences (at handle)	2-10 times	2	1	m	1	5,0	Hannoveraner	Warmblood	1	350	3	1	single	2	unrestricted	2	unrestricted	1	restricted	
173	gen.Germ.	2	quest	1	2299,0	1		more than 20 times	4		m	1	6,0	Westfahle	Warmblood	1	350	3	1	single	2	unrestricted	2	unrestricted	2	unrestricted	
174	gen.Germ.	2	quest	1	2327,0	2	take off muzzel,approach other horses for taking muzzle off	dayly	5		g	2	9,0	Arab mix	Arabian horse	3	4000	1	2	group	2	unrestricted	2	unrestricted	1	restricted	
175	gen.Germ.	2	quest	1	2377,0	1		dayly	5		m	1	26,0	Oldenburg	Warmblood	1	350	3	1	single	2	unrestricted	2	unrestricted	1	restricted	
176	gen.Germ.	2	quest	1	2404,0	1	NA	dayly	5	0	g	2	10,0	Rheinisches Warmblood	Warmblood	1	350	3	2	group	2	unrestricted	2	unrestricted	2	unrestricted	
177	gen.Germ.	2	quest	1	2405,0	1	NA	2-10 times	2	0	g	2	12,0	Oldenburg	Warmblood	1	350	3	2	group	2	unrestricted	2	unrestricted	2	unrestricted	
178	gen.Germ.	2	quest	1	2441,0	1		dayly	5		g	2	3,0	Arab mix	Arabian horse	3	4000	1	2	group	2	unrestricted	2	unrestricted	2	unrestricted	
179	gen.Germ.	2	quest	1	2251,0	2	open water tap, open door of trailor from the inside	2-10 times	2	0	g	2	7,0	NA	Warmblood	1	350	3	1	single	1	restricted	1	restricted	1	restricted	
180	gen.Germ.	2	quest	1	2452,0	2		NA			g	2	8,0	hungarian warmblood	Warmblood	1	350	3	2	group	2	unrestricted	2	unrestricted	2	unrestricted	
181	gen.Germ.	2	quest	1	2470,0	1	NA	more than 20 (	4	1	g	2	10,0	Connemara	Pony	2	100	5	2	group	2	unrestricted	2	unrestricted	2	unrestricted	
182	gen.Engl.	1	quest	1	2265,0	4	crouch underneath fences, open latches, open slide door	2-10 times	2	0	m	1	5,0	Quarter horse	Warmblood	1	350	3	2	group	2	unrestricted	2	unrestricted	2	unrestricted	
183	gen.Engl.	1	quest	1	2272,0	1		NA			g	2	10,0	Tennessee Walking Horse	Warmblood	1	350	3	1	single	2	unrestricted	2	unrestricted	1	restricted	
184	gen.Engl.	1	quest	1	2275,0	1		more than 20 times	4		m	1	10,0	Belgium / Tennessee Walking cross	Warmblood	1	350	3	2	group	2	unrestricted	2	unrestricted	2	unrestricted	
185	gen.Engl.	1	quest	1	2279,0	1		2-10 times	2		g	2	18,0	Paint horse	Warmblood	1	350	3	2	group	2	unrestricted	2	unrestricted	2	unrestricted	
186	gen.Engl.	1	quest	1	2342,0	1		11-20 times	3		g	2	9,0	Pony	Pony	2	100	5	2	group	2	unrestricted	2	unrestricted	1	restricted	
187	gen.Engl.	1	quest	1	2315,0	1		more than 20 times	4		g	2	10,0	Tennessee Walking horse	Warmblood	1	350	3	1	single	2	unrestricted	2	unrestricted	1	restricted	watch?v=gmJ9wbpPSsQ
188	gen.Engl.	1	quest	1	2428,0	1		2-10 times	2		g	2	4,0	Miniture horse	Pony	2	100	5	2	group	2	unrestricted	2	unrestricted	2	unrestricted	
[illegible]	[illegible]	[illegible]	[illegible]	[illegible]	[illegible]	[illegible]	[illegible]	[illegible]	[illegible]	[illegible]	[illegible]	[illegible]	[illegible]	[illegible]	[illegible]	[illegible]	[illegible]	[illegible]	[illegible]	[illegible]	[illegible]	[illegible]	[illegible]	[illegible]	[illegible]	[illegible]	

1	sorce	sorce num	video / quest	video_quest_num	sID	nr reported behaviours	type of other behaviours	how often was the behaviour shown	behaviour frequency	social learning (spreading to other horses)	sex	sex num	age	breed	breed-type	breed type num	length of domestication	purpose of domestication	single group stabling	single group stabling	restricted unrestricted pasture	restricted unrestricted pasture	restricted unrestricted contact with horses	restricted unrestricted contact with horses	restricted unrestricted roughage	restricted unrestricted roughage	link
189	gen.Engl.	1	quest	1	2439,0	1	NA	dayly	5	0	g	2	4,0	Arabian	Arabian horse	3	4000	1	2	group	2	unrestricted	2	unrestricted	2	unrestricted	
190	gen.Engl.	1	quest	1	2444,0	1		dayly	5		g	2	7,0	Thoroughbred	Thoroughbre	5	250	2	1	single	2	unrestricted	2	unrestricted	2	unrestricted	
191	gen.Engl.	1	quest	1	2504,0	1	NA	more than 20 t	4	0	g	2	8,0	Arabian	Arabian horse	3	4000	1	2	group	2	unrestricted	2	unrestricted	2	unrestricted	
192	gen.Engl.	1	quest	1	2506,0	1	NA	2-10 times	2	0	m	1	13,0	Appaloosa	Warmblood	1	350	3	2	group	2	unrestricted	2	unrestricted	1	restricted	
193	gen. Frensh	3	quest	1	2502,0	1		more than 20 times	4		m	1	6,0	Selle Francais	Warmblood	1	350	3	1	single	2	unrestricted	2	unrestricted	1	restricted	
194	gen. Frensh	3	quest	1	2499,0	1	1	dayly	5	0	g	2	18,0	Arabian	Arabian horse	3	4000	1	1	single	2	unrestricted	2	unrestricted	2	unrestricted	
195	gen. Frensh	3	quest	1	2500,0	1	0	dayly	5	1	m	1	6,0	Connemara	Pony	2	100	5	1	single	2	unrestricted	2	unrestricted	1	restricted	
196	door_gate_Gern	4	video	2	2181,0	1	NA	NA			NA			NA	Warmblood	1	350	3									https://www.youtube.com/results?q=clever+horse&sp=S
197	door_gate_Gern	4	video	2	2179,0	3	NA	NA			m	1		NA	Arabian horse	3	4000	1	1	single							https://www.youtube.com/watch?v=nDmEs_gRKgQ
198	door_gate_Gern	4	video	2	2176,0	1	NA	NA			s	3		NA	Warmblood	1	350	3	1	single							https://www.youtube.com/watch?v=LZ91lg3yZks
199	door_gate_Gern	4	video	2	2177,0	1	NA	NA			m	1		NA	Arabian horse	3	4000	1	1	single							https://www.youtube.com/watch?v=6pibfNWwVXg
200	door_gate_Gern	4	quest_dc	3	2184,0	3	open door, unty itself	more than 20 t	4	0	g	2	14,0	NA	Draught horse	4	500	4	2	group	2	unrestricted	2	unrestricted	1	restricted	
201	door_gate_Gern	4	quest_dc	3	2187,0	1	electric fence tap	more than 20 t	4		g	2	5,0	NA	Warmblood	1	350	3	1	single	2	unrestricted	2	unrestricted	2	unrestricted	
202	door_gate_Gern	4	quest_dc	3	2189,0	2	NA	more than 20 t	4	0	g	2	7,0	NA	Warmblood	1	350	3	1	single	1	restricted	2	unrestricted	2	unrestricted	
203	door_gate_Gern	4	quest_dc	3	2192,0	1	NA	more than 20 t	4	0	m	1	11,0	NA	Warmblood	1	350	3	1	single	1	restricted	1	restricted	1	restricted	
204	door_gate_Gern	4	quest_dc	3	2195,0	1	NA	more than 20 t	4	0	g	2	24,0	NA	Warmblood	1	350	3	2	group	1	restricted	2	unrestricted	2	unrestricted	
205	door_gate_Gern	4	quest_dc	3	2197,0	1	NA	11-20 times	3	0	s	3	24,0	NA	Warmblood	1	350	3	1	single	1	restricted	1	restricted	1	restricted	
206	door_gate_Gern	4	quest_dc	3	2201,0	1	open door, open gate	2-10 times	2	0	g	2	5,0	NA	Warmblood	1	350	3	1	single	1	restricted	1	restricted	1	restricted	
207	door_gate_Gern	4	quest_dc	3	2203,0	1	Bem. feeds from feed bins of other horses when he frees	2-10 times	2	0	g	2	17,0	NA	Draught horse	4	500	4	1	single	1	restricted	2	unrestricted	2	unrestricted	
208	door_gate_Gern	4	video	2	2204,0	1	NA	NA			NA			NA	Warmblood	1	350	3	1	single							https://www.youtube.com/watch?v=Y6580YcRQyI
209	door_gate_Gern	4	video	2	2205,0	1	electric fence taps	NA			NA			NA	Warmblood	1	350	3									https://www.youtube.com/watch?v=IxMuQiFQN98
210	door_gate_Gern	4	quest_dc	3	2210,0	1	open gate, open door	dayly	5		g	2	18,0	NA	Pony	2	100	5	2	group	2	unrestricted	2	unrestricted	2	unrestricted	
211	door_gate_Gern	4	quest_dc	3	2212,0	3	NA	more than 20 t	4	0	g	2	3,0	NA	Warmblood	1	350	3	1	single	2	unrestricted	1	restricted	2	unrestricted	
212	door_gate_Gern	4	quest_dc	3	2213,0	1	NA	more than 20 t	4	0	g	2	20,0	NA	Warmblood	1	350	3	2	group	2	unrestricted	2	unrestricted	2	unrestricted	
213	door_gate_Gern	4	quest_dc	3	2215,0	4	open door, open gate	11-20 times	3	0	g	2	15,0	NA	Warmblood	1	350	3	2	group	1	restricted	2	unrestricted	2	unrestricted	
214	door_gate_Gern	4	quest_dc	3	2220,0	1	open gate, open electric fence taps	more than 20 t	4	0	m	1	6,0	NA	Warmblood	1	350	3	2	group	2	unrestricted	2	unrestricted	2	unrestricted	
215	door_gate_Gern	4	quest_dc	3	2224,0	2	open door, close lock of trailor	more than 20 t	4	0	m	1	10,0	NA	Warmblood	1	350	3	1	single	1	restricted	1	restricted	2	unrestricted	
216	door_gate_Gern	4	quest_dc	3	2227,0	1	NA	11-20 times	3	0	g	2	6,0	NA	Warmblood	1	350	3	1	single	1	restricted	1	restricted	1	restricted	
217	door_gate_Gern	4	quest_dc	3	2228,0	1	open door, open gate	11-20 times	3	1	g	2	4,0	NA	Draught horse	4	500	4	1	single	1	restricted	1	restricted	2	unrestricted	
218	door_gate_Gern	4	quest_dc	3	2230,0	2	NA	dayly	5	0	m	1	10,0	NA	Warmblood	1	350	3	2	group	2	unrestricted	2	unrestricted	1	restricted	
219	door_gate_Gern	4	quest_dc	3	2232,0	2	NA	more than 20 t	4	0	m	1	18,0	NA	Warmblood	1	350	3	2	group	2	unrestricted	2	unrestricted	1	restricted	
220	door_gate_Gern	4	quest_dc	3	2234,0	1	NA	2-10 times	2	0	g	2	1,0	NA	Warmblood	1	350	3	2	group	1	restricted	2	unrestricted	2	unrestricted	
221	door_gate_Gern	4	quest_dc	3	2236,0	1	NA	NA		0	g	2	14,0	NA	Warmblood	1	350	3	1	single	2	unrestricted	1	restricted	1	restricted	
222	door_gate_Gern	4	quest_dc	3	2237,0	2	NA	dayly	5	0	g	2	17,0	NA	Warmblood	1	350	3	1	single	2	unrestricted	1	restricted	1	restricted	
223	door_gate_Gern	4	quest_dc	3	2239,0	1	NA	more than 20 t	4	0	g	2	8,0	NA	Warmblood	1	350	3	1	single	2	unrestricted	1	restricted	2	unrestricted	
224	door_gate_Gern	4	quest_dc	3	2240,0	1	NA	more than 20 t	4	0	g	2	7,0	NA	Pony	2	100	5	2	group			2	unrestricted	1	restricted	
225	door_gate_Gern	4	quest_dc	3	2241,0	1	NA	more than 20 t	4	0	m	1	19,0	NA	Warmblood	1	350	3	1	single	2	unrestricted	1	restricted	1	restricted	
226	door_gate_Gern	4	quest_dc	3	2244,0	3	NA	more than 20 t	4	0	m	1	21,0	NA	Warmblood	1	350	3	1	single	2	unrestricted	1	restricted	1	restricted	
227	door_gate_Gern	4	quest_dc	3	2246,0	1	NA	more than 20 t	4	0	g	2	6,0	NA	Warmblood	1	350	3	2	group	2	unrestricted	2	unrestricted	1	restricted	
228	door_gate_Gern	4	quest_dc	3	2249,0	1	NA	2-10 times	2	1	m	1	17,0	NA	Warmblood	1	350	3	2	group	2	unrestricted	2	unrestricted	2	unrestricted	

1	sorce	sorce num	video/ quest	video_quest_num	sID	nr reported behaviours	type of other behaviours	how often was the behaviour shown	behaviour frequency	social learning (spreading to other horses)	sex	sex num	age	breed	breed-type	breed type num	length of domestication	purpose of domestication	single group stabling	single group stabling	restricted unrestricted pasture	restricted unrestricted pasture	restricted unrestricted contact with horses	restricted unrestricted contact with horses	restricted unrestricted roughage	restricted unrestricted roughage	link
229	door_gate_Gern	4	quest_dc	3	2252,0	1	NA	dayly	5	0	g	2	5,0	NA	Warmblood	1	350	3	2	group	2	unrestricted	2	unrestricted	1	restricted	
230	door_gate_Gern	4	quest_dc	3	2255,0	1	NA	2-10 times	2	0	g	2	7,0	NA	Warmblood	1	350	3	1	single	1	restricted	1	restricted	1	restricted	
231	door_gate_Gern	4	quest_dc	3	2256,0	1	NA	more than 20 t	4	0	s	3	3,0	NA	Draught horse	4	500	4	2	group	2	unrestricted	2	unrestricted	2	unrestricted	
232	door_gate_Gern	4	quest_dc	3	2257,0	1	NA	NA		0	g	2	5,0	NA	Warmblood	1	350	3	2	group	2	unrestricted	2	unrestricted	2	unrestricted	
233	door_gate_Gern	4	quest_dc	3	2264,0	2	NA	dayly	5	0	m	1	4,0	NA	Arabian horse	3	4000	1	2	group	2	unrestricted	2	unrestricted	1	restricted	
234	door_gate_Gern	4	quest_dc	3	2280,0	3	open door, open gate	more than 20 t	4	0	g	2	12,0	NA	Warmblood	1	350	3	2	group	2	unrestricted	2	unrestricted	2	unrestricted	
235	door_gate_Gern	4	quest_dc	3	2284,0	1	NA	2-10 times	2	0	g	2	11,0	NA	Warmblood	1	350	3	1	single	2	unrestricted	1	restricted	1	restricted	
236	door_gate_Gern	4	quest_dc	3	2286,0	1	NA	dayly	5	0	g	2	10,0	NA	Warmblood	1	350	3	2	group	2	unrestricted	2	unrestricted	1	restricted	
237	door_gate_Gern	4	quest_dc	3	2287,0	1	open door, open gate	more than 20 t	4	0	g	2	10,0	NA	Pony	2	100	5	2	group	2	unrestricted	2	unrestricted	1	restricted	
238	door_gate_Gern	4	quest_dc	3	2289,0	2	open door	more than 20 t	4	0	g	2	8,0	NA	Draught horse	4	500	4	1	single			1	restricted	2	unrestricted	
239	door_gate_Gern	4	quest_dc	3	2290,0	3	open door, open gate, turn light switch	more than 20 t	4	0	g	2	12,0	NA	Warmblood	1	350	3	1	single	1	restricted	1	restricted	2	unrestricted	
240	door_gate_Gern	4	quest_dc	3	2291,0	1	NA	more than 20 t	4	0	g	2	28,0	NA	Warmblood	1	350	3	2	group	2	unrestricted	2	unrestricted	1	restricted	
241	door_gate_Gern	4	quest_dc	3	2292,0	2	open door, unty itself	2-10 times	2	1	g	2	12,0	NA	Warmblood	1	350	3	2	group	2	unrestricted	2	unrestricted	2	unrestricted	
242	door_gate_Gern	4	quest_dc	3	2293,0	2	open door, open	11-20 times	3	0	g	2	16,0	NA	Warmblood	1	350	3	1	single	1	restricted	1	restricted	1	restricted	
243	door_gate_Gern	4	quest_dc	3	2294,0	2	NA	2-10 times	2	0	g	2	10,0	NA	NA				1	single	1	restricted	1	restricted	1	restricted	
244	door_gate_Gern	4	quest_dc	3	2295,1	2	NA	more than 20 t	4	1	g	2	11,0	NA	Warmblood	1	350	3	1	single	1	restricted	1	restricted	1	restricted	
245	door_gate_Gern	4	quest_dc	3	2296,0	1	NA	2-10 times	2	0	m	1	5,0	NA	Warmblood	1	350	3	2	group	1	restricted	2	unrestricted	1	restricted	
246	door_gate_Gern	4	quest_dc	3	2298,0	1	NA	dayly	5	0	g	2	4,0	NA	Warmblood	1	350	3	2	group	2	unrestricted	2	unrestricted	1	restricted	
247	door_gate_Gern	4	quest_dc	3	2301,0	1	NA	more than 20 t	4	0	g	2	15,0	NA	Arabian horse	3	4000	1	2	group	1	restricted	2	unrestricted	1	restricted	
248	door_gate_Gern	4	quest_dc	3	2306,0	1	NA	2-10 times	2	0	g	2	8,0	NA	Draught horse	4	500	4	2	group	2	unrestricted	2	unrestricted	1	restricted	
249	door_gate_Gern	4	quest_dc	3	2307,0	3	open gate, open	2-10 times	2	0	g	2	15,0	NA	Pony	2	100	5	2	group	2	unrestricted	2	unrestricted	2	unrestricted	
250	door_gate_Gern	4	quest_dc	3	2309,0	2	NA	more than 20 t	4	0	m	1	10,0	NA	Warmblood	1	350	3	1	single			1	restricted	2	unrestricted	
251	door_gate_Gern	4	quest_dc	3	2310,0	2	NA	dayly	5	0	m	1	2,0	NA	Warmblood	1	350	3	2	group	2	unrestricted	2	unrestricted	2	unrestricted	
252	door_gate_Gern	4	quest_dc	3	2314,0	6	open door, unty itself and others, carries objects	2-10 times	2	0	g	2	3,0	NA	Pony	2	100	5	2	group	2	unrestricted	1	restricted	2	unrestricted	
253	door_gate_Gern	4	quest_dc	3	2316,0	1	NA	more than 20 t	4	0	g	2	10,0	NA	Draught horse	4	500	4	2	group	2	unrestricted	2	unrestricted	1	restricted	
254	door_gate_Gern	4	quest_dc	3	2318,0	1	NA	2-10 times	2	0	g	2	10,0	NA	Warmblood	1	350	3	1	single	1	restricted	1	restricted	1	restricted	
255	door_gate_Gern	4	quest_dc	3	2319,0	1	NA	dayly	5	0	g	2	4,0	NA	Warmblood	1	350	3	1	single	1	restricted	1	restricted	2	unrestricted	
256	door_gate_Gern	4	quest_dc	3	2320,0	1	NA	more than 20 t	4	0	s	3	27,0	NA	NA				2	group	1	restricted	2	unrestricted	1	restricted	
257	door_gate_Gern	4	quest_dc	3	2322,0	2	open gate, turn water clock	2-10 times	2	0	g	2	6,0	NA	Warmblood	1	350	3	2	group	2	unrestricted	2	unrestricted	1	restricted	
258	door_gate_Gern	4	quest_dc	3	2323,0	3	water clock, unty itself	11-20 times	3	0	m	1	9,0	NA	Warmblood	1	350	3	1	single	1	restricted	1	restricted	1	restricted	
259	door_gate_Gern	4	quest_dc	3	2324,0	1	NA	11-20 times	3	0	g	2	10,0	NA	Thoroughbred	5	250	2	1	single	1	restricted	1	restricted			
260	door_gate_Gern	4	quest_dc	3	2329,0	1	NA	dayly	5	0	g	2	15,0	NA	Warmblood	1	350	3	2	group	1	restricted	2	unrestricted	1	restricted	
261	door_gate_Gern	4	quest_dc	3	2332,0	2	NA	2-10 times	2	0	m	1	14,0	NA	NA				1	single	2	unrestricted	1	restricted	1	restricted	
262	door_gate_Gern	4	quest_dc	3	2333,0	1	NA	NA		1	g	2	10,0	NA	Pony	2	100	5	2	group	2	unrestricted	2	unrestricted	1	restricted	
263	door_gate_Gern	4	quest_dc	3	2334,0	3	NA	2-10 times	2	0	m	1	11,0	NA	Warmblood	1	350	3	1	single	1	restricted	1	restricted	2	unrestricted	
264	door_gate_Gern	4	quest_dc	3	2335,0	1	NA	more than 20 t	4	0	m	1	5,0	NA	Draught horse	4	500	4	1	single	2	unrestricted	1	restricted	1	restricted	
265	door_gate_Gern	4	quest_dc	3	2336,0	2	open door, unty itself	2-10 times	2		s	3	4,0	NA	Warmblood	1	350	3	2	group	2	unrestricted	2	unrestricted	2	unrestricted	
266	door_gate_Gern	4	quest_dc	3	2338,0	1	NA	2-10 times	2	0	g	2	7,0	NA	Warmblood	1	350	3	1	single	1	restricted	1	restricted	2	unrestricted	
267	door_gate_Gern	4	quest_dc	3	2341,0	2	NA	dayly	5	0	m	1	6,0	NA	Warmblood	1	350	3	2	group	2	unrestricted	2	unrestricted	2	unrestricted	
268	door_gate_Gern	4	quest_dc	3	2343,0	1	NA	2-10 times	2	0	s	3	22,0	NA	Warmblood	1	350	3	1	single	1	restricted	1	restricted	2	unrestricted	
269	door_gate_Gern	4	quest_dc	3	2340,0	2	open door, open water clock	more than 20 t	4	0	g	2	3,0	NA	Warmblood	1	350	3	2	group	2	unrestricted	2	unrestricted	2	unrestricted	
270	door_gate_Gern	4	quest_dc	3	2344,0	2	open door, unty itself	2-10 times	2	0	g	2	6,0	NA	Warmblood	1	350	3	1	single	1	restricted	1	restricted	2	unrestricted	

1	sorce	sorce num	video / quest	video_quest_num	sID	nr reported behaviours	type of other behaviours	how often was the behaviour shown	behaviour frequency	social learning (spreading to other horses)	sex	sex num	age	breed	breed-type	breed type num	length of domestication	purpose of domestication	single group stabling	single group stabling	restricted unrestricted pasture	restricted unrestricted pasture	restricted unrestricted contact with horses	restricted unrestricted contact with horses	restricted unrestricted roughage	restricted unrestricted roughage	link
271	door_gate_Gern	4	quest_dc	3	2345,0	2	NA	dayly	5	1	m	1	10,0	NA	Warmblood	1	350	3	2	group	2	unrestricted	2	unrestricted	1	restricted	
272	door_gate_Gern	4	quest_dc	3	2346,0	2	open door, open gate	more than 20 (	4	0	g	2	18,0	NA	Warmblood	1	350	3	2	group	2	unrestricted	2	unrestricted	2	unrestricted	
273	door_gate_Gern	4	quest_dc	3	2350,0	3	open door, unty itself	2-10 times	2	0	g	2	21,0	NA	NA				1	single	1	restricted	1	restricted	1	restricted	
274	door_gate_Gern	4	quest_dc	3	2356,0	1	NA	2-10 times	2	0	m	1	18,0	NA	Pony	2	100	5	2	group	1	restricted	2	unrestricted	1	restricted	
275	door_gate_Gern	4	quest_dc	3	2357,0	3	Na	2-10 times	2	0	g	2	9,0	NA	Warmblood	1	350	3	2	group	2	unrestricted	2	unrestricted	1	restricted	
276	door_gate_Gern	4	quest_dc	3	2358,0	2	NA	11-20 times	3	0	g	2	4,0	NA	Draught horse	4	500	4	1	single	1	restricted	1	restricted	1	restricted	
277	door_gate_Gern	4	quest_dc	3	2361,0	3	NA	11-20 times	3	0	g	2	5,0	NA	Draught horse	4	500	4	2	group	2	unrestricted	2	unrestricted	2	unrestricted	
278	door_gate_Gern	4	quest_dc	3	2363,0	1	NA	more than 20 (	4	0	g	2	10,0	NA	Warmblood	1	350	3	1	single	1	restricted	1	restricted	1	restricted	
279	door_gate_Gern	4	quest_dc	3	2364,0	3	NA	dayly	5	0	g	2	7,0	NA	Warmblood	1	350	3	2	group	2	unrestricted	2	unrestricted	2	unrestricted	
280	door_gate_Gern	4	quest_dc	3	2365,0	1	NA	more than 20 (	4	2	g	2	4,0	NA	Arabian horse	3	4000	1	2	group	1	restricted	2	unrestricted	1	restricted	
281	door_gate_Gern	4	quest_dc	3	2366,0	2	NA	2-10 times	2	0	m	1	9,0	NA	Warmblood	1	350	3	1	single	2	unrestricted	1	restricted	2	unrestricted	
282	door_gate_Gern	4	quest_dc	3	2367,0	2	NA	2-10 times	2	0	m	1	8,0	NA	NA				1	single	1	restricted	1	restricted			
283	door_gate_Gern	4	quest_dc	3	2368,0	3	NA	more than 20 (	4	0	m	1	21,0	NA	Donkey	7	6000	4	2	group	2	unrestricted	2	unrestricted	1	restricted	
284	door_gate_Gern	4	quest_dc	3	2370,0	1	NA	2-10 times	2	0	g	2	5,0	NA	Pony	2	100	5	2	group	2	unrestricted	2	unrestricted	2	unrestricted	
285	door_gate_Gern	4	quest_dc	3	2371,0	1	NA	NA		0	g	2	8,0	NA	Warmblood	1	350	3	2	group	2	unrestricted	2	unrestricted	1	restricted	
286	door_gate_Gern	4	quest_dc	3	2373,0	3	NA	2-10 times	2		g	2	11,0	NA	Draught horse	4	500	4	2	group	2	unrestricted	2	unrestricted	2	unrestricted	
287	door_gate_Gern	4	quest_dc	3	2374,0	2	open door, unty itself	2-10 times	2	2	g	2	8,0	NA	Warmblood	1	350	3	1	single	1	restricted	1	restricted	1	restricted	
288	door_gate_Gern	4	quest_dc	3	2379,0	2	NA	2-10 times	2		m	1	22,0	NA	Pony	2	100	5	2	group	2	unrestricted	2	unrestricted	1	restricted	
289	door_gate_Gern	4	quest_dc	3	2379,0	2	NA	2-10 times	2		NA		1,0	NA	Mule	6	3000	4	2	group	2	unrestricted	2	unrestricted	1	restricted	
290	door_gate_Gern	4	quest_dc	3	2381,0	3	NA	11-20 times	3	0	g	2	27,0	NA	Pony	2	100	5	2	group	2	unrestricted	2	unrestricted	1	restricted	
291	door_gate_Gern	4	quest_dc	3	2383,0	2	NA	2-10 times	2	0	g	2	16,0	NA	Pony	2	100	5	2	group	2	unrestricted	2	unrestricted	1	restricted	
292	door_gate_Gern	4	quest_dc	3	2385,0	1	NA	more than 20 (	4	0	g	2	2,0	NA	Warmblood	1	350	3	1	single	2	unrestricted	1	restricted	1	restricted	
293	door_gate_Gern	4	quest_dc	3	2389,0	1	open door, open gate	more than 20 (	4	0	g	2	7,0	NA	Warmblood	1	350	3	2	group	2	unrestricted	2	unrestricted	2	unrestricted	
294	door_gate_Gern	4	quest_dc	3	2390,0	5	open door, unty itself	11-20 times	3	0	g	2	9,0	NA	Pony	2	100	5	1	single	1	restricted	1	restricted	2	unrestricted	
295	door_gate_Gern	4	quest_dc	3	2391,0	2	NA	dayly	5	0	g	2	13,0	NA	Draught horse	4	500	4	2	group	2	unrestricted	2	unrestricted	2	unrestricted	
296	door_gate_Gern	4	quest_dc	3	2393,0	1	NA	more than 20 (	4	0	g	2	3,0	NA	Warmblood	1	350	3	1	single	1	restricted	1	restricted	1	restricted	
297	door_gate_Gern	4	quest_dc	3	2396,0	1	open door, open gate	2-10 times	2	0	g	2	8,0	NA	Warmblood	1	350	3	1	single	1	restricted	1	restricted	1	restricted	
298	door_gate_Gern	4	quest_dc	3	2397,0	3	NA	more than 20 (	4	0	m	1	5,0	NA	Pony	2	100	5	2	group	2	unrestricted	2	unrestricted	1	restricted	
299	door_gate_Gern	4	quest_dc	3	2408,0	1	NA	2-10 times	2	0	m	1	7,0	NA	Arabian horse	3	4000	1	1	single	1	restricted	1	restricted	1	restricted	
300	door_gate_Gern	4	quest_dc	3	2409,0	1	NA	2-10 times	2	0	g	2	7,0	NA	Warmblood	1	350	3	1	single	2	unrestricted	2	unrestricted	2	unrestricted	
301	door_gate_Gern	4	quest_dc	3	2410,0	1	NA	11-20 times	3	0	m	1	10,0	NA	Warmblood	1	350	3	1	single	1	restricted	1	restricted	1	restricted	
302	door_gate_Gern	4	quest_dc	3	2411,0	1	NA	2-10 times	2	0	g	2	5,0	NA	Arabian horse	3	4000	1	2	group	2	unrestricted	2	unrestricted	2	unrestricted	
303	door_gate_Gern	4	quest_dc	3	2418,0	1	NA	2-10 times	2	0	g	2	12,0	NA	Warmblood	1	350	3	2	group	2	unrestricted	2	unrestricted	2	unrestricted	
304	door_gate_Gern	4	quest_dc	3	2419,0	1	NA	2-10 times	2	0	m	1	8,0	NA	Warmblood	1	350	3	1	single	1	restricted	1	restricted	1	restricted	
305	door_gate_Gern	4	quest_dc	3	2420,0	5	open boots, unty itself, take off rugs, apport things	NA		0	g	2	11,0	NA	Warmblood	1	350	3	2	group	1	restricted	2	unrestricted	2	unrestricted	
306	door_gate_Gern	4	quest_dc	3	2421,0	3	NA	more than 20 (	4	0	g	2	22,0	NA	Warmblood	1	350	3	2	group	2	unrestricted	2	unrestricted	1	restricted	
307	door_gate_Gern	4	quest_dc	3	2422,0	1	NA	2-10 times	2	0	g	2	7,0	NA	Pony	2	100	5	2	group	2	unrestricted	2	unrestricted	2	unrestricted	
308	door_gate_Gern	4	quest_dc	3	2425,0	2	shut water tap	11-20 times	3	0	g	2	3,0	NA	Warmblood	1	350	3	1	single	1	restricted	1	restricted	1	restricted	
309	door_gate_Gern	4	quest_dc	3	2427,0	1	NA	more than 20 (	4	0	s	3	5,0	NA	Warmblood	1	350	3	2	group	1	restricted	2	unrestricted	2	unrestricted	
310	door_gate_Gern	4	quest_dc	3	2432,0	1	NA	2-10 times	2	0	g	2	13,0	NA	Pony	2	100	5	2	group	2	unrestricted	2	unrestricted	1	restricted	
311	door_gate_Gern	4	quest_dc	3	2434,0	2	NA	dayly	5	0	g	2	13,0	NA	Warmblood	1	350	3	2	group	2	unrestricted	2	unrestricted	1	restricted	
312	door_gate_Gern	4	quest_dc	3	2435,0	2	NA	2-10 times	2	1	g	2	2,0	NA	Warmblood	1	350	3	1	single	1	restricted	1	restricted	2	unrestricted	
313	door_gate_Gern	4	quest_dc	3	2436,0	2	NA	more than 20 (	4	0	m	1	11,0	NA	Warmblood	1	350	3	2	group	1	restricted	2	unrestricted	2	unrestricted	
314	door_gate_Gern	4	quest_dc	3	2437,0	3	NA	dayly	5	0	m	1	5,0	NA	Warmblood	1	350	3	1	single	1	restricted	1	restricted	1	restricted	
315	door_gate_Gern	4	quest_dc	3	2443,0	1	NA	2-10 times	2	0	s	3	20,0	NA	Warmblood	1	350	3	1	single	1	restricted	1	restricted	1	restricted	
[illegible]	[illegible]	[illegible]	[illegible]	[illegible]	[illegible]	[illegible]	[illegible]	[illegible]	[illegible]	[illegible]	[illegible]	[illegible]	[illegible]	[illegible]	[illegible]	[illegible]	[illegible]	[illegible]	[illegible]	[illegible]	[illegible]	[illegible]	[illegible]	[illegible]	[illegible]	[illegible]	[illegible]

1	sorce	sorce num	video / quest	video_quest_num	sID	nr reported behaviours	type of other behaviours	how often was the behaviour shown	behaviour frequency	social learning (spreading to other horses)	sex	sex num	age	breed	breed-type	breed type num	length of domestication	purpose of domestication	single group stabling	single group stabling	restricted unrestricted pasture	restricted unrestricted pasture	restricted unrestricted contact with horses	restricted unrestricted contact with horses	restricted unrestricted roughage	restricted unrestricted roughage	link
316	door_gate_Gern	4	quest_dc	3	2446,0	1	NA	more than 20 (	4	0	m	1	26,0	NA	Warmblood	1	350	3	1	single	1	restricted	1	restricted	1	restricted	
317	door_gate_Gern	4	quest_dc	3	2448,0	1	NA	2-10 times	2	0	g	2	5,0	NA	Pony	2	100	5	2	group	1	restricted	2	unrestricted	1	restricted	
318	door_gate_Gern	4	quest_dc	3	2449,0	2	open door, open gate	2-10 times	2		g	2	13,0	NA	Pony	2	100	5	2	group	1	restricted	2	unrestricted	1	restricted	
319	door_gate_Gern	4	quest_dc	3	2451,0	3	NA	more than 20 (	4	0	m	1	5,0	NA	Warmblood	1	350	3	2	group	2	unrestricted	2	unrestricted	1	restricted	
320	door_gate_Gern	4	quest_dc	3	2454,0	1	NA	more than 20 (	4	0	g	2	3,0	NA	Arabian horse	3	4000	1	2	group	2	unrestricted	2	unrestricted	2	unrestricted	
321	door_gate_Gern	4	quest_dc	3	2509,0	1	NA	11-20 times	3	1	m	1	0,5	NA	Arabian horse	3	4000	1	2	group	2	unrestricted	2	unrestricted	1	restricted	
322	door_gate_Gern	4	video	2	2510,0	1	NA	more than 20 (	4	0	NA			NA	Donkey	7	6000	4	2	group	2	unrestricted	2	unrestricted	1	restricted	https://www.youtube.com/watch?v=sa5cgpMO0xA
323	door_gate_Engl	5	video	2	2206,0	1	NA	NA			g	2		NA	Warmblood	1	350	3	1	single							https://www.youtube.com/watch?v=ez6nOMYfclc
324	door_gate_Engl	5	video	2	2207,0	1	NA	NA			g	2		NA	Warmblood	1	350	3	1	single							https://www.youtube.com/watch?v=owjvgT8H4g4
325	door_gate_Engl	5	video	2	2208,0	1	NA	NA			NA			NA	Warmblood	1	350	3	1	single							https://www.youtube.com/watch?v=d_xe1lNWkq0
326	door_gate_Engl	5	video	2	2209,0	1	NA	NA			m	1		NA	Arabian horse	3	4000	1	1	single							https://www.youtube.com/watch?v=5XUuQ68bbh0
327	door_gate_Engl	5	quest_dc	3	2245,0	2	open door, open gate	more than 20 (	4	0	g	2	8,0	NA	Pony	2	100	5	2	group	2	unrestricted	2	unrestricted	2	unrestricted	
328	door_gate_Engl	5	quest_dc	3	2259,0	2	open door, open gate , take of fly protection (head)	11-20 times	3	0	g	2	5,0	NA	Warmblood	1	350	3	1	single	1	restricted	1	restricted	2	unrestricted	
329	door_gate_Engl	5	quest_dc	3	2263,0	2	NA	dayly	5	1	m	1	7,0	NA	Warmblood	1	350	3	2	group	2	unrestricted	2	unrestricted	2	unrestricted	
330	door_gate_Engl	5	quest_dc	3	2266,0	2	open door, open gate	11-20 times	3	0	g	2	7,0	NA	Warmblood	1	350	3	1	single	1	restricted	1	restricted	2	unrestricted	
331	door_gate_Engl	5	quest_dc	3	2267,0	2	NA	11-20 times	3	1	m	1	20,0	NA	Warmblood	1	350	3	2	group	2	unrestricted	2	unrestricted	1	restricted	
332	door_gate_Engl	5	quest_dc	3	2269,0	3	open door, open gate	dayly	5	0	m	1	5,0	NA	Warmblood	1	350	3	1	single	1	restricted	1	restricted	2	unrestricted	
333	door_gate_Engl	5	quest_dc	3	2271,0	6	open door, open gate, unty itself, take off halter	dayly	5	1	g	2	10,0	NA	Warmblood	1	350	3	1	single	1	restricted	1	restricted	1	restricted	
334	door_gate_Engl	5	quest_dc	3	2273,0	2	NA	more than 20 (	4	0	g	2	9,0	NA	Warmblood	1	350	3	1	single	2	unrestricted	1	restricted	2	unrestricted	
335	door_gate_Engl	5	quest_dc	3	2274,0	2	NA	NA		0	m	1	7,0	NA	Arabian horse	3	4000	1	1	single	1	restricted	1	restricted	1	restricted	
336	door_gate_Engl	5	quest_dc	3	2277,0	1	NA	2-10 times	2	0	m	1	4,0	NA	Warmblood	1	350	3	2	group	2	unrestricted	2	unrestricted	1	restricted	
337	door_gate_Engl	5	quest_dc	3	2278,0	3	NA	more than 20 (	4	0	m	1	4,0	NA	Warmblood	1	350	3	2	group	2	unrestricted	2	unrestricted	2	unrestricted	
338	door_gate_Engl	5	quest_dc	3	2317,0	4	open door, open gate	11-20 times	3	0	g	2	7,0	NA	Draught horse	4	500	4	2	group	2	unrestricted	2	unrestricted	1	restricted	
339	door_gate_Engl	5	quest_dc	3	2321,0	1	NA	more than 20 (	4	0	NA			NA	NA				1	single	2	unrestricted	1	restricted	2	unrestricted	
340	door_gate_Engl	5	quest_dc	3	2330,0	4	open door, open gate	11-20 times	3	0	g	2	5,0	NA	Warmblood	1	350	3	1	single	1	restricted	1	restricted	2	unrestricted	
341	door_gate_Engl	5	quest_dc	3	2330,1	2	NA	11-20 times	3	0	NA			NA	Pony	2	100	5	1	single	1	restricted	1	restricted	2	unrestricted	
342	door_gate_Engl	5	quest_dc	3	2339,0	1	NA	11-20 times	3	0	m	1	25,0	NA	Pony	2	100	5	1	single	1	restricted	1	restricted	1	restricted	
343	door_gate_Engl	5	quest_dc	3	2347,0	2	NA	dayly	5	0	g	2	10,0	NA	Warmblood	1	350	3	1	single	2	unrestricted	1	restricted	2	unrestricted	
344	door_gate_Engl	5	quest_dc	3	2353,0	3	NA	more than 20 (	4	1	g	2	28,0	NA	Warmblood	1	350	3	1	single	1	restricted	1	restricted	2	unrestricted	
345	door_gate_Engl	5	quest_dc	3	2355,0	1	NA	2-10 times	2	0	m	1	7,0	NA	Thoroughbre	5	250	2	1	single	1	restricted	1	restricted	2	unrestricted	
346	door_gate_Engl	5	quest_dc	3	2360,0	2	NA	NA		0	g	2	13,0	NA	Warmblood	1	350	3	1	single	1	restricted	1	restricted	1	restricted	
347	door_gate_Engl	5	quest_dc	3	2362,0	1	NA	2-10 times	2	0	g	2	10,0	NA	Warmblood	1	350	3	1	single	1	restricted	1	restricted	2	unrestricted	
348	door_gate_Engl	5	quest_dc	3	2369,0	3	NA	dayly	5	0	g	2	6,0	NA	Warmblood	1	350	3	2	group	1	restricted	2	unrestricted	1	restricted	
349	door_gate_Engl	5	quest_dc	3	2380,0	4	NA	more than 20 (	4	0	m	1	18,0	NA	Thoroughbre	5	250	2	1	single	1	restricted	1	restricted	2	unrestricted	
350	door_gate_Engl	5	quest_dc	3	2395,0	3	NA	more than 20 (	4	0	m	1	16,0	NA	Warmblood	1	350	3	2	group	2	unrestricted	2	unrestricted	2	unrestricted	
351	door_gate_Engl	5	quest_dc	3	2398,0	1	NA	more than 20 (	4	0	m	1	3,0	NA	Pony	2	100	5	1	single	1	restricted	1	restricted	1	restricted	
352	door_gate_Engl	5	quest_dc	3	2403,0	1	open door, open gate	2-10 times	2	0	g	2	19,0	NA	Warmblood	1	350	3	1	single	2	unrestricted	1	restricted	2	unrestricted	
353	door_gate_Engl	5	quest_dc	3	2414,0	1	NA	2-10 times	2	1	s	3	21,0	NA	Arabian horse	3	4000	1	1	single	1	restricted	1	restricted	2	unrestricted	
354	door_gate_Engl	5	quest_dc	3	2423,0	3	NA	2-10 times	2	0	m	1	13,0	NA	Draught horse	4	500	4	2	group	2	unrestricted	2	unrestricted	2	unrestricted	
355	door_gate_Engl	5	quest_dc	3	2423,1	2	NA	2-10 times	2	0	g	2		NA	Warmblood	1	350	3	2	group	2	unrestricted	2	unrestricted	2	unrestricted	
356	door_gate_Engl	5	quest_dc	3	2424,0	2	NA	2-10 times	2	0	g	2	8,0	NA	Warmblood	1	350	3	1	single	2	unrestricted	1	restricted	2	unrestricted	
357	door_gate_Engl	5	quest_dc	3	2429,0	1	NA	more than 20 (	4	0	g	2	5,0	NA	Warmblood	1	350	3	1	single	1	restricted	1	restricted	2	unrestricted	

1	sorce	sorce num	video / quest	video_quest_num	sID	nr reported behaviours	type of other behaviours	how often was the behaviour shown	behaviour frequency	social learning (spreading to other horses)	sex	sex num	age	breed	breed-type	breed type num	length of domestication	purpose of domestication	single group stabling	single group stabling	restricted unrestricted pasture	restricted unrestricted pasture	restricted unrestricted contact with horses	restricted unrestricted contact with horses	restricted unrestricted roughage	restricted unrestricted roughage	link
358	door_gate_Engl	5	quest_dc	3	2430,0	1	NA	2-10 times	2	0	g	2	4,0	NA	Pony	2	100	5	2	group	1	restricted	2	unrestricted	1	restricted	
359	door_gate_Engl	5	quest_dc	3	2438,0	1	NA	2-10 times	2	0	m	1	2,0	NA	Arabian horse	3	4000	1	2	group	2	unrestricted	2	unrestricted	1	restricted	
360	door_gate_Engl	5	quest_dc	3	2440,0	4	open gate, unty itself	dayly	5	1	g	2	6,0	NA	Warmblood	1	350	3	2	group	2	unrestricted	2	unrestricted	2	unrestricted	
361	door_gate_Engl	5	quest_dc	3	2458,0	4	himself, remove fence	more than 20 t	4	0	g	2	30,0	NA	Warmblood	1	350	3	2	group	2	unrestricted	2	unrestricted	2	unrestricted	
362	door_gate_Engl	5	quest_dc	3	2478,0	4	NA	dayly	5	0	g	2	14,0	NA	Arabian horse	3	4000	1	1	single	1	restricted	1	restricted	2	unrestricted	
363	door_gate_Engl	5	quest_dc	3	2478,1	1	NA	dayly	5	0	m	1		NA	Arabian horse	3	4000	1	1	single	1	restricted	1	restricted	2	unrestricted	
364	door_gate_Engl	5	quest_dc	3	2505,0	12	untied halter knots, st	more than 20 t	4	0	g	2	2,0	NA	Arabian horse	3	4000	1	2	group	2	unrestricted	2	unrestricted	1	restricted	
365	door_gate_Engl	5	video	2	2507,0	2	NA	NA		0	NA			NA	Donkey	7	6000	4	2	group	1	restricted	2	unrestricted			https://www.youtube.com/watch?v=AN6I0Ehu7O0
366	door_gate_Engl	5	video	2	2508,0	1	NA	NA		0	NA			NA	Donkey	7	6000	4	1	single	2	unrestricted	1	restricted			https://www.youtube.com/watch?v=xEfotAYK_TE
367	door_gate_Engl	5	video	2	2511,0	1	NA	NA		0	g	2		NA	Mule	6	3000	4	1	single	1	restricted	1	restricted	1	restricted	https://www.youtube.com/watch?v=QiEYv4fA9vE
368	door_gate_Fren	6	video	2	2459,0	1	NA	dayly	5	0	g	2	7,0	NA	Warmblood	1	350	3	1	single	1	restricted	1	restricted	1	restricted	
369	door_gate_Fren	6	video	2	2471,0	2	NA	dayly	5	0	s	3	6,0	NA	NA				1	single	1	restricted	1	restricted	1	restricted	
370	door_gate_Fren	6	quest_dc	3	2469,0	2	NA	2-10 times	2		g	2	17,0	NA	Pony	2	100	5	1	single	1	restricted	1	restricted	1	restricted	
371	door_gate_Fren	6	quest_dc	3	2467,0	4	NA	more than 20 t	4	0	g	2	2,0	NA	Warmblood	1	350	3	1	single	1	restricted	1	restricted	1	restricted	
372	door_gate_Fren	6	quest_dc	3	2466,1	1	NA	11-20 times	3	1	m	1	2,0	NA	Arabian horse	3	4000	1	1	single	1	restricted	1	restricted	1	restricted	
373	door_gate_Fren	6	quest_dc	3	2465,0	2	NA	2-10 times	2		g	2	14,0	NA	Warmblood	1	350	3	1	single	1	restricted	1	restricted	2	unrestricted	
374	door_gate_Fren	6	quest_dc	3	2463,0	2	NA	11-20 times	3	0	g	2	28,0	NA	NA				2	group	2	unrestricted	2	unrestricted	1	restricted	
375	door_gate_Fren	6	quest_dc	3	2462,0	1	NA	2-10 times	2		g	2	13,0	NA	Warmblood	1	350	3	1	single	1	restricted	1	restricted	2	unrestricted	
376	door_gate_Fren	6	quest_dc	3	2461,0	2	NA	dayly	5	0	g	2	26,0	NA	Warmblood	1	350	3	1	single	1	restricted	1	restricted	1	restricted	
377	door_gate_Fren	6	quest_dc	3	2460,0	2	NA	more than 20 t	4	0	g	2	8,0	NA	NA				1	single	1	restricted	1	restricted	1	restricted	
378	door_gate_Fren	6	quest_dc	3	2472,0	1	NA	dayly	5	0	m	1	8,0	NA	Pony	2	100	5	1	single	1	restricted	1	restricted	2	unrestricted	
379	door_gate_Fren	6	quest_dc	3	2473,0	1	NA	dayly	5	0	m	1		NA	Arabian horse	3	4000	1	1	single	2	unrestricted	1	restricted	1	restricted	
380	door_gate_Fren	6	quest_dc	3	2476,0	2	NA	NA		0	g	2	18,0	NA	Arabian horse	3	4000	1	2	group	2	unrestricted	2	unrestricted	2	unrestricted	
381	door_gate_Fren	6	quest_dc	3	2477,0	2	NA	NA		0	g	2	7,0	NA	Warmblood	1	350	3	1	single	1	restricted	1	restricted	1	restricted	
382	door_gate_Fren	6	quest_dc	3	2479,0	1	NA	dayly	5	0	g	2	13,0	NA	Warmblood	1	350	3	1	single	1	restricted	1	restricted	1	restricted	
383	door_gate_Fren	6	quest_dc	3	2481,0	2	NA	11-20 times	3		g	2	11,0	NA	Warmblood	1	350	3	1	single	1	restricted	1	restricted	1	restricted	
384	door_gate_Fren	6	quest_dc	3	2483,0	1	NA	dayly	5	1	m	1	5,0	NA	NA				2	group	1	restricted	2	unrestricted	1	restricted	
385	door_gate_Fren	6	quest_dc	3	2485,0	2	NA	2-10 times	2	0	g	2	12,0	NA	Arabian horse	3	4000	1	1	single	1	restricted	1	restricted	1	restricted	
386	door_gate_Fren	6	quest_dc	3	2486,0	3	NA	dayly	5	0	g	2	4,0	NA	Warmblood	1	350	3	1	single	1	restricted	1	restricted	2	unrestricted	
387	door_gate_Fren	6	quest_dc	3	2486,1	1	NA	dayly	5	0	m	1		NA	Warmblood	1	350	3	1	single	1	restricted	1	restricted	2	unrestricted	
388	door_gate_Fren	6	quest_dc	3	2487,0	2	NA	2-10 times	2	0	g	2	10,0	NA	Warmblood	1	350	3	1	single	1	restricted	1	restricted	1	restricted	
389	door_gate_Fren	6	quest_dc	3	2488,0	2	NA	dayly	5	0	m	1	20,0	NA	Warmblood	1	350	3	1	single	1	restricted	1	restricted	1	restricted	
390	door_gate_Fren	6	quest_dc	3	2489,0	3	goes through electric fence	more than 20 t	4	0	m	1	16,0	NA	Warmblood	1	350	3	2	group	2	unrestricted	2	unrestricted	2	unrestricted	
391	door_gate_Fren	6	quest_dc	3	2494,0	2	NA	2-10 times	2	0	m	1	8,0	NA	Pony	2	100	5	2	group	2	unrestricted	2	unrestricted	2	unrestricted	
392	door_gate_Fren	6	quest_dc	3	2495,0	2	NA	2-10 times	2	0	g	2	9,0	NA	Warmblood	1	350	3	1	single	1	restricted	1	restricted	1	restricted	
393	door_gate_Fren	6	quest_dc	3	2496,0	3	NA	dayly	5	0	g	2	12,0	NA	Warmblood	1	350	3	1	single	1	restricted	1	restricted	1	restricted	
394	door_gate_Fren	6	quest_dc	3	2497,0	2	NA	more than 20 t	4	0	g	2	6,0	NA	Arabian horse	3	4000	1	2	group	2	unrestricted	2	unrestricted	2	unrestricted	
395	door_gate_Fren	6	quest_dc	3	2501,0	2	NA	more than 20 t	4	0	m	1	14,0	NA	Pony	2	100	5	2	group	2	unrestricted	2	unrestricted	2	unrestricted	
396	door_gate_Fren	6	quest_dc	3	2503,0	2	open door, open gate	more than 20 t	4	0	g	2	8,0	NA	Warmblood	1	350	3	2	group	1	restricted	2	unrestricted	1	restricted	
397	door_gate_Gern	4	video	2	2512,0	2	NA	NA			NA			NA	Warmblood	1	350	3	1	single	1	restricted	1	restricted	1	restricted	https://www.youtube.com/watch?v=IXebjTbLT4M

1	sorce	sorce num	video / quest	video_quest_num	sID	nr reported behaviours	type of other behaviours	how often was the behaviour shown	behaviour frequency	social learning (spreading to other horses)	sex	sex num	age	breed	breed-type	breed type num	length of domestication	purpose of domestication	single group stabling	single group stabling	restricted unrestricted pasture	restricted unrestricted pasture	restricted unrestricted contact with horses	restricted unrestricted contact with horses	restricted unrestricted roughage	restricted unrestricted roughage	link
398	door_gate_Gern	4	video	2	2513,0	1	NA	NA			NA			NA	Warmblood	1	350	3	1	single	1	restricted	1	restricted			https://www.youtube.com/watch?v=-R_e-zd5zr0
399	door_gate_Gern	4	video	2	2514,0	2	NA	NA			g	2		NA	Warmblood	1	350	3	1	single	1	restricted	1	restricted	1	restricted	https://www.youtube.com/watch?v=krnNwRdIkJo
400	door_gate_Gern	4	video	2	2515,0	1	NA	NA			g	2	6,0	NA	Arabian horse	3	4000	1	1	single	1	restricted	1	restricted	1	restricted	https://www.youtube.com/watch?v=bo60IBvt5jw
401	door_gate_Gern	4	video	2	2516,0	1	NA	NA			g	2		NA	Warmblood	1	350	3	1	single	1	restricted	1	restricted	1	restricted	https://www.youtube.com/watch?v=qBjIyl96E3g
402	door_gate_Gern	4	video	2	2517,0	1	NA	NA			NA			NA	Pony	2	100	5									https://www.youtube.com/watch?v=rZLbI5xcV9E
403	door_gate_Gern	4	video	2	2518,0	3	NA	NA			NA			NA	Warmblood	1	350	3	1	single	1	restricted	1	restricted	1	restricted	https://www.youtube.com/watch?v=W3KnTX0RI5s
404	door_gate_Gern	4	video	2	2538,0	1	NA	NA			NA			NA	Warmblood	1	350	3	1	single	1	restricted	1	restricted	1	restricted	https://www.youtube.com/watch?v=8IE1HHGuJ-E
405	door_gate_Gern	4	video	2	2539,0	1	NA	NA			m	1		NA	Warmblood	1	350	3	1	single	1	restricted	1	restricted			https://www.youtube.com/watch?v=QnVISUkqMAo
406	door_gate_Gern	4	video	2	2519,0	2	NA	NA			NA			NA	Warmblood	1	350	3	1	single	1	restricted	1	restricted	1	restricted	https://www.youtube.com/watch?v=0qUFaYN8-Ec
407	door_gate_Gern	4	video	2	2520,0	1	NA	NA			NA			NA	Warmblood	1	350	3	1	single	1	restricted	1	restricted			https://www.youtube.com/watch?v=ul-u7-_wA94
408	door_gate_Gern	4	video	2	2521,0	1	NA	NA			NA			NA	Warmblood	1	350	3	1	single	1	restricted	1	restricted	1	restricted	https://www.youtube.com/watch?v=A9V-ePJZfWo
409	door_gate_Gern	4	video	2	2522,0	1	NA	NA			NA			NA	Pony	2	100	5	1	single	1	restricted	1	restricted	1	restricted	https://www.youtube.com/watch?v=m3cIQNIGBQ4
410	door_gate_Gern	4	video	2	2523,0	1	NA	NA			NA		0,5	NA	NA												https://www.youtube.com/watch?v=YUe9gmUVQ6U
411	door_gate_Gern	4	video	2	2524,0	1	NA	NA			NA			NA	Warmblood	1	350	3	1	single	1	restricted	1	restricted	1	restricted	https://www.youtube.com/watch?v=Fg-wViXmc1Y
412	door_gate_Gern	4	video	2	2525,0	1	NA	NA			NA			NA	Warmblood	1	350	3	2	group			2	unrestricted			https://www.youtube.com/watch?v=VqYcJrye6VU
413	door_gate_Gern	4	video	2	2526,0	1	NA	NA			NA			NA	Warmblood	1	350	3	1	single	1	restricted	1	restricted			https://www.youtube.com/watch?v=g_DzG4vkmHc
414	door_gate_Gern	4	video	2	2527,0	1	NA	NA			g	2		NA	Warmblood	1	350	3	1	single	1	restricted	1	restricted			https://www.youtube.com/watch?v=zwsm07q3y7M
415	door_gate_Gern	4	video	2	2528,0	1	NA	NA			NA			NA	Pony	2	100	5									https://www.youtube.com/watch?v=EKAROqJvYDs
416	door_gate_Gern	4	video	2	2529,0	1	NA	NA			NA			NA	Pony	2	100	5									https://www.youtube.com/watch?v=Iu8LwlvZqvk
417	door_gate_Gern	4	video	2	2530,0	1	NA	NA			g	2		NA	Warmblood	1	350	3									https://www.youtube.com/watch?v=rDU4BIJFj9c
418	door_gate_Gern	4	video	2	2531,0	1	NA	NA			NA			NA	Warmblood	1	350	3	1	single	1	restricted	1	restricted			https://www.youtube.com/watch?v=AUGKbhZGTJw
419	door_gate_Gern	4	video	2	2532,0	1	NA	NA			m	1		NA	Pony	2	100	5									https://www.youtube.com/watch?v=8JIrxgDxyZ0
420	door_gate_Gern	4	video	2	2533,0	1	NA	NA			NA			NA	Pony	2	100	5									https://www.youtube.com/watch?v=G1rqmvbJcrs
421	door_gate_Gern	4	video	2	2534,0	1	NA	NA			g	2		NA	Warmblood	1	350	3	1	single	1	restricted	1	restricted	1	restricted	https://www.youtube.com/watch?v=IIwN-PxdTrw
422	door_gate_Gern	4	video	2	2535,0	1	NA	NA			NA			NA	Pony	2	100	5									https://www.youtube.com/watch?v=3iJMVjydtcM
423	door_gate_Gern	4	video	2	2536,0	1	NA	NA			NA			NA	Warmblood	1	350	3	1	single	1	restricted	1	restricted	1	restricted	https://www.youtube.com/watch?v=F-ZWQt7Htfw
424	door_gate_Gern	4	video	2	2537,0	1	NA	NA			NA			NA	Warmblood	1	350	3									https://www.youtube.com/watch?v=rDMYQbe5Mwk
425	door_gate_Gern	4	video	2	2540,0	1	NA	NA			NA			NA	Warmblood	1	350	3									https://www.youtube.com/watch?v=-nndf7Mhc_o
426	gen.Germ.	2	quest	1	2552,0	4	collect water 3. extinguish fire by stepping on smoldering coal. 4. climbs on bucket	more than 20 times	4		g	2	10,0	unknown	NA				1	single	2	unrestricted	1	restricted	2	unrestricted	
427	gen.Engl.	1	quest	1	2543,0	1		2 - 10 times	2		g	2	23,0	Quater horse	Warmblood	1	350	3	1	single	2	unrestricted	2	unrestricted	2	unrestricted	
428	gen.Engl.	1	quest	1	2550,0	1		more than 20 times	4		g	2	5,0	Quater horse	Warmblood	1	350	3	2	group	2	unrestricted	2	unrestricted	2	unrestricted	
429	door_gate_Germ.	4	quest_door	3	2542,0	2		2 - 10 times	2		g	2	12,0	Islandic horse	Pony	2	100	5	2	group	2	unrestricted	2	unrestricted	1	restricted	

	A	B	C	D	E	G	H	I	J	K	L	M	N	O	P	Q	R	S	T	U	V	W	X	Y	Z	AA	AB	AC	AD
1	sorce	source num	video / quest	video_quest_num	sID	nr reported behaviours	type of other behaviours	how often was the behaviour shown	behaviour frequency	social learning (spreading to other horses)	sex	sex num	age	breed	breed-type	breed type num	length of domestication	purpose of domestication	single group stabling	single group stabling	restricted unrestricted pasture	restricted unrestricted pasture	restricted unrestricted contact with horses	restricted unrestricted contact with horses	restricted unrestricted roughage	restricted unrestricted roughage	link		
430	door_gate_Germ.	4	quest_door	3	2545,0	1		more than 20 times	4		s	3	23,0	Quater mix	Warmblood	1	350	3	2	group	2	unrestricted	2	unrestricted	2	unrestricted			
431	door_gate_Germ.	4	quest_door	3	2545,1	1		more than 20 times	4		g	2		Arabmix	Arabian horse	3	4000	1	2	group	2	unrestricted	2	unrestricted	2	unrestricted			
432	door_gate_Germ.	4	quest_door	3	2551,0	1		more than 20 times	4		m	1	12,0	Haflinger	Warmblood	1	350	3	2	group	2	unrestricted	2	unrestricted	2	unrestricted			
433	gen Germ	2	video	2	2556,0	1	roll snow balls, feed grass underneath								Warmblood	1	350	3									video at: https://m.facebook.com/story.php?story_fbid=10211		
434	gen Germ	2	video	2	2555,0	1	lies underneath fence and eats grass								Warmblood	1	350	3									https://www.youtube.com/watch?v=sJeLi7OqHJQ		
435	door_gate_Germ.	4	quest_door	1	2554,0	1	open sliding door	dayly	5	0	m	1	19,0	Hessian Warmblood	Warmblood	1	350	3	1	single	1	restricted	1	restricted	2	unrestricted			
436																													

Data horse behavior

1	sorce	video / quest	video_ quest_ num	sID	name behaviour	behaviour	nr reported behaviours	how often was the behaviour shown	behaviour frequency	behaviour category num	behaviour _category	feeding behaviour	escape behaviour	social behaviour human	social behaviour horse	social behaviour	comfort behaviour	unknown play	sex	sex num	age	breed	breed-type	bree d type num	length of domestic ation	purpose of domesticati on	purpose of domestica tion	single group stabling	single group stabling	restricted unrestricte d pasture	restricted unrestricted pasture	restricted unrestricted contact with horses	restricted unrestricted contact with horses	restricted unrestric. roughage	restricted unrestricted roughage	links
2	gen.Engl.	quest	1	1958,0	manipulate_human_	horse comes to owner to let him smash a	1	dayly	5	3	social	0	0	1	0	1	0	0	g	2	7,0	Quarter Horse	Warmblood	1	350	3	cooperativ	2	group	2	unrestricted	2	unrestricted	2	unrestrict	
3	gen.Engl.	quest	1	1965,0	open_door_1	open box stall door	5	2-10 times	2	2	escape	0	1	0	0	0	0	0	s	3	1,0	Warmblood	Warmblood	1	350	3	cooperativ	2	group	2	unrestricted	2	unrestricted	2	unrestrict	
4	gen.Engl.	quest	1	1965,0	open_door_1	unty knots	5	2-10 times	2	2	escape	0	1	0	0	0	0	0	s	3	1,0	Warmblood	Warmblood	1	350	3	cooperativ	2	group	2	unrestricted	2	unrestricted	2	unrestrict	
5	gen.Engl.	quest	1	1965,0	open_door_1	bump feed or water bowl against wall	5	2-10 times	2	1	feeding	1	0		0	0	0	0	s	3	1,0	Warmblood	Warmblood	1	350	3	cooperativ	2	group	2	unrestricted	2	unrestricted	2	unrestrict	
6	gen.Engl.	quest	1	1965,0	open_door_1	bump feed or water bowl against wall	5	2-10 times	2	3	social	0	0	1	0	1	0	0	s	3	1,0	Warmblood	Warmblood	1	350	3	cooperativ	2	group	2	unrestricted	2	unrestricted	2	unrestrict	
7	gen.Engl.	quest	1	1965,0	open_door_1	play fetch with feed bowl	5	2-10 times	2	5	play	0	0	0	0	0	0	1	s	3	1,0	Warmblood	Warmblood	1	350	3	cooperativ	2	group	2	unrestricted	2	unrestricted	2	unrestrict	
8	gen.Engl.	quest	1	1978,0	open_box_2	open grooming box to feed on treats	1	dayly	5	1	feeding	1	0	0	0	0	0	0	m	1	18,0	Connemara	Pony	2	100	5	robustnes	2	group	2	unrestricted	2	unrestricted	2	unrestrict	
9	gen.Engl.	quest	1	1980,0	open_door_2	lock opening	4	more than 20 times	4	2	escape	0	1	0	0	0	0	0	s	3	0,5	Lusitano	Warmblood	1	350	3	cooperativ	1	single	2	unrestricted	2	unrestricted	2	unrestrict	
10	gen.Engl.	quest	1	1980,0	untie_itself_2	knot untying	4	more than 20 times	4	2	escape	0	1	0	0	0	0	0	s	3	0,5	Lusitano	Warmblood	1	350	3	cooperativ	2	group	2	unrestricted	2	unrestricted	2	unrestrict	
11	gen.Engl.	quest	1	1980,0	grabbing_bucket_1	asking for more food/water by grabbing	4	more than 20 times	4	1	feeding	1	0	0	0	0	0	0	s	3	0,5	Lusitano	Warmblood	1	350	3	cooperativ	2	group	2	unrestricted	2	unrestricted	2	unrestrict	
12	gen.Engl.	quest	1	1980,0	grabbing_bucket_2	play fetch with grain bucket	4	more than 20 times	4	5	play	0	0	0	0	0	0	1	s	3	0,5	Lusitano	Warmblood	1	350	3	cooperativ	2	group	2	unrestricted	2	unrestricted	2	unrestrict	
13	gen.Engl.	quest	1	1981,0	manipulate_things_1	Esperia ponys turn over stones to reach	1	2-10 times	2	1	feeding	1	0	0	0	0	0	0	m	1		Esperia pony	Warmblood	1	350	3	cooperativ	2	group	2	unrestricted	2	unrestricted	2	unrestrict	
14	gen.Engl.	quest	1	2001,0	manipulate_things_	untying haynets of neighbouring horses	1	2-10 times	2	1	feeding	1	0	0	0	0	0	0	g	2	4,0	Appaloosa	Warmblood	1	350	3	cooperativ	2	group	2	unrestricted	2	unrestricted	1	restricted	
15	gen.Engl.	quest	1	2003,0	open_door_2	opening door	2	dayly	5	2	escape	0	1	0	0	0	0	0	g	2	8,0	Haflinger	Warmblood	1	350	3	cooperativ	1	single	2	unrestricted	2	unrestricted	2	unrestrict	
16	gen.Engl.	quest	1	2003,0	open_ton_1	opening feed tons	2	dayly	5	1	feeding	1	0	0	0	0	0	0	g	2	8,0	Haflinger	Warmblood	1	350	3	cooperativ	1	single	2	unrestricted	2	unrestricted	2	unrestrict	
17	gen.Engl.	quest	1	2004,0	open_door_2	opening door of his box stall	3	11-20 times	3	2	escape	0	1	0	0	0	0	0	g	2	6,0	Shetland	Pony	2	100	5	robustnes	1	single	2	unrestricted	2	unrestricted	1	restricted	
18	gen.Engl.	quest	1	2004,0	open_door_2	opening box stall where food is kept	3	11-20 times	3	1	feeding	1	0	0	0	0	0	0	g	2	6,0	Shetland	Pony	2	100	5	robustnes	1	single	2	unrestricted	2	unrestricted	1	restricted	
19	gen.Engl.	quest	1	2004,0	open_door_2	opening door with handle	3	11-20 times	3	1	feeding	1	0	0	0	0	0	0	g	2	6,0	Shetland	Pony	2	100	5	robustnes	1	single	2	unrestricted	2	unrestricted	1	restricted	
20	gen.Engl.	quest	1	2005,0	open_door_5	open box stall door	1	dayly	5	2	escape	0	1	0	0	0	0	0	g	2	10,0	KWPN	Warmblood	1	350	3	cooperativ	1	single	1	restricted	1	restricted	1	restricted	
21	gen.Engl.	quest	1	2006,0	open_door_6	opening box stall door	1	dayly	5	2	escape	0	1	0	0	0	0	0	g	2	8,0	Swedish	Warmblood	1	350	3	cooperativ	1	single	2	unrestricted	2	unrestricted	1	restricted	
22	gen.Engl.	quest	1	2008,0	open_door_7	open box stall door	1	more than 20 times	4	2	escape	0	1	0	0	0	0	0	m	1	6,0	NA	NA					1	single	1	restricted	1	restricted	1	restricted	
23	gen.Engl.	quest	1	2010,0	open_door_8	opening box stall door, even when secured with rope or carabina	3	dayly	5	2	escape	0	1	0	0	0	0	0	g	2	8,0	Welsh D	Warmblood	1	350	3	cooperativ eness	1	single	2	unrestricted pasture	2	unrestricted contact	1	restricted roughage	
24	gen.Engl.	quest	1	2010,0	untie_itself_3	untie himself	3	dayly	5	2	escape	0	1	0	0	0	0	0	g	2	8,0	Welsh D	Warmblood	1	350	3	cooperativ eness	1	single	2	unrestricted pasture	2	unrestricted contact	1	restricted roughage	
25	gen.Engl.	quest	1	2010,0	fence_2	lift loop of electric wire (without current) off the fence post, move three plastic jumps to get out to grass	3	dayly	5	2	escape	0	1	0	0	0	0	0	g	2	8,0	Welsh D	Warmblood	1	350	3	cooperativ eness	1	single	2	unrestricted pasture	2	unrestricted contact	1	restricted roughage	
26	gen.Engl.	quest	1	2011,0	open_door_9	open box stall door	1	more than 20 times	4	1	feeding	1	0	0	0	0	0	0	m	1	9,0	Friesian horse	Warmblood	1	350	3	cooperativ	1	single	2	unrestricted	2	unrestricted	1	restricted	
27	gen.Engl.	quest	1	2012,0	open_door_10	open box stall door, also with extralock,	1	dayly	5	2	escape	0	1	0	0	0	0	0	g	2	13,0	KWPN	Warmblood	1	350	3	cooperativ	1	single	2	unrestricted	2	unrestricted	2	unrestrict	
28	gen.Engl.	quest	1	2013,0	fence_3	opening electric fence on handle with	1	2-10 times	2	2	escape	0	1	0	0	0	0	0	m	1	4,0	Paint horse	Warmblood	1	350	3	cooperativ	2	group	2	unrestricted	2	unrestricted	2	unrestrict	
29	gen.Engl.	quest	1	2014,0	open_door_11	open box stall door with chain bar that	1	more than 20 times	4	2	escape	0	1	0	0	0	0	0	m	1	6,0	Tinker horse	Draught horse	4	500	4	power	2	group	2	unrestricted	2	unrestricted	2	unrestrict	
30	gen.Engl.	quest	1	2016,0	open_door_12	open box stall door	1	2-10 times	2	2	escape	0	1	0	0	0	0	0	g	2	9,0	KWPN	Warmblood	1	350	3	cooperativ eness	1	single	2	unrestricted pasture	2	unrestricted contact	2	unrestrict roughage	
31	gen.Engl.	quest	1	2017,0	manipulate_human_ 2	pushing owner and looking at thing that is bothering horse when it feels uncomfortable because of a fly or	2	dayly	5	3	social	0	0	1	0	1	0	0	m	1	8,0	Dutch Warmblood	Warmblood	1	350	3	cooperativ eness	1	single	2	unrestricted pasture	2	unrestricted contact	2	unrestrict roughage	
32	gen.Engl.	quest	1	2017,0	manipulate_human_ 3	tapping with hindleg against things in her way, looking at owner to remove it	2	dayly	5	3	social	0	0	1	0	1	0	0	m	1	8,0	Dutch Warmblood	Warmblood	1	350	3	cooperativ eness	1	single	2	unrestricted pasture	2	unrestricted contact	2	unrestrict roughage	
33	gen.Engl.	quest	1	2018,0	open_door_13	open different box stall doors	4	dayly	5	2	escape	0	1	0	0	0	0	0	g	2	14,0	KWPN	Warmblood	1	350	3	cooperativ eness	1	single	1	restricted pasture	1	restricted contact	2	unrestrict roughage	
34	gen.Engl.	quest	1	2018,0	open_door_13	open box stall of neigbouring horse since in new stall	4	dayly	5	3	social	0	0	0	1	1	0	0	g	2	14,0	KWPN	Warmblood	1	350	3	cooperativ eness	1	single	1	restricted pasture	1	restricted contact	2	unrestrict roughage	
35	gen.Engl.	quest	1	2018,0	untie_itself_3	unties rope	4	dayly	5	2	escape	0	1	0	0	0	0	0	g	2	14,0	KWPN	Warmblood	1	350	3	cooperativ eness	1	single	1	restricted pasture	1	restricted contact	2	unrestrict roughage	
36	gen.Engl.	quest	1	2018,0	protection_off_1	take off protection boots	4	dayly	5	4	comfort	0	0	0	0	0	1	0	m	1	4,0	KWPN	Warmblood	1	350	3	cooperativ eness	1	single	1	restricted pasture	1	restricted contact	2	unrestrict roughage	
37	gen.Engl.	quest	1	2019,0	open_door_14	taking out pen of lock, then open closure	2	11-20 times	3	2	escape	0	1	0	0	0	0	0	m	1	10,0	NA	Warmblood	1	350	3	cooperativ	2	group	2	unrestricted	2	unrestricted	1	restricted	
38	gen.Engl.	quest	1	2019,0	fence_3	going through wire the second the power	3	11-20 times	3	2	escape	0	1	0	0	0	0	0	m	1	10,0	NA	Warmblood	1	350	3	cooperativ	2	group	2	unrestricted	2	unrestricted	1	restricted	
39	gen.Engl.	quest	1	2020,0	manipulate_human_	horse goes to the rung if it does not want	1	dayly	5	3	social	0	0	1	0	1	0	0	m	1	15,0	Haflinger	Warmblood	1	350	3	cooperativ	1	single	2	unrestricted	2	unrestricted	1	restricted	
40	gen.Engl.	quest	1	2021,0	fence_5	pulling sticks of electric fencing out of	3	2-10 times	2	2	escape	0	1	0	0	0	0	0	m	1	6,0	Appaloosa	Warmblood	1	350	3	cooperativ	2	group	2	unrestricted	2	unrestricted	2	unrestrict	
41	gen.Engl.	quest	1	2021,0	fence_5	pulling sticks of electric fencing out of	3	2-10 times	2	5	play	0	0	0	0	0	0	1	m	1	6,0	Appaloosa	Warmblood	1	350	3	cooperativ	2	group	2	unrestricted	2	unrestricted	2	unrestrict	
42	gen.Engl.	quest	1	2021,0	open_door_15	escaping from different stables with	3	2-10 times	2	2	escape	0	1	0	0	0	0	0	m	1	6,0	Appaloosa	Warmblood	1	350	3	cooperativ	2	group	2	unrestricted	2	unrestricted	2	unrestrict	
43	gen.Engl.	quest	1	2022,0	open_door_16	open handles on stables	2	more than 20 times	4	1	feeding	1	0	0	0	0	0	0	g	2	25,0	Connemara	Pony	2	100	5	robustnes	2	group	2	unrestricted	2	unrestricted	2	unrestrict	
44	gen.Engl.	quest	1	2022,0	unty_itself	open ropes	2	more than 20 times	4	2	escape	0	1	0	0	0	0	0	g	2	25,0	Connemara	Pony	2	100	5	robustnes	2	group	2	unrestricted	2	unrestricted	2	unrestrict	
45	gen.Engl.	quest	1	2030,0	tool_use_3	scratching himself abdomen/belly with a	1	2-10 times	2	4	comfort	0	0	0	0	0	1	0	g	2	14,0	Arabian	Arabian horse	3	4000	1	endurance	2	group	2	unrestricted	2	unrestricted	2	unrestrict	
46	gen.Engl.	quest	1	2031,0	open_door_17	opening different gates and latches	12	more than 20 times	4	2	escape	0	1	0	0	0	0	0	g	2	15,0	Missouri Fox	Warmblood	1	350	3	cooperativ	1	single	2	unrestricted	1	restricted	2	unrestrict	
47	gen.Engl.	quest	1	2031,0	open_door_17	opening door handle	12	more than 20 times	4	1	feeding	1	0	0	0	0	0	0	g	2	15,0	Missouri Fox	Warmblood	1	350	3	cooperativ	1	single	2	unrestricted	1	restricted	2	unrestrict	
48	gen.Engl.	quest	1	2031,0	tool_use_4	picking up whip an hitting other horses	12	more than 20 times	4	3	social	0	0	0	1	1	0	0	g	2	15,0	Missouri Fox	Warmblood	1	350	3	cooperativ	1	single	2	unrestricted	1	restricted	1	restricted	
49	gen.Engl.	quest	1	2031,0	action_1	opening horse trailer and trying to load	12	more than 20 times	4	5	play	0	0	0	0	0	0	1	g	2	15,0	Missouri Fox	Warmblood	1	350	3	cooperativ	1	single	2	unrestricted	1	restricted	1	restricted	
50	gen.Engl.	quest	1	2031,0	comfort_3	walk with halter clip in his mouth so it	12	more than 20 times	4	4	comfort	0	0	0	0	0	1	0	g	2	15,0	Missouri Fox	Warmblood	1	350	3	cooperativ	1	single	2	unrestricted	1	restricted	1	restricted	
51	[illegible]	[illegible]	1	2031,0	[illegible]	[illegible]	12	[illegible]	4	[illegible]	[illegible]	0	1	0	0	0	0	0	[illegible]	2	15,0	[illegible]	[illegible]	1	350	3	[illegible]	1	[illegible]	2	[illegible]	1	[illegible]	1	[illegible]	

1	sorce	video / quest	video_quest_num	sID	name behaviour	behaviour	nr reported behaviours	how often was the behaviour shown	behaviour frequency	behaviour category num	behaviour_category	feeding behaviour	escape behaviour	social behaviour human	social behaviour horse	social behaviour	comfort behaviour	unknown play	sex	sex num	age	breed	breed-type	breed type num	length of domestication	purpose of domestication	purpose of domestication	single group stabling	single group stabling	restricted unrestricted pasture	restricted unrestricted pasture	restricted unrestricted contact with horses	restricted unrestricted contact with horses	restricted unrestric. roughage	restricted unrestricted roughage	links
51	gen.Engl.	quest	1	2031,0	untie_itself_4	untie himself	12	more than 20 times	4	2	escape	0	1	0	0	0	0	0	g	2	15,0	Missouri Fox	Warmblood	1	350	3	cooperativ	1	single	2	unrestricted	1	restricted	1	restricted	
52	gen.Engl.	quest	1	2031,0	untie_itself_5	untie himself with blocker tie ring by	12	more than 20 times	4	2	escape	0	1	0	0	0	0	0	g	2	15,0	Missouri Fox	Warmblood	1	350	3	cooperativ	1	single	2	unrestricted	1	restricted	1	restricted	
53	gen.Engl.	quest	1	2031,0	protection_off_2	taking off tendon boots by opening the	12	more than 20 times	4	4	comfort	0	0	0	0	0	1	0	g	2	15,0	Missouri Fox	Warmblood	1	350	3	cooperativ	1	single	2	unrestricted	1	restricted	1	restricted	
54	gen.Engl.	quest	1	2031,0	protection_off_3	taking tail wraps off, lying down for it	12	more than 20 times	4	4	comfort	0	0	0	0	0	1	0	g	2	15,0	Missouri Fox	Warmblood	1	350	3	cooperativ	1	single	2	unrestricted	1	restricted	1	restricted	
55	gen.Engl.	quest	1	2031,0	protection_off_5	trying to scrape hind boots off on tire	12	more than 20 times	4	4	comfort	0	0	0	0	0	1	0	g	2	15,0	Missouri Fox	Warmblood	1	350	3	cooperativ	1	single	2	unrestricted	1	restricted	1	restricted	
56	gen.Engl.	quest	1	2031,0	manipulate_things_5	shaking jingle bell after seeing boy doing	12	more than 20 times	4	5	play	0	0	0	0	0	0	1	g	2	15,0	Missouri Fox	Warmblood	1	350	3	cooperativ	1	single	2	unrestricted	1	restricted	1	restricted	
57	gen.Engl.	quest	1	2031,0	protection_off_6	taking fly mask off and leave it outside the	12	more than 20 times	4	4	comfort	0	0	0	0	0	1	0	g	2	15,0	Missouri Fox	Warmblood	1	350	3	cooperativ	1	single	2	unrestricted	1	restricted	1	restricted	
58	gen.Engl.	quest	1	2032,0	borderline_tool_use	pulling chair with bag of carrots closer	5	2-10 times	2	1	feeding	1	0	0	0	0	0	0	g	2	8,0	Iceland horse	Pony	2	100	5	robustnes	1	single	2	unrestricted	1	restricted	1	restricted	
59	gen.Engl.	quest	1	2032,0	open_box_3	opening box containing hose reel	5	2-10 times	2	1	feeding	1	0	0	0	0	0	0	g	2	8,0	Iceland horse	Pony	2	100	5	robustnes	1	single	2	unrestricted	1	restricted	1	restricted	
60	gen.Engl.	quest	1	2032,0	protection_off_7	taking off different muzzles by scratching	5	2-10 times	2	4	comfort	0	0	0	0	0	1	0	g	2	8,0	Iceland horse	Pony	2	100	5	robustnes	1	single	2	unrestricted	1	restricted	1	restricted	
61	gen.Engl.	quest	1	2032,0	untie_itself_6	untying himself	5	2-10 times	2	2	escape	0	1	0	0	0	0	0	g	2	8,0	Iceland horse	Pony	2	100	5	robustnes	1	single	2	unrestricted	1	restricted	1	restricted	
62	gen.Engl.	quest	1	2032,0	intellegence_tests_1	solving different "horse intelligence tests"	5	2-10 times	2	1	feeding	1	0	0	0	0	0	0	g	2	8,0	Iceland horse	Pony	2	100	5	robustnes	1	single	2	unrestricted	1	restricted	1	restricted	
63	gen.Engl.	quest	1	2033,0	comfort_7	taking off briddle	4	2-10 times	2	4	comfort	0	0	0	0	0	1	0	g	2	9,0	Friesian horse	Warmblood	1	350	3	cooperativ	1	single	1	restricted	1	restricted	1	restricted	
64	gen.Engl.	quest	1	2033,0	open_door_18	unlock gate of round pen	4	2-10 times	2	2	escape	0	1	0	0	0	0	0	g	2	9,0	Friesian horse	Warmblood	1	350	3	cooperativ	1	single	1	restricted	1	restricted	1	restricted	
65	gen.Engl.	quest	1	2033,0	open_door_19	after an additional lock was put on the	4	2-10 times	2	2	escape	0	1	0	0	0	0	0	g	2	9,0	Friesian horse	Warmblood	1	350	3	cooperativ	1	single	1	restricted	1	restricted	1	restricted	
66	gen.Engl.	quest	1	2033,0	open_door_20	lifting the chain up off the peg of a gate	4	2-10 times	2	2	escape	0	1	0	0	0	0	0	g	2	9,0	Friesian horse	Warmblood	1	350	3	cooperativ	1	single	1	restricted	1	restricted	1	restricted	
67	gen.Engl.	quest	1	2034,0	untie_itself_7	slipping halter off	5	more than 20 times	4	2	escape	0	1	0	0	0	0	0	g	2	3,0	Palomino	Warmblood	1	350	3	cooperativ	2	group	2	unrestricted	2	unrestricted	2	unrestrict	
68	gen.Engl.	quest	1	2034,0	untie_others	untying other horses	5	more than 20 times	4	3	social	0	0	0	1	1	0	0	g	2	3,0	Palomino	Warmblood	1	350	3	cooperativ	2	group	2	unrestricted	2	unrestricted	2	unrestrict	
69	gen.Engl.	quest	1	2034,0	tool_use_5	herding other horse by carrying stick in	5	more than 20 times	4	3	social	0	0	0	1	1	0	0	g	2	3,0	Palomino	Warmblood	1	350	3	cooperativ	2	group	2	unrestricted	2	unrestricted	2	unrestrict	
70	gen.Engl.	quest	1	2034,0	grabbing_bucket_3	yanking own food dish out of tire to get	5	more than 20 times	4	1	feeding	1	0	0	0	0	0	0	g	2	3,0	Palomino	Warmblood	1	350	3	cooperativ	2	group	2	unrestricted	2	unrestricted	2	unrestrict	
71	gen.Engl.	quest	1	2034,0	grabbing_bucket_4	yanking still partially filled food dish of	5	more than 20 times	4	1	feeding	1	0	0	0	0	0	0	g	2	3,0	Palomino	Warmblood	1	350	3	cooperativ	2	group	2	unrestricted	2	unrestricted	2	unrestrict	
72	gen.Engl.	quest	1	2036,0	open_door_21	opening doors and latches even when	1	more than 20 times	4	2	escape	0	1	0	0	0	0	0	m	1	12,0	Finhorse	Warmblood	1	350	3	cooperativ	2	group	2	unrestricted	2	unrestricted	1	restricted	
73	gen.Engl.	quest	1	2038,0	borderline_tool_use	stepping on edge of the tub to turn it	1	more than 20 times	4	4	comfort	0	0	0	0	0	1	0	m	1	19,0	Belgian horse	Draught horse	4	500	4	power	2	group	2	unrestricted	2	unrestricted	1	restricted	
74	gen.Engl.	quest	1	2039,0	open_door_22	open door with bolt of own stable	2	more than 20 times	4	2	escape	0	1	0	0	0	0	0	g	2	7,0	Quarter horse	Warmblood	1	350	3	cooperativ	1	single	2	unrestricted	2	unrestricted	2	unrestrict	
75	gen.Engl.	quest	1	2039,0	open_door_22	open door with bolt other horses' stables	2	more than 20 times	4	3	social	0	0	0	1	1	0	0	g	2	7,0	Quarter horse	Warmblood	1	350	3	cooperativ	1	single	2	unrestricted	2	unrestricted	2	unrestrict	
76	gen.Engl.	quest	1	2040,0	open_door_23	opening doors with handle and doors	2	more than 20 times	4	2	escape	0	1	0	0	0	0	0	m	1	7,0	Arabian	Arabian horse	3	4000	1	endurance	1	single	1	restricted	1	restricted	1	restricted	
77	gen.Engl.	quest	1	2040,0	open_door_23	opening doors with handle and doors	2	more than 20 times	4	3	social	0	0	0	1	1	0	0	m	1	7,0	Arabian	Arabian horse	3	4000	1	endurance	1	single	1	restricted	1	restricted	1	restricted	
78	gen.Engl.	quest	1	2042,0	protection_off_8	taking off boots of horse in the	1	2-10 times	2	5	play	0	0	0	0	0	0	1	g	2	3,0	Arabian	Arabian horse	3	4000	1	endurance	1	single	1	restricted	1	restricted	1	restricted	
79	gen.Engl.	quest	1	2044,0	untie_itself_8	taking off halter	5	dayly	5	2	escape	0	1	0	0	0	0	0	g	2	2,0	Quarter horse	Warmblood	1	350	3	cooperativ	2	group	2	unrestricted	2	unrestricted	1	restricted	
80	gen.Engl.	quest	1	2044,0	untie_itself_9	untying rope	5	dayly	5	2	escape	0	1	0	0	0	0	0	g	2	2,0	Quarter horse	Warmblood	1	350	3	cooperativ	2	group	2	unrestricted	2	unrestricted	1	restricted	
81	gen.Engl.	quest	1	2044,0	open_door_24	open gates	5	dayly	5	2	escape	0	1	0	0	0	0	0	g	2	2,0	Quarter horse	Warmblood	1	350	3	cooperativ	2	group	2	unrestricted	2	unrestricted	1	restricted	
82	gen.Engl.	quest	1	2044,0	borderline_tool_use	stepping in feed tub to keep grain ration	5	dayly	5	1	feeding	1	0	0	0	0	0	0	g	2	2,0	Quarter horse	Warmblood	1	350	3	cooperativ	2	group	2	unrestricted	2	unrestricted	1	restricted	
83	gen.Engl.	quest	1	2044,0	protection_off_9	taking off blanket (with buckles still	5	dayly	5	4	comfort	0	0	0	0	0	1	0	g	2	2,0	Quarter horse	Warmblood	1	350	3	cooperativ	2	group	2	unrestricted	2	unrestricted	1	restricted	
84	gen.Engl.	quest	1	2046,0	untie_itself_10	untie herself	3	more than 20 times	4	2	escape	0	1	0	0	0	0	0	m	1	13,0	Missouri Fox	Warmblood	1	350	3	cooperativ	2	group	2	unrestricted	2	unrestricted	1	restricted	
85	gen.Engl.	quest	1	2046,0	open_door_25	open tack room door	3	more than 20 times	4	1	feeding	1	0	0	0	0	0	0	m	1	13,0	Missouri Fox	Warmblood	1	350	3	cooperativ	2	group	2	unrestricted	2	unrestricted	1	restricted	
86	gen.Engl.	quest	1	2046,0	open_box_5	open box with cookies and dropping it,	3	more than 20 times	4	1	feeding	1	0	0	0	0	0	0	m	1	13,0	Missouri Fox	Warmblood	1	350	3	cooperativ	2	group	2	unrestricted	2	unrestricted	1	restricted	
87	gen.Engl.	quest	1	2051,0	watering_hay_1	moving hay to the other side of the box to	1	more than 20 times	4	1	feeding	1	0	0	0	0	0	0	g	2	21,0	Irish hunter	Warmblood	1	350	3	cooperativ	1	single	2	unrestricted	1	restricted	1	restricted	
88	gen.Engl.	quest	1	2062,0	open_door_26	opening bolt of box stall door and going	2	more than 20 times	4	2	escape	0	1	0	0	0	0	0	g	2	10,0	Anglo-Arab	Arabian horse	3	4000	1	endurance	1	single	2	unrestricted	2	unrestricted	2	unrestrict	
89	gen.Engl.	quest	1	2062,0	open_door_26	opening bolt of box stall door letting	2	more than 20 times	4	3	social	0	0	0	1	1	0	0	g	2	10,0	Anglo-Arab	Arabian horse	3	4000	1	endurance	1	single	2	unrestricted	2	unrestricted	2	unrestrict	
90	gen.Engl.	quest	1	2063,0	open_door_27	opening door when being ridden after	1	more than 20 times	4	2	escape	0	1	0	0	0	0	0	g	2	5,0	Anglo-Arab	Arabian horse	3	4000	1	endurance	1	single	2	unrestricted	2	unrestricted	2	unrestrict	
91	gen.Engl.	quest	1	2064,0	open_door_28	open stall to let himself out, then search	4	more than 20 times	4	2	escape	0	1	0	0	0	0	0	g	2	12,0	Morgan	Warmblood	1	350	3	cooperativ	1	single	2	unrestricted	2	unrestricted	2	unrestrict	
92	gen.Engl.	quest	1	2064,0	open_door_28	open stall to let other horses out, then	4	more than 20 times	4	3	social	0	0	0	1	1	0	0	g	2	12,0	Morgan	Warmblood	1	350	3	cooperativ	1	single	2	unrestricted	2	unrestricted	2	unrestrict	
93	gen.Engl.	quest	1	2064,0	open_ton_2	opening bins	4	more than 20 times	4	1	feeding	1	0	0	0	0	0	0	g	2	12,0	Morgan	Warmblood	1	350	3	cooperativ	1	single	2	unrestricted	2	unrestricted	2	unrestrict	
94	gen.Engl.	quest	1	2064,0	open_door_29	open pasture gate to let himself and other	4	more than 20 times	4	2	escape	0	1	0	0	0	0	0	g	2	12,0	Morgan	Warmblood	1	350	3	cooperativ	1	single	2	unrestricted	2	unrestricted	2	unrestrict	
95	gen.Engl.	quest	1	2066,0	comfort_4	digging hole, causing the waterer to<br>overflow into hole and then rolling in the	1	11-20 times	3	4	comfort	0	0	0	0	0	1	0	g	2	20,0	NA	NA					1	single	1	restricted pasture	1	restricted	1	restricted roughage	
96	gen.Engl.	quest	1	1947,0	harvesting_apples_1	bucking and kicking against apple tree so t	1	dayly	5	1	feeding	1	0	0	0	0	0	0	m	1	3,0	NA	Warmblood	1	350	3	cooperativ	2	group	2	unrestricted	2	unrestricted	1	restricted	
97	gen.Engl.	quest	1	1952,0	open_door_30	opening doors	2	2-10 times	2	2	escape	0	1	0	0	0	0	0	m	1	2,0	Friesian horse	Warmblood	1	350	3	cooperativ	2	group	2	unrestricted	2	unrestricted	2	unrestrict	
98	gen.Engl.	quest	1	1952,0	open_box_6	opening freezer	2	2-10 times	2	1	feeding	1	0	0	0	0	0	0	m	1	2,0	Friesian horse	Warmblood	1	350	3	cooperativ	2	group	2	unrestricted	2	unrestricted	2	unrestrict	
99	gen.Engl.	quest	1	1957,0	fence_8	opening the electric fence through	1	dayly	5	2	escape	0	1	0	0	0	0	0	s	3	12,0	Selle Francais	Warmblood	1	350	3	cooperativ	2	group	2	unrestricted	2	unrestricted	2	unrestrict	
100	gen.Engl.	quest	1	1960,0	manipulate_things_1	picking up things, taking with him and	3	2-10 times	2	5	play	0	0	0	0	0	0	1	g	2	7,0	Paint horse	Warmblood	1	350	3	cooperativ	1	single	2	unrestricted	2	unrestricted	1	restricted	
101	gen.Engl.	quest	1	1960,0	untie_itself_11	opening knots	3	2-10 times	2	2	escape	0	1	0	0	0	0	0	g	2	7,0	Paint horse	Warmblood	1	350	3	cooperativ	1	single	2	unrestricted	2	unrestricted	1	restricted	
102	gen.Engl.	quest	1	1960,0	open_door_31	opening doors (also of other horses	3	dayly	5	2	escape	0	1	0	0	0	0	0	g	2	7,0	Paint horse	Warmblood	1	350	3	cooperativ	1	single	2	unrestricted	2	unrestricted	1	restricted	
103	gen.Engl.	quest	1	1961,0	automatic_feeder_1	Kicking wall so that grains fall out of the	5	dayly	5	1	feeding	1	0	0	0	0	0	0	g	2	9,0	Hispano	Arabian horse	3	4000	1	endurance	2	group	2	unrestricted	2	unrestricted	1	restricted	
104	gen.Engl.	quest	1	1961,0	automatic_feeder_2	Pulling out hay of the automatic hay feeder	5	dayly	5	1	feeding	1	0	0	0	0	0	0	g	2	9,0	Hispano	Arabian horse	3	4000	1	endurance	2	group	2	unrestricted	2	unrestricted	1	restricted	
105	gen.Engl.	quest	1	1961,0	untie_itself_12	opening knots	5	dayly	5	2	escape	0	1	0	0	0	0	0	g	2	9,0	Hispano	Arabian horse	3	4000	1	endurance	2	group	2	unrestricted	2	unrestricted	1	restricted	
106	gen.Engl.	quest	1	1961,0	manipulate_things_1	opening belts with mouth	5	dayly	5	5	play	0	0	0	0	0	0	1	g	2	9,0	Hispano	Arabian horse	3	4000	1	endurance	2	group	2	unrestricted	2	unrestricted	1	restricted	
107	gen.Engl.	quest	1	1961,0	open_door_31	opening doors	5	dayly	5	2	escape	0	1	0	0	0	0	0	g	2	9,0	Hispano	Arabian horse	3	4000	1	endurance	2	group	2	unrestricted	2	unrestricted	1	restricted	
108	gen.Germ.	quest	1	1963,0	open_door_32	opens door to feed room and feeds there	1	dayly	5	1	feeding	1	0	0	0	0	0	0	m	1	14,0	German	Pony	2	100	5	robustnes	1	single	1	restricted	1	restricted	2	unrestrict	
109	gen.Engl.	quest	1	1964,0	open_door_33	opening different doors	2	dayly	5	2	escape	0	1	0	0	0	0	0	g	2	10,0	Trotter Mix	Thoroughbre	5	250	2	speed	2	group	2	unrestricted	2	unrestricted	1	restricted	
110	gen.Engl.	quest	1	1964,0	untie_itself_13	opening safety-knot	2	dayly	5	2	escape	0	1	0	0	0	0	0	g	2	10,0	Trotter Mix	Thoroughbre	5	250	2	speed	2	group	2	unrestricted	2	unrestricted	1	restricted	
111	[illegible]	[illegible]	1	[illegible]	[illegible]	[illegible]	1	[illegible]	5	1	[illegible]	1	0	0	0	0	0	0	m	1	[illegible]	[illegible]	[illegible]	2	100	5	[illegible]	1	[illegible]	2	[illegible]	2	[illegible]	1	[illegible]	

1	sorce	video / quest	video_quest_num	sID	name behaviour	behaviour	nr reported behaviours	how often was the behaviour shown	behaviour frequency	behaviour category num	behaviour _category	feeding behaviour	escape behaviour	social behaviour human	social behaviour horse	social behaviour	comfort behaviour	unknown play	sex	sex num	age	breed	breed-type	breed type num	length of domestication	purpose of domestication	purpose of domestication	single group stabling	single group stabling	restricted unrestricted pasture	restricted unrestricted pasture	restricted unrestricted contact with horses	restricted unrestricted contact with horses	restricted unrestric. roughage	restricted unrestricted roughage	links
111	gen.Engl.	quest	1	1969,0	open_ton_3	opening barrel with screw cap by	1	dayly	5	1	feeding	1	0	0	0	0	0	0	m	1	31,0	Mini Shetland	Pony	2	100	5	robustnes	1	single	2	unrestricted	2	unrestricted	1	restricted	
112	gen.Germ.	quest	1	1969,1	open door	breaking in the feed room	1	dayly	5	1	feeding	1	0	0	0	0	0	0	m	1	13,0	NA	Warmblood	1	350	3	cooperativ	1	single	2	unrestricted	2	unrestricted	1	restricted	
113	gen.Engl.	quest	1	1970,0	open_door_34	opening door with two locks	2	dayly	5	2	escape	0	1	0	0	0	0	0	s	3	4,0	PRE	Warmblood	1	350	3	cooperativ	1	single	2	unrestricted	2	unrestricted	1	restricted	
114	gen.Engl.	quest	1	1970,0	manipulate_human_5	if the horse thinks he has worked enough	2	dayly	5	3	social	0	0	1	0	1	0	0	g	2	6,0	PRE	Warmblood	1	350	3	cooperativ	1	single	2	unrestricted	2	unrestricted	1	restricted	
115	gen.Engl.	quest	1	1971,0	manipulate_things_15	pulling hay bags (Heusäcke) into box stall by pulling it along the grates and lifting it into the box	2	more than 20 times	4	1	feeding	1	0	0	0	0	0	0	g	2	5,0	Pinto	Warmblood	1	350	3	cooperativeness	1	single	2	unrestricted pasture	1	restricted	1	restricted roughage	
116	gen.Germ.	quest	1	1971,0	open door - gate	open door - gate	2	more than 20 times	4	2	escape	0	1	0	0	0	0	0	g	2	6,0	Pinto	Warmblood	1	350	3	cooperativeness	1	single	2	unrestricted pasture	1	restricted	1	restricted roughage	
117	gen.Engl.	quest	1	1972,0	open_ton_4	opening barrel?/Tonne with seitlichem	2	more than 20 times	4	1	feeding	1	0	0	0	0	0	0	g	2	13,0	Fjordhorse	Warmblood	1	350	3	cooperativ	1	single	2	unrestricted	1	restricted	2	unrestrict	
118	gen.Engl.	quest	1	1972,0	open_box_7	opening cupboard with upper lip on the	2	2-10 times	2	1	feeding	1	0	0	0	0	0	0	g	2	13,0	Fjordhorse	Warmblood	1	350	3	cooperativ	1	single	2	unrestricted	1	restricted	2	unrestrict	
119	gen.Germ.	quest	1	1972,1	open door - gate	open door - gate	2	2-10 times	2	2	escape	0	1	0	0	0	0	0	s	3	30,0	NA	Pony	2	100	5	robustnes	1	single	2	unrestricted	1	restricted	2	unrestrict	
120	gen.Engl.	quest	1	1972,1	opne_box	open feed container	2	2-10 times	2	1	feeding	1	0	0	0	0	0	0	s	3	30,0	NA	Pony	2	100	5	robustnes	1	single	2	unrestricted	1	restricted	2	unrestrict	
121	gen.Engl.	quest	1	1977,0	untie_itself_10	taking off the halter	2	dayly	5	2	escape	0	1	0	0	0	0	0	g	2	3,0	Paint horse	Warmblood	1	350	3	cooperativ	2	group	2	unrestricted	2	unrestricted	1	restricted	
122	gen.Germ.	quest	1	1977,0	open door - gate	open door - gate	2	dayly	5	2	escape	0	1	0	0	0	0	0	g	2	3,0	Paint horse	Warmblood	1	350	3	cooperativ	2	group	2	unrestricted	2	unrestricted	1	restricted	
123	gen.Engl.	quest	1	1982,0	untie_itself_11	opening knot	1	11-20 times	3	2	escape	0	1	0	0	0	0	0	s	3	24,0	NA	Pony	2	100	5	robustnes	2	group	2	unrestricted	2	unrestricted	2	unrestrict	
124	gen.Germ.	quest	1	1982,1	open door - gate	open door - gate	1	11-20 times	3	2	escape	0	1	0	0	0	0	0	s	3	24,0	NA	Pony	2	100	5	robustnes	2	group	2	unrestricted	2	unrestricted	2	unrestrict	
125	gen.Engl.	quest	1	1983,0	open_door_35	opens different doors with different locks	1	more than 20 times	4	2	escape	0	1	0	0	0	0	0	g	2	14,0	Württemberg	Warmblood	1	350	3	cooperativ	1	single	2	unrestricted	2	unrestricted	1	restricted	
126	gen.Engl.	quest	1	1984,0	manipulate things	kick against feeder so that food falls out	4	dayly	5	1	feeding	1	0	0	0	0	0	0	g	2	9,0	Arab mix	Arabian horse	3	4000	1	endurance	2	group	2	unrestricted	2	unrestricted	2	unrestrict	
127	gen.Engl.	quest	1	1984,0	manipuilate things	stay in door of feedr so that it can not	4	dayly	5	1	feeding	1	0	0	0	0	0	0	g	2	9,0	Arab mix	Arabian horse	3	4000	1	endurance	2	group	2	unrestricted	2	unrestricted	2	unrestrict	
128	gen.Engl.	quest	1	1984,0	unty itself	open knots	4	dayly	5	2	escape	0	1	0	0	0	0	0	g	2	9,0	Arab mix	Arabian horse	3	4000	1	endurance	2	group	2	unrestricted	2	unrestricted	2	unrestrict	
129	gen.Germ.	quest	1	1984,0	open door	open door	4	NA		1	feeding	1	0	0	0	0	0	0	g	2	9,0	Arab mix	Arabian horse	3	4000	1	endurance	2	group	2	unrestricted	2	unrestricted	1	restricted	
130	gen.Engl.	quest	1	1961,1	stepping_on_things	Climbing on the hay rack to have free	1	NA		1	feeding	1	0	0	0	0	0	0	m	1	14,0	Haflinger	Warmblood	1	350	3	cooperativ	2	group	2	unrestricted	2	unrestricted	1	restricted	
131	gen.Germ.	quest	1	1986,0	open door - gate	open door - gate	3	dayly	5	2	escape	0	1	0	0	0	0	0	g	2	8,0	Pony mix	Pony	2	100	5	robustnes	1	single	2	unrestricted	2	unrestricted	1	restricted	
132	gen.Germ.	quest	1	1986,0	untie_itself	unty knots	3	dayly	5	2	escape	0	1	0	0	0	0	0	g	2	8,0	Pony mix	Pony	2	100	5	robustnes	1	single	2	unrestricted	2	unrestricted	1	restricted	
133	gen.Germ.	quest	1	1986,0	manipulating_things	raking with stick	3	dayly	5	5	play	0	0	0	0	0	0	1	g	2	8,0	Pony mix	Pony	2	100	5	robustnes	1	single	2	unrestricted	2	unrestricted	1	restricted	
134	gen.Germ.	quest	1	1988,0	open fence	opens electric fence handles	1	NA		2	escape	0	1	0	0	0	0	0	g	2	12,0	Selle Francais	Warmblood	1	350	3	cooperativ									
135	gen.Engl.	quest	1	1989,0	tool_use_6	Using a stick to scratch out hay from	1	NA		1	feeding	1	0	0	0	0	0	0	g	2	18,0	Freiberger	Warmblood	1	350	3	cooperativ	2	group							
136	gen.Engl.	quest	1	1990,0	manipulate_horse_2	flinging/dashing? horses against electric	1	dayly	5	3	social	0	0	0	1	1	0	0	g	2		Friesian horse	Warmblood	1	350	3	cooperativ	2	group							
137	gen.Germ.	quest	1	1990,1	open door - gate	open door - gate, own door , stall door an	4	dayly	5	2	escape	0	1	0	0	0	0	0	m	1	3,0	NA	Warmblood	1	350	3	cooperativ	1	single	1	restricted	2	unrestricted	1	restricted	facebook
138	gen.Germ.	quest	1	1990,1	open door - gate	open door - of other horses boxes	4	dayly	5	3	social	0	0	0	1	1	0	0	m	1	3,0	NA	Warmblood	1	350	3	cooperativ	1	single	1	restricted	2	unrestricted	1	restricted	facebook
139	gen.Germ.	quest	1	1990,1	open door - gate	open door - feed room	4	dayly	5	1	feeding	1	0	0	0	0	0	0	m	1	3,0	NA	Warmblood	1	350	3	cooperativ	1	single	1	restricted	2	unrestricted	1	restricted	facebook
140	gen.Germ.	quest	1	1990,1	manipulate_things	open feed box	4	dayly	5	1	feeding	1	0	0	0	0	0	0	m	1	3,0	NA	Warmblood	1	350	3	cooperativ	1	single	1	restricted	2	unrestricted	1	restricted	facebook
141	gen.Engl.	quest	1	1993,0	feeding_station_1	Pulling open door to feeding	1	NA		1	feeding	1	0	0	0	0	0	0	g	2	3,0	Friesian horse	Warmblood	1	350	3	cooperativ	2	group							
142	gen.Engl.	quest	1	1994,0	feeding_station_2	Eating feed of others in concentrate and	1	dayly	5	1	feeding	1	0	0	0	0	0	0	g	2	21,0	Camargue	Warmblood	1	350	3	cooperativ	2	group	2	unrestricted			1	restricted	
143	gen.Engl.	video	3	1998,0	feeding_horse_1	feeding other horse (in box with saw dust)	1	NA		3	social	0	0	0	1	1	0	0	NA			NA	Warmblood	1	350	3	cooperativ									
144	gen.Engl.	video	3	1999,0	untie_itself_12	taking off the halter by passing rope over	1	NA		2	escape	0	1	0	0	0	0	0	g	2		NA	Warmblood	1	350	3	cooperativ									
145	gen.Germ.	quest	1	2000,0	open door - gate	open door - gate	2	more than 20 times	4	2	escape	0	1	0	0	0	0	0	g	2	19,0	NA	Warmblood	1	350	3	cooperativ	1	single	2	unrestricted	2	unrestricted			
146	gen.Germ.	quest	1	2000,0	manipulate_things	takes down electric fence	2	more than 20 times	4	2	escape	0	1	0	0	0	0	0	g	2	19,0	NA	Warmblood	1	350	3	cooperativ	1	single	2	unrestricted	2	unrestricted			
147	gen.Engl.	quest	1	2023,0	open_door_37	opening box stall doors	2	more than 20 times	4	2	escape	0	1	0	0	0	0	0	g	2	19,0	Oldenburg	Warmblood	1	350	3	cooperativ	1	single	2	unrestricted	2	unrestricted	1	restricted	
148	gen.Engl.	quest	1	2023,0	fence_12	pulling out the fence posts of the	2	more than 20 times	4	2	escape	0	1	0	0	0	0	0	g	2	19,0	Oldenburg	Warmblood	1	350	3	cooperativ	1	single	2	unrestricted	2	unrestricted	1	restricted	
149	gen.Engl.	video	3	2037,0	tool_use_1	foal grooms mother with curry comb	1	NA		3	social	0	0	0	1	1	0	0	NA		0,5	NA	Warmblood	1	350	3	cooperativ	1	single							
150	gen.Engl.	video	3	2056,0	open_box_8	open grooming box to feed on carrot	1	NA		1	feeding	1	0	0	0	0	0	0	g	2		NA	Warmblood	1	350	3	cooperativ									
151	gen.Engl.	video	3	2057,0	harvesting_apples_	bucking and kicking against apple tree so	1	NA		1	feeding	1	0	0	0	0	0	0	g	2		NA	Warmblood	1	350	3	cooperativ									
152	gen.Engl.	quest	1	2061,0	manipulate_things_1	turning over feed tub to get the rest of the	1	more than 20 times	4	1	feeding	1	0	0	0	0	0	0	g	2	3,0	Iceland horse	Pony	2	100	5	robustnes	2	group	2	unrestricted	1	restricted			
153	gen.Germ.	quest	1	2069,0	open_door_38	opening bolts/doors	2	dayly	5	2	escape	0	1	0	0	0	0	0	g	2	4,0	Trakehner	Warmblood	1	350	3	cooperativ	1	single	1	restricted	1	restricted	1	restricted	
154	gen.Germ.	quest	1	2069,0	open_door_38	unty knots	2	dayly	5	2	escape	0	1	0	0	0	0	0	g	2	4,0	Trakehner	Warmblood	1	350	3	cooperativ	1	single	1	restricted	1	restricted	1	restricted	
155	gen.Germ.	video	3	2071,0	open_door_40	opening box stall door	1	NA		2	escape	0	1	0	0	0	0	0	NA			NA	Warmblood	1	350	3	cooperativ	1	single							Ein schlaues Pferd - YouTube [720p].
156	gen.Engl.	video	3	2074,0	open_water_hoase_a_drink	pulls water hoase towards fence, opens hoase by turning lever, drinking water , closing hose by turning lever	1	NA		1	feeding	1	0	0	0	0	0	0	NA			NA	Warmblood	1	350	3	cooperativeness									
157	gen.Germ.	video	3	2075,0	open_door_41	pony opens stable door of other horse so	1	NA		3	social	0	0	0	1	1	0	0	NA			NA	Warmblood	1	350	3	cooperativ									https://www.youtube.com/watch?v=Y
158	gen.Engl.	quest	1	2076,0	feeding_station_3	opening exit door of feeding station when	1	dayly	5	1	feeding	1	0	0	0	0	0	0	g	2	10,0	Paso Peruano	Warmblood	1	350	3	cooperativ	2	group	2	unrestricted	2	unrestricted	2	unrestrict	
159	gen.Engl.	quest	1	2077,0	open_box_9	opening feeding bin by grapping handle	1	dayly	5	1	feeding	1	0	0	0	0	0	0	g	2	6,0	Swedish	Draught horse	4	500	4	power	2	group	2	unrestricted	2	unrestricted	1	restricted	
160	gen.Engl.	video	3	2079,0	open_door_42	opening door by streching its neck to the	1	NA		2	escape	0	1	0	0	0	0	0	g	2		NA	Warmblood	1	350	3	cooperativ	1	single							http://youtu.be/DuleXhBpZKs
161	gen.Germ.	video	3	2080,0	open_door_43	picking up blankets and throwing them	1	NA		2	escape	0	1	0	0	0	0	0	m	1		NA	Warmblood	1	350	3	cooperativ	1	single							http://youtu.be/LS69RAiOOus
162	gen.Engl.	video	3	2081,0	open_door_44	opening bolt/door	1	NA		2	escape	0	1	0	0	0	0	0	m	1		NA	Warmblood	1	350	3	cooperativ	1	single							http://youtu.be/_UIByc0rlyA
163	gen.Engl.	video	3	2082,0	open_door_45	putting head through gate, lifting it up and	1	NA		2	escape	0	1	0	0	0	0	0	g	2		NA	Warmblood	1	350	3	cooperativ									
164	gen.Engl.	video	3	2083,0	open_door_46	opening bolt/door	1	NA		2	escape	0	1	0	0	0	0	0	m	1		Arabian	Arabian horse	3	4000	1	endurance									My horse unlocks her door!_0.mp4
165	gen.Engl.	video	3	2084,0	open_door_47	opening bolt/door, going to hay	1	NA		2	escape	0	1	0	0	0	0	0	m	1		NA	Warmblood	1	350	3	cooperativ	1	single							http://youtu.be/a1IAVrKHtlA
166	gen.Engl.	video	3	2085,0	open_door_48	opening bolt/door from inside	1	NA		5	play	0	0	0	0	0	0	1	NA			NA	Warmblood	1	350	3	cooperativ	1	single							http://youtu.be/1XWFjUjNq3E
167	gen.Engl.	video	3	2086,0	open_door_50	opening bolt/door (5 times)	1	NA		2	escape	0	1	0	0	0	0	0	m	1		NA	Warmblood	1	350	3	cooperativ									http://youtu.be/Ntydqyo6D8o
168	gen.Engl.	video	3	2087,0	open_door_51	opening bolt/door	1	NA		2	escape	0	1	0	0	0	0	0	g	2		NA	Warmblood	1	350	3	cooperativ									http://youtu.be/awuHaxptcgc
169	gen.Engl.	video	3	2088,0	open_door_52	opening bolt/door	1	NA		2	escape	0	1	0	0	0	0	0	g	2		NA	Warmblood	1	350	3	cooperativ									http://youtu.be/8uJ05KN4rhg
170	gen.Engl.	video	3	2089,0	untie_itself_16	opening knot	1	NA		2	escape	0	1	0	0	0	0	0	NA			NA	Warmblood	1	350	3	cooperativ									

1	sorce	video / quest	video_quest_num	sID	name behaviour	behaviour	nr reported behaviours	how often was the behaviour shown	behaviour frequency	behaviour category num	behaviour_category	feeding behaviour	escape behaviour	social behaviour human	social behaviour horse	social behaviour	comfort behaviour	unknown play
171	gen.Engl.	video	3	2090,0	open_door_53	opening bolt/door	1	NA		2	escape	0	1	0	0	0	0	0
172	gen.Engl.	video	3	2091,0	open_door_54	opening vertical bolt	1	NA		2	escape	0	1	0	0	0	0	0
173	gen.Engl.	video	3	2093,0	open_door_55	opening kitchen door and inspecting trash	1	NA		1	feeding	1	0	0	0	0	0	0
174	gen.Engl.	video	3	2094,0	open_box_11	opening feed bin	1	NA		1	feeding	1	0	0	0	0	0	0
175	gen.Engl.	video	3	2095,0	open box	open feed box	1	NA		1	feeding	1	0	0	0	0	0	0
176	gen.Engl.	video	3	2096,0	open_door_56	opening door of stall	2	NA		2	escape	0	1	0	0	0	0	0
177	gen.Engl.	video	3	2096,0	open_door_56	opening door of feeding room	2	NA		1	feeding	1	0	0	0	0	0	0
178	gen.Engl.	video	3	2097,0	manipulate_human_	mare makes clear that she wants to go out	4	NA		2	escape	0	1	0	0	0	0	0
179	gen.Engl.	video	3	2097,0	manipulate_human_	mare makes clear that she wants to go out	4	NA		3	social	0	0	1	0	1	0	0
180	gen.Engl.	video	3	2097,0	manipulate_human_	mare makes clear that she wants to go out	4	NA		4	comfort	0	0	0	0	0	1	0
181	gen.Engl.	video	3	2097,0	manipulate_human_	mare makes clear that she wants to go out	4	NA		3	social	0	0	1	0	1	0	0
182	gen.Engl.	video	3	2101,0	harvesting_apples_	rearing to get apples and pulling on limb	1	NA		1	feeding	1	0	0	0	0	0	0
183	gen.Engl.	video	3	2103,0	open_door_57	opening bolt/door	1	NA		2	escape	0	1	0	0	0	0	0
184	gen.Engl.	video	3	2104,0	open_door_58	opening bolt/pasture gate	1	NA		2	escape	0	1	0	0	0	0	0
185	gen.Engl.	video	3	2106,0	open_door_59	opening bolt/door	1	NA		2	escape	0	1	0	0	0	0	0
186	gen.Engl.	video	3	2107,0	open_door_60	opening bolt/door	1	NA		2	escape	0	1	0	0	0	0	0
187	gen.Engl.	video	3	2108,0	untie_itself_17	take off halter by striping it off at a post	1	11-20 times	3	2	escape	0	1	0	0	0	0	0
188	gen.Engl.	video	3	2109,0	open_door_61	opening bolt/stable door from the	1	NA		3	social	0	0	0	1	1	0	0
189	gen.Engl.	video	3	2110,0	grabbing_bucket_6	Reaching edge of the bucket (hangs on a	1	NA		1	feeding	1	0	0	0	0	0	0
190	gen.Engl.	video	3	2111,0	tool_use_8	waving bucket to chase away another	1	NA		1	feeding	1	0	0	0	0	0	0
191	gen.Engl.	video	3	2112,0	open_door_62	opening door	1	NA		2	escape	0	1	0	0	0	0	0
192	gen.Engl.	video	3	2113,0	open_door_63	opening bolt/door	1	NA		2	escape	0	1	0	0	0	0	0
193	gen.Engl.	video	3	2114,0	borderline_tool_use	grabbing feeding bucket of another horse	1	NA		1	feeding	1	0	0	0	0	0	0
194	gen.Engl.	video	3	2115,0	untie_itself_18	opening safe release carabiner	1	NA		2	escape	0	1	0	0	0	0	0
195	gen.Engl.	video	3	2116,0	feeding_horse_2	feeding other horse (in box with saw dust)	1	NA		3	social	0	0	0	1	1	0	0
196	gen.Engl.	video	3	2118,0	open_door_64	opening gate	1	NA		2	escape	0	1	0	0	0	0	0
197	gen.Engl.	video	3	2119,0	feeding_under_fenc	eating grass under the fence by lying on	1	NA		1	feeding	1	0	0	0	0	0	0
198	gen.Engl.	video	3	2120,0	open_box_12	opening freezer (contains feed)	1	NA		1	feeding	1	0	0	0	0	0	0
199	gen.Engl.	video	3	2121,0	open_door_65	opening gate by biting in the wooden bars	1	NA		2	escape	0	1	0	0	0	0	0
200	gen.Engl.	video	3	2122,0	untie_itself_19	opening knot/untie itself	1	NA		2	escape	0	1	0	0	0	0	0
201	gen.Engl.	video	3	2123,0	open_door_66	opening different kinds of bolts, other	1	more than 20 times	4	2	escape	0	1	0	0	0	0	0
202	gen.Engl.	quest	1	2125,0	manipulate_horse_4	leading another horse on the halter while	1	2-10 times	2	3	social	0	0	0	1	1	0	0
203	gen.Engl.	quest	1	2128,0	untie_itself_20	take off halter by striping it off at a post	1	more than 20 times	4	2	escape	0	1	0	0	0	0	0
204	gen.Engl.	quest	1	2078,0	open_door_67	opening different kinds of gates,	3	dayly	5	2	escape	0	1	0	0	0	0	0
205	gen.Engl.	quest	1	2078,0	open_door_67	opening different kinds of gates, letting	3	dayly	5	3	social	0	0	0	1	1	0	0
206	gen.Engl.	quest	1	2078,0	open_door_67	opening different kinds of gates closing	3	dayly	5	5	play	0	0	0	0	0	0	1
207	gen.Engl.	quest	1	2117,0	open_door_68	opening door/bolt	1	11-20 times	3	2	escape	0	1	0	0	0	0	0
208	gen.Engl.	quest	1	2124,0	open_door_69	opening stall	7	2-10 times	2	2	escape	0	1	0	0	0	0	0
209	gen.Engl.	quest	1	2124,0	fence_15	crashes down wood fences that he knows	7	2-10 times	2	2	escape	0	1	0	0	0	0	0
210	gen.Engl.	quest	1	2124,0	untie_itself_21	untie knots	7	2-10 times	2	2	escape	0	1	0	0	0	0	0
211	gen.Engl.	quest	1	2124,0	untie_itself_22	taking off halters	7	2-10 times	2	2	escape	0	1	0	0	0	0	0
212	gen.Engl.	quest	1	2124,0	untie_itself_15	unsnap lead ropes(bull snaps or regular	7	2-10 times	2	2	escape	0	1	0	0	0	0	0
213	gen.Engl.	quest	1	2124,0	fence_16	crawling under fence when it is high	7	2-10 times	2	2	escape	0	1	0	0	0	0	0
214	gen.Engl.	quest	1	2126,0	untie_itself_23	unsnap lead ropes	7	more than 20 times	4	2	escape	0	1	0	0	0	0	0
215	gen.Engl.	quest	1	2126,0	open_door_70	opening gates	2	more than 20 times	4	2	escape	0	1	0	0	0	0	0
216	gen.Engl.	quest	1	2126,0	open_door_70	unty lead ropes	2	more than 20 times	4	2	escape	0	1	0	0	0	0	0
217	gen.Engl.	quest	1	2127,0	open_door_71	opening different kinds of gates when	1	dayly	5	2	escape	0	1	0	0	0	0	0
218	gen.Germ.	video	3	2130,0	untie_itself_24	opening knot/untie itsself	1	NA		2	escape	0	1	0	0	0	0	0
219	gen.Germ.	quest	1	2131,0	open_door_72	opening door/bolt (twice)	1	NA		2	escape	0	1	0	0	0	0	0
220	gen.Germ.	video	3	2132,0	feeding_under_fenc	eating grass under the fence by liying on	1	NA		1	feeding	1	0	0	0	0	0	0
221	gen.Germ.	video	3	2133,0	open_door_73	opening door/bolt	1	NA		2	escape	0	1	0	0	0	0	0
222	gen.Germ.	video	3	2134,0	open_door_74	opening door (sliding) / unlock	1	NA		2	escape	0	1	0	0	0	0	0
223	gen.Germ.	video	3	2135,0	open_door_75	opening door/ bolt	1	NA		2	escape	0	1	0	0	0	0	0
224	gen.Germ.	video	3	2136,0	open_door_76	opening door/ handle (normal "human"	1	NA		1	feeding	1	0	0	0	0	0	0
225	gen.Germ.	video	3	2137,0	open_door_77	opening door/ handle (normal "human"	1	NA		1	feeding	1	0	0	0	0	0	0
226	gen.Germ.	video	3	2138,0	open_door_78	opening gate by biting in the wooden bars	1	NA		2	escape	0	1	0	0	0	0	0
227	gen.Germ.	video	3	2140,0	untie_itself_25	unsnap lead rope with security hook (to	1	NA		2	escape	0	1	0	0	0	0	0
228	gen.Germ.	video	3	2141,0	open_box_13	opening feed bin (trash Can)	1	NA		1	feeding	1	0	0	0	0	0	0
229	gen.Engl.	quest	1	2146,0	open door - gate	open door - gate	1	more than 20 times	4	2	escape	0	1	0	0	0	0	0
230	gen.Engl.	quest	1	2147,0	untie_itself_26	untying knots	3	dayly	5	2	escape	0	1	0	0	0	0	0

1	sex	sex num	age	breed	breed-type	breed type num	length of domestication	purpose of domestication	purpose of domestication	single group stabling	single group stabling	restricted unrestricted pasture	restricted unrestricted pasture	restricted unrestricted contact with horses	restricted unrestricted contact with horses	restricted unrestric. roughage	restricted unrestricted roughage	links
171	m	1		NA	Warmblood	1	350	3	cooperativ									http://youtu.be/BLejxlfZkkA
172	g	2		NA	Warmblood	1	350	3	cooperativ									http://youtu.be/EJDoCMj-WIQ
173	NA			NA	Pony	2	100	5	robustnes									http://youtu.be/7Zf4IbMe7VM
174	NA			NA	Warmblood	1	350	3	cooperativ									
175	NA			NA	Warmblood	1	350	3	cooperativ									http://youtu.be/OYW5oUQGRLk
176	NA			NA	NA													http://youtu.be/90Xqn0uUnaY
177	NA			NA	NA													http://youtu.be/90Xqn0uUnaY
178	m	1		NA	Warmblood	1	350	3	cooperativ									
179	m	1		NA	Warmblood	1	350	3	cooperativ									
180	m	1		NA	Warmblood	1	350	3	cooperativ									
181	m	1		NA	Warmblood	1	350	3	cooperativ									
182	m	1		NA	Warmblood	1	350	3	cooperativ									
183	NA			NA	Arabian horse	3	4000	1	endurance									http://youtu.be/vFc9bQ0Vs3Q
184	g	2		NA	Warmblood	1	350	3	cooperativ									http://youtu.be/UnuuukezlUw
185	g	2		NA	Warmblood	1	350	3	cooperativ									http://youtu.be/0SDpaxilWfI
186	g	2		NA	Warmblood	1	350	3	cooperativ									http://youtu.be/nY2owCKXAr4
187	s	3		Iceland horse	Pony	2	100	5	robustnes									
188	g	2		NA	Warmblood	1	350	3	cooperativ									http://youtu.be/MTg4M5D3fH4
189	NA			Miniature	Pony	2	100	5	robustnes									
190	NA			NA	Warmblood	1	350	3	cooperativ									
191	g	2		NA	Warmblood	1	350	3	cooperativ									
192	NA			NA	Warmblood	1	350	3	cooperativ									http://youtu.be/M-LvDjityTM
193	m	1		NA	Warmblood	1	350	3	cooperativ									
194	g	2		NA	Warmblood	1	350	3	cooperativ									
195	NA			NA	NA													
196	g	2		NA	Mule	7	3000	4	power									
197	NA		0,5	NA	Warmblood	1	350	3	cooperativ									
198	NA			NA	Warmblood	1	350	3	cooperativ									
199	g	2		NA	Warmblood	1	350	3	cooperativ									http://youtu.be/T7wi6Bt8O3g
200	g	2	12,0	NA	Warmblood	1	350	3	cooperativ									
201	g	2		NA	NA					1	single							
202	g	2	7,0	Iceland horse	Pony	2	100	5	robustnes	2	group	2	unrestricted	2	unrestricted	2	unrestrict	
203	m	1	5,0	Iceland horse	Pony	2	100	5	robustnes	2	group	2	unrestricted	2	unrestricted	1	restricted	
204	g	2	2,0	Appaloosa	Warmblood	1	350	3	cooperativ	2	group	2	unrestricted	2	unrestricted	2	unrestrict	
205	g	2	2,0	Appaloosa	Warmblood	1	350	3	cooperativ	2	group	2	unrestricted	2	unrestricted	2	unrestrict	
206	g	2	2,0	Appaloosa	Warmblood	1	350	3	cooperativ	2	group	2	unrestricted	2	unrestricted	2	unrestrict	
207	s	3	5,0	Iceland horse	Pony	2	100	5	robustnes	1	single	2	unrestricted	1	restricted	1	restricted	
208	s	3	5,0	Iceland horse	Pony	2	100	5	robustnes	1	single	2	unrestricted	1	restricted	1	restricted	
209	s	3	5,0	Iceland horse	Pony	2	100	5	robustnes	1	single	2	unrestricted	1	restricted	1	restricted	
210	s	3	5,0	Iceland horse	Pony	2	100	5	robustnes	1	single	2	unrestricted	1	restricted	1	restricted	
211	s	3	5,0	Iceland horse	Pony	2	100	5	robustnes	1	single	2	unrestricted	1	restricted	1	restricted	
212	s	3	5,0	Iceland horse	Pony	2	100	5	robustnes	1	single	2	unrestricted	1	restricted	1	restricted	
213	s	3	5,0	Iceland horse	Pony	2	100	5	robustnes	1	single	2	unrestricted	1	restricted	1	restricted	
214	g	2	4,0	Paint horse	Warmblood	1	350	3	cooperativ	1	single	2	unrestricted	2	unrestricted	2	unrestrict	
215	g	2	4,0	Paint horse	Warmblood	1	350	3	cooperativ	1	single	2	unrestricted	2	unrestricted	2	unrestrict	
216	g	2	4,0	Paint horse	Warmblood	1	350	3	cooperativ	1	single	2	unrestricted	2	unrestricted	2	unrestrict	
217	g	2	9,0	Quarter horse	Warmblood	1	350	3	cooperativ	2	group	2	unrestricted	2	unrestricted	1	restricted	
218	m	1		Arab mix	Arabian horse	3	4000	1	endurance									
219	m	1		NA	Warmblood	1	350	3	cooperativ									http://youtu.be/osbxMbHUNe8
220	g	2		NA	Warmblood	1	350	3	cooperativ									
221	g	2		NA	Arabian horse	3	4000	1	endurance									http://youtu.be/m2B3_qwwglM
222	NA			NA	NA													http://youtu.be/jyWR6F-EaeY
223	g	2		NA	Warmblood	1	350	3	cooperativ									http://youtu.be/AzLToOWyXyk
224	m	1		NA	Warmblood	1	350	3	cooperativ									http://youtu.be/1yLYMfwFqis
225	g	2		NA	Pony	2	100	5	robustnes									http://youtu.be/TipjTiYOgE4
226	NA			NA	NA													http://youtu.be/b0BUqwqLI3wNAt=31s
227	m	1		NA	Pony	2	100	5	robustnes									
228	NA			NA	Warmblood	1	350	3	cooperativ									
229	m	1	1,0	NA	Warmblood	1	350	3	cooperativ	1	single	2	unrestricted	2	unrestricted	1	restricted	
230	g	2	7,0	Paint horse	Warmblood	1	350	3	cooperativ	1	single	2	unrestricted	2	unrestricted	1	restricted	

1	sorce	video / quest	video_quest_num	sID	name behaviour	behaviour	nr reported behaviours	how often was the behaviour shown	behaviour frequency	behaviour category num	behaviour_category	feeding behaviour	escape behaviour	social behaviour human	social behaviour horse	social behaviour	comfort behaviour	unknown play	sex	sex num	age	breed	breed-type	breed type num	length of domestication	purpose of domestication	purpose of domestication	single group stabling	single group stabling	restricted unrestricted pasture	restricted unrestricted pasture	restricted unrestricted contact with horses	restricted unrestricted contact with horses	restricted unrestric. roughage	restricted unrestricted roughage	links
231	gen.Engl.	quest	1	2147,0	manipulate_horse_5	taking of head-gears of other horses	3	dayly	5	3	social	0	0	0	1	1	0	0	g	2	7,0	Paint horse	Warmblood	1	350	3	cooperativ	1	single	2	unrestricted	2	unrestricted	1	restricted	
232	gen.Engl.	quest	1	2147,0	fence_17	Looking for weak spots in the fence	3	dayly	5	2	escape	0	1	0	0	0	0	0	g	2	7,0	Paint horse	Warmblood	1	350	3	cooperativ	1	single	2	unrestricted	2	unrestricted	1	restricted	
233	gen.Engl.	quest	1	2148,0	fence_18	opening main entrance to pasture	1	11-20 times	3	2	escape	0	1	0	0	0	0	0	s	3	3,0	Rocky	Warmblood	1	350	3	cooperativ	2	group	2	unrestricted	2	unrestricted	2	unrestrict	
234	gen.Engl.	quest	1	2150,0	untie_itself_27	untie knots	3	more than 20 times	4	2	escape	0	1	0	0	0	0	0	g	2	26,0	Selle Francais	Warmblood	1	350	3	cooperativ	2	group	2	unrestricted	2	unrestricted	2	unrestrict	
235	gen.Engl.	quest	1	2150,0	open_door_80	opening locked stabledoors	3	more than 20 times	4	2	escape	0	1	0	0	0	0	0	g	2	26,0	Selle Francais	Warmblood	1	350	3	cooperativ	2	group	2	unrestricted	2	unrestricted	2	unrestrict	
236	gen.Engl.	quest	1	2150,0	open_door_80	unty knots	3	more than 20 times	4	2	escape	0	1	0	0	0	0	0	g	2	26,0	Selle Francais	Warmblood	1	350	3	cooperativ	2	group	2	unrestricted	2	unrestricted	2	unrestrict	
237	gen.Engl.	quest	1	2152,0	open_door_81	opening different kind of doors, feed room	4	more than 20 times	4	1	feeding	1	0	0	0	0	0	0	g	2	15,0	Danish Warmblood	Warmblood	1	350	3	cooperativeness	1	single	2	unrestricted pasture	2	unrestricted contact	2	unrestrict roughage	
238	gen.Engl.	quest	1	2152,0	open_door_81	opening different kind of doors, own door	4	more than 20 times	4	2	escape	0	1	0	0	0	0	0	g	2	15,0	Danish Warmblood	Warmblood	1	350	3	cooperativeness	1	single	2	unrestricted pasture	2	unrestricted contact	2	unrestrict roughage	
239	gen.Engl.	quest	1	2152,0	untie_itself_28	untie knots	4	more than 20 times	4	2	escape	0	1	0	0	0	0	0	g	2	15,0	Danish Warmblood	Warmblood	1	350	3	cooperativeness	1	single	2	unrestricted pasture	2	unrestricted contact	2	unrestrict roughage	
240	gen.Engl.	quest	1	2152,0	grabbing_bucket_7	throwing buckets from all horses out of box onto stable hallway	4	more than 20 times	4	5	play	0	0	0	0	0	0	1	g	2	15,0	Danish Warmblood	Warmblood	1	350	3	cooperativeness	1	single	2	unrestricted pasture	2	unrestricted contact	2	unrestrict roughage	
241	gen.Germ.	quest	1	2151,0	manipulate_horse_6	showing exercises from training in	1	dayly	5	3	social	0	0	0	1	1	0	0	g	2	9,0	Hannoveraner	Warmblood	1	350	3	cooperativ	2	group	2	unrestricted	2	unrestricted	2	unrestrict	
242	gen.Germ.	quest	1	2155,0	fence_20	going through fences when electricity is	1	2-10 times	2	2	escape	0	1	0	0	0	0	0	m	1		Hannoveraner	Warmblood	1	350	3	cooperativ	1	single	2	unrestricted	2	unrestricted	1	restricted	
243	gen.Engl.	quest	1	2153,0	open_door_82	opening own stable door	2	11-20 times	3	2	escape	0	1	0	0	0	0	0	m	1	17,0	KWPN	Warmblood	1	350	3	cooperativ	1	single	2	unrestricted	2	unrestricted	1	restricted	
244	gen.Engl.	quest	1	2153,0	open_door_82	opening stable doors of other horses	2	11-20 times	3	3	social	0	0	0	1	1	0	0	m	1	17,0	KWPN	Warmblood	1	350	3	cooperativ	1	single	2	unrestricted	2	unrestricted	1	restricted	
245	gen.Germ.	quest	1	2156,0	tool_use_9	Using a stick to scratch out hay from the hay rack	2	more than 20 times	4	1	feeding	1	0	0	0	0	0	0	g	2	6,0	Scottish Highland	Warmblood	1	350	3	cooperativeness	2	group	2	unrestricted pasture	2	unrestricted contact	1	restricted roughage	
246	gen.Germ.	quest	1	2156,0	manipulate_things_17	Shaking feeding bucket so that food falls to one side of the bucket	2	more than 20 times	4	1	feeding	1	0	0	0	0	0	0	g	2	6,0	Scottish Highland	Warmblood	1	350	3	cooperativeness	2	group	2	unrestricted pasture	2	unrestricted contact	1	restricted roughage	
247	gen.Germ.	quest	1	2157,0	manipulate_things_19	Opening water-tap with ist mouth	2	NA		1	feeding	1	0	0	0	0	0	0	g	2	4,0	Hannoveraner	Warmblood	1	350	3	cooperativeness	2	group	2	unrestricted pasture	2	unrestricted contact	2	unrestrict roughage	
248	gen.Germ.	quest	1	2157,0	untie_itself_29	Opening carabiner when standing on trailer	2	NA		5	play	0	0	0	0	0	0	1	g	2	4,0	Hannoveraner	Warmblood	1	350	3	cooperativeness	2	group	2	unrestricted pasture	2	unrestricted contact	2	unrestrict roughage	
249	gen.Germ.	quest	1	2158,0	open door - gate	open door - gate	1	dayly	5	1	feeding	1	0	0	0	0	0	0	s	3	10,0	NA	NA					1	single	2	unrestricted pasture	2	unrestricted contact	1	restricted roughage	
250	gen.Germ.	quest	1	2159,0	feeding_station_4	Slipping through feeding station behinde other horse into area with hay, holding head up so that transponder cannot be	1	11-20 times	3	1	feeding	1	0	0	0	0	0	0	g	2	8,0	Trakehner	Warmblood	1	350	3	cooperativeness	2	group	2	unrestricted pasture	2	unrestricted contact	2	unrestrict roughage	
251	gen.Germ.	quest	1	2161,0	manipulate_horse_	Running out of stable as if there's sth. dangerous, other horse follow, going back and feeding on roughage of the	1	11-20 times	3	3	social	0	0	1	0	1	0	0	g	2	5,0	Arab mix	Arabian horse	3	4000	1	endurance	2	group	2	unrestricted pasture	2	unrestricted contact	2	unrestrict roughage	
252	gen.Germ.	quest	1	2164,0	feeding_station_5	Eating feed of others in feeding station (by letting exit door lean against the body and pushing horse backwards after it got	1	dayly	5	1	feeding	1	0	0	0	0	0	0	g	2	8,0	Friesian horse	Warmblood	1	350	3	cooperativeness	2	group	2	unrestricted pasture	2	unrestricted contact	1	restricted roughage	
253	gen.Germ.	quest	1	2165,0	manipulate_things_21	Switching light on and off in the stable	1	more than 20 times	4	5	play	0	0	0	0	0	0	1	g	2	9,0	Hannoveraner	Warmblood	1	350	3	cooperativeness	2	group	2	unrestricted pasture	2	unrestricted contact	1	restricted roughage	
254	gen.Germ.	quest	1	2166,0	open_box_14	Opening hatch of other boxes through which horses are fed	1	dayly	5	1	feeding	1	0	0	0	0	0	0	m	1	10,0	NA	NA					1	single	1	restricted pasture	1	restricted	1	restricted roughage	
255	gen.Engl.	quest	1	2162,0	saddle_off_1	Removing saddle when saddle pad no longer fitted, stopping behaviour when saddle/pad replaced	1	2-10 times	2	4	comfort	0	0	0	0	0	1	0	g	2	8,0	Trakehner Arabian mix	Warmblood	1	350	3	cooperativeness	2	group	2	unrestricted pasture	2	unrestricted contact	2	unrestrict roughage	
256	gen.Engl.	quest	1	2163,0	open_door_83	Opening gates	3	dayly	5	2	escape	0	1	0	0	0	0	0	s	3	15,0	PRE	Warmblood	1	350	3	cooperativeness	2	group	2	unrestricted pasture	2	unrestricted contact	2	unrestrict roughage	
257	gen.Engl.	quest	1	2163,0	untie_itself	Untying knots	3	dayly	5	2	escape	0	1	0	0	0	0	0	s	3	15,0	PRE	Warmblood	1	350	3	cooperativeness	2	group	2	unrestricted pasture	2	unrestricted contact	2	unrestrict roughage	
258	gen.Engl.	quest	1	2163,0	untie_itself	taking off halter	3	dayly	5	2	escape	0	1	0	0	0	0	0	s	3	15,0	PRE	Warmblood	1	350	3	cooperativeness	2	group	2	unrestricted pasture	2	unrestricted contact	2	unrestrict roughage	
259	gen.Engl.	quest	1	2168,0	open_door_84	Opening different kind of doors , own door, own door	5	NA		2	escape	0	1	0	0	0	0	0	g	2	10,0	Arabian	Arabian horse	3	4000	1	endurance	2	group	2	unrestricted pasture	2	unrestricted contact	2	unrestrict roughage	
260	gen.Engl.	quest	1	2168,0	open_door_84	Opening different kind of doors , own door	5	NA		3	social	0	0	0	1	1	0	0	g	2	10,0	Arabian	Arabian horse	3	4000	1	endurance	2	group	2	unrestricted pasture	2	unrestricted contact	2	unrestrict roughage	
261	gen.Engl.	quest	1	2168,0	open_door_84	Opening different kind of doors , letting other horses out, frees other horses	5	NA		3	social	0	0	0	1	1	0	0	g	2	10,0	Arabian	Arabian horse	3	4000	1	endurance	2	group	2	unrestricted pasture	2	unrestricted contact	2	unrestrict roughage	
262	gen.Engl.	quest	1	2168,0	untie_itself_31	Untying ropes,	5	2-10 times	2	2	escape	0	1	0	0	0	0	0	g	2	10,0	Arabian	Arabian horse	3	4000	1	endurance	2	group	2	unrestricted pasture	2	unrestricted contact	2	unrestrict roughage	
263	gen.Engl.	quest	1	2168,0	untie_itself_31	Untying ropes, from other horses	5	2-10 times	2	3	social	0	0	0	1	1	0	0	g	2	10,0	Arabian	Arabian horse	3	4000	1	endurance	2	group	2	unrestricted pasture	2	unrestricted contact	2	unrestrict roughage	
264	gen.Germ.	quest	1	2169,0	open_door_85	Opening doors	1	NA		2	escape	0	1	0	0	0	0	0	g	2	7,0	NA	NA					2	group	2	unrestricted pasture	2	unrestricted contact	2	unrestrict roughage	
265	gen.Engl.	quest	1	2173,0	open_door_87	Opening treegate. This gate is closed with a rope. He is lifting the rope up and pulling the gate towards himself and moves backwards. He wants to gain fresh grass.	1	2-10 times	2	2	escape	0	1	0	0	0	0	0	g	2	7,0	Iceland horse	Pony	2	100	5	robustness	2	group	2	unrestricted pasture	2	unrestricted contact	2	unrestrict roughage	
266	gen.Germ.	quest	1	2174,0	untie_itself	remove halter when tied	4	2-10 times	2	2	escape	0	1	0	0	0	0	0	g	2	16,0	Fjordhorse	Warmblood	1	350	3	cooperativeness	2	group	2	unrestricted pasture	2	unrestricted contact	2	unrestrict roughage	

1	sorce	video / quest	video_quest_num	sID	name behaviour	behaviour	nr reported behaviours	how often was the behaviour shown	behaviour frequency	behaviour category num	behaviour_category	feeding behaviour	escape behaviour	social behaviour human	social behaviour horse	social behaviour	comfort behaviour	unknown play	sex	sex num	age	breed	breed-type	breed type num	length of domestication	purpose of domestication	purpose of domestication	single group stabling	single group stabling	restricted unrestricted pasture	restricted unrestricted pasture	restricted unrestricted contact with horses	restricted unrestricted contact with horses	restricted unrestric. roughage	restricted unrestricted roughage	links
267	gen.Germ.	quest	1	2174,0	untie itself	unty knots	4	2-10 times	2	2	escape	0	1	0	0	0	0	0	g	2	16,0	Fjordhorse	Warmblood	1	350	3	cooperativeness	2	group	2	unrestricted pasture	2	unrestricted contact	2	unrestrict roughage	
268	gen.Germ.	quest	1	2174,0	open door	open door	4	2-10 times	2	2	escape	0	1	0	0	0	0	0	g	2	16,0	Fjordhorse	Warmblood	1	350	3	cooperativeness	2	group	2	unrestricted pasture	2	unrestricted contact	2	unrestrict roughage	
269	gen.Germ.	quest	1	2174,0	manipulate humans	manipulate humans	4	2-10 times	2	3	social	0	0	1	0	1	0	0	g	2	16,0	Fjordhorse	Warmblood	1	350	3	cooperativeness	2	group	2	unrestricted pasture	2	unrestricted contact	2	unrestrict roughage	
270	gen.Germ.	quest	1	2175,0	open_door	opens many different doors	3	dayly	5	5	play	0	0	0	0	0	0	1	g	2	7,0	Württemberger	Warmblood	1	350	3	cooperativeness	2	group	2	unrestricted pasture	2	unrestricted contact	2	unrestrict roughage	
271	gen.Germ.	quest	1	2175,0	manipulate things	take down rags	3	dayly	5	4	comfort	0	0	0	0	0	1	0	g	2	7,0	Württemberger	Warmblood	1	350	3	cooperativeness	2	group	2	unrestricted pasture	2	unrestricted contact	2	unrestrict roughage	
272	gen.Germ.	quest	1	2175,0	manipulate things	open boxes	3	dayly	5	5	play	0	0	0	0	0	0	1	g	2	7,0	Württemberger	Warmblood	1	350	3	cooperativeness	2	group	2	unrestricted pasture	2	unrestricted contact	2	unrestrict roughage	
273	gen.Germ.	quest	1	2188,0	untie_itself	opening knots of the rope when tied	2	more than 20 times	4	2	escape	0	1	0	0	0	0	0	m	1	13,0	Quarter horse	Warmblood	1	350	3	cooperativeness	1	single	2	unrestricted pasture	2	unrestricted contact	2	unrestrict roughage	
274	gen.Germ.	quest	1	2188,0	untie_itself	remove halter	2	more than 20 times	4	2	escape	0	1	0	0	0	0	0	m	1	13,0	Quarter horse	Warmblood	1	350	3	cooperativeness	1	single	2	unrestricted pasture	2	unrestricted contact	2	unrestrict roughage	
275	gen.Germ.	quest	1	2191,0	manipulate things	stepp on water hoss when being cleaned	1	11-20 times	3	4	comfort	0	0	0	0	0	1	0	g	2	5,0	Arab mix	Arabian horse	3	4000	1	endurance	1	single	1	restricted	2	unrestricted	1	restricted	
276	gen.Germ.	quest	1	2193,0	untie itself	unty knots,	1	11-20 times	3	2	escape	0	1	0	0	0	0	0	g	2	4,0	Haflinger	Warmblood	1	350	3	cooperativeness	2	group	2	unrestricted pasture	2	unrestricted contact	2	unrestrict roughage	
277	gen.Germ.	quest	1	2194,0	untie itself	take off halter	1	dayly	5	2	escape	0	1	0	0	0	0	0	g	2	4,0	Haflinger	Warmblood	1	350	3	cooperativeness	2	group	2	unrestricted pasture	2	unrestricted contact	2	unrestrict roughage	
278	gen.Germ.	quest	1	2217,0	open door	opens door handles	1	dayly	5	1	feeding	1	0	0	0	0	0	0	g	2	5,0	Arab mix	Arabian horse	3	4000	1	endurance	2	group	2	unrestricted pasture	2	unrestricted contact	2	unrestrict roughage	
279	gen.Germ.	quest	1	2225,0	manipulate things	take off rag, blankets	1	more than 20 times	4	4	comfort	0	0	0	0	0	1	0	m	1	10,0	Westfahle	Warmblood	1	350	3	cooperativeness	1	single	1	restricted pasture	1	restricted	1	restricted roughage	
280	gen.Germ.	quest	1	2231,0	unty itself	take off halter	2	NA		2	escape	0	1	0	0	0	0	0	m	1	9,0	Lewitzer	Draught horse	4	500	4	power	1	single	2	unrestricted pasture	2	unrestricted contact	2	unrestrict roughage	
281	gen.Germ.	quest	1	2231,0	manipulate things	take off bandages, leg protection	2	NA		4	comfort	0	0	0	0	0	1	0	m	1	9,0	Lewitzer	Draught horse	4	500	4	power	1	single	2	unrestricted pasture	2	unrestricted contact	2	unrestrict roughage	
282	gen.Germ.	quest	1	2233,0	open door	open door	2	more than 20 times	4	1	feeding	1	0	0	0	0	0	0	m	1	28,0	Haflinger	Warmblood	1	350	3	cooperativeness	2	group	2	unrestricted pasture	2	unrestricted contact	2	unrestrict roughage	
283	gen.Germ.	quest	1	2233,0	manipulate things	open food box	2	more than 20 times	4	1	feeding	1	0	0	0	0	0	0	m	1	28,0	Haflinger	Warmblood	1	350	3	cooperativeness	2	group	2	unrestricted pasture	2	unrestricted contact	2	unrestrict roughage	
284	gen.Germ.	quest	1	2238,0	open door	open gates, elctric fence handle	3	more than 20 times	4	1	feeding	1	0	0	0	0	0	0	m	1	6,0	Freiberger	Warmblood	1	350	3	cooperativeness	2	group	2	unrestricted pasture	2	unrestricted contact	2	unrestrict roughage	
285	gen.Germ.	quest	1	2238,0	manipulate things	open feed box, also when secured with carabina	3	more than 20 times	4	1	feeding	1	0	0	0	0	0	0	m	1	6,0	Freiberger	Warmblood	1	350	3	cooperativeness	2	group	2	unrestricted pasture	2	unrestricted contact	2	unrestrict roughage	
286	gen.Germ.	quest	1	2238,0	untie itself	unty knots	3	more than 20 times	4	2	escape	0	1	0	0	0	0	0	m	1	6,0	Freiberger	Warmblood	1	350	3	cooperativeness	2	group	2	unrestricted pasture	2	unrestricted contact	2	unrestrict roughage	
287	gen.Germ.	quest	1	2251,0	open door	open door of trailor from the inside	2	2-10 times	2	2	escape	0	1	0	0	0	0	0	g	2	7,0	Haflinger	Warmblood	1	350	3	cooperativeness	1	single	2	unrestricted pasture	2	unrestricted contact	2	unrestrict roughage	
288	gen.Germ.	quest	1	2251,0	manipulate things	open water tap	2	2-10 times	2	1	feeding	1	0	0	0	0	0	0	g	2	7,0	Haflinger	Warmblood	1	350	3	cooperativeness	1	single	2	unrestricted pasture	2	unrestricted contact	2	unrestrict roughage	
289	gen.Germ.	quest	1	2282,0	manipulate things	open feed box	1	2-10 times	2	1	feeding	1	0	0	0	0	0	0	g	2	19,0	Paint horse	Warmblood	1	350	3	cooperativeness	1	single	2	unrestricted pasture	2	unrestricted contact	2	unrestrict roughage	
290	gen.Germ.	quest	1	2297,0	open door	open door	6	more than 20 times	4	2	escape	0	1	0	0	0	0	0	m	1	5,0	Hannoveraner	Warmblood	1	350	3	cooperativeness	1	single	2	unrestricted pasture	2	unrestricted contact	1	restricted roughage	
291	gen.Germ.	quest	1	2297,0	open door	open gate, electric fence handles	6	2-10 times	2	2	escape	0	1	0	0	0	0	0	m	1	5,0	Hannoveraner	Warmblood	1	350	3	cooperativeness	1	single	2	unrestricted pasture	2	unrestricted contact	1	restricted roughage	
292	gen.Germ.	quest	1	2297,0	manipulate things	open water tap	6	more than 20 times	4	1	feeding	1	0	0	0	0	0	0	m	1	5,0	Hannoveraner	Warmblood	1	350	3	cooperativeness	1	single	2	unrestricted pasture	2	unrestricted contact	1	restricted roughage	
293	gen.Germ.	quest	1	2297,0	manipulate things	turn light on	6	more than 20 times	4	5	play	0	0	0	0	0	0	1	m	1	5,0	Hannoveraner	Warmblood	1	350	3	cooperativeness	1	single	2	unrestricted pasture	2	unrestricted contact	1	restricted roughage	
294	gen.Germ.	quest	1	2297,0	unttie itself	unty knots	6	more than 20 times	4	2	escape	0	1	0	0	0	0	0	m	1	5,0	Hannoveraner	Warmblood	1	350	3	cooperativeness	1	single	2	unrestricted pasture	2	unrestricted contact	1	restricted roughage	
295	gen.Germ.	quest	1	2297,0	unttie itself	take off halters	6	more than 20 times	4	2	escape	0	1	0	0	0	0	0	m	1	5,0	Hannoveraner	Warmblood	1	350	3	cooperativeness	1	single	2	unrestricted pasture	2	unrestricted contact	1	restricted roughage	
296	gen.Germ.	quest	1	2299,0	manipulate things	close water tab, step on water hose	1	more than 20 times	4	4	comfort	0	0	0	0	0	1	0	m	1	6,0	Westfahle	Warmblood	1	350	3	cooperativeness	1	single	2	unrestricted pasture	2	unrestricted contact	2	unrestrict roughage	
297	gen.Germ.	quest	1	2327,0	manipulate horses	take off muzzel	2	dayly	5	1	feeding	1	0	0	0	0	0	0	g	2	9,0	Arab mix	Arabian horse	3	4000	1	endurance	2	group	2	unrestricted pasture	2	unrestricted contact	1	restricted roughage	
298	gen.Germ.	quest	1	2327,0	manipulate horses	approach other horses for taking muzzle off	2	dayly	5	3	social	0	0	0	1	1	0	0	g	2	9,0	Arab mix	Arabian horse	3	4000	1	endurance	2	group	2	unrestricted pasture	2	unrestricted contact	1	restricted roughage	
299	gen.Germ.	quest	1	2377,0	manipulate things	opens zippers and Velcro strips	1	dayly	5	5	play	0	0	0	0	0	0	1	m	1	26,0	Oldenburg horse	Warmblood	1	350	3	cooperativeness	1	single	2	unrestricted pasture	2	unrestricted contact	1	restricted roughage	
300	gen.Germ.	quest	1	2404,0	open door	open door to get in	1	dayly	5	3	social	0	0	0	1	1	0	0	g	2	10,0	Rheinisches Warmblood	Warmblood	1	350	3	cooperativeness	2	group	2	unrestricted pasture	2	unrestricted contact	2	unrestrict roughage	

1	sorce	video / quest	video_quest_num	sID	name behaviour	behaviour	nr reported behaviours	how often was the behaviour shown	behaviour frequency	behaviour category num	behaviour_category	feeding behaviour	escape behaviour	social behaviour human	social behaviour horse	social behaviour	comfort behaviour	unknown play	sex	sex num	age	breed	breed-type	breed type num	length of domestication	purpose of domestication	purpose of domestication	single group stabling	single group stabling	restricted unrestricted pasture	restricted unrestricted pasture	restricted unrestricted contact with horses	restricted unrestricted contact with horses	restricted unrestric. roughage	restricted unrestricted roughage	links
301	gen.Germ.	quest	1	2405,0	open door	open gate	1	2-10 times	2	2	escape	0	1	0	0	0	0	0	g	2	12,0	Oldenburg horse	Warmblood	1	350	3	cooperativeness	2	group	2	unrestricted pasture	2	unrestricted contact	2	unrestrict roughage	
302	gen.Germ.	quest	1	2441,0	manipulate things	kicks food out of food bucket by pushing with ist head against the hanging buckket from underneath. Food pops out.	1	dayly	5	1	feeding	1	0	0	0	0	0	0	g	2	3,0	Arab mix	Arabian horse	3	4000	1	endurance	2	group	2	unrestricted pasture	2	unrestricted contact	2	unrestrict roughage	
303	gen.Germ.	quest	1	2251,0	open door - gate	open door - gate	2	2-10 times	2	2	escape	0	1	0	0	0	0	0	g	2	7,0	NA	Warmblood	1	350	3	cooperativeness	1	single	1	restricted pasture	1	restricted	1	restricted roughage	
304	gen.Germ.	quest	1	2251,0	manipulate_thing	open water tap	2	2-10 times	2	1	feeding	1	0	0	0	0	0	0	g	2	7,0	NA	Warmblood	1	350	3	cooperativeness	1	single	1	restricted pasture	1	restricted	1	restricted roughage	
305	gen.Germ.	quest	1	2452,0	unty itself	take off halter	2	NA		2	escape	0	1	0	0	0	0	0	g	2	8,0	hungarian warmblood	Warmblood	1	350	3	cooperativeness	2	group	2	unrestricted pasture	2	unrestricted contact	2	unrestrict roughage	
306	gen.Germ.	quest	1	2452,0	manipilate things	opens zippers and Velcro strips, play s with many different objects	2	NA		5	play	0	0	0	0	0	0	1	g	2	8,0	hungarian warmblood	Warmblood	1	350	3	cooperativeness	2	group	2	unrestricted pasture	2	unrestricted contact	2	unrestrict roughage	
307	gen.Germ.	quest	1	2470,0	open door	open door, open gate	1	more than 20 times	4	2	escape	0	1	0	0	0	0	0	g	2	10,0	Connemara	Pony	2	100	5	robustness	2	group	2	unrestricted pasture	2	unrestricted contact	2	unrestrict roughage	
308	gen.Engl.	quest	1	2265,0	open door	open slide door, to get out	4	2-10 times	2	2	escape	0	1	0	0	0	0	0	m	1	5,0	Quarter horse	Warmblood	1	350	3	cooperativeness	2	group	2	unrestricted pasture	2	unrestricted contact	2	unrestrict roughage	
309	gen.Engl.	quest	1	2265,0	open door	crouch underneath fence	4	2-10 times	2	2	escape	0	1	0	0	0	0	0	m	1	5,0	Quarter horse	Warmblood	1	350	3	cooperativeness	2	group	2	unrestricted pasture	2	unrestricted contact	2	unrestrict roughage	
310	gen.Engl.	quest	1	2265,0	open door	open slide door, to enter feed room	4	2-10 times	2	1	feeding	1	0	0	0	0	0	0	m	1	5,0	Quarter horse	Warmblood	1	350	3	cooperativeness	2	group	2	unrestricted pasture	2	unrestricted contact	2	unrestrict roughage	
311	gen.Engl.	quest	1	2265,0	open door	unty latches	4	2-10 times	2	2	escape	0	1	0	0	0	0	0	m	1	5,0	Quarter horse	Warmblood	1	350	3	cooperativeness	2	group	2	unrestricted pasture	2	unrestricted contact	2	unrestrict roughage	
312	gen.Engl.	quest	1	2272,0	open door	open latch of stall	1	NA		2	escape	0	1	0	0	0	0	0	g	2	10,0	Tennessee Walking	Warmblood	1	350	3	cooperativeness	1	single	2	unrestricted pasture	2	unrestricted contact	1	restricted roughage	
313	gen.Engl.	quest	1	2275,0	open door	open latch of stall and lets other horses out	1	more than 20 times	4	2	escape	0	1	0	0	0	0	0	m	1	10,0	Belgium / Tennessee Walking	Warmblood	1	350	3	cooperativeness	2	group	2	unrestricted pasture	2	unrestricted contact	2	unrestrict roughage	
314	gen.Engl.	quest	1	2279,0	open fence	escapes from stalls and pastures (technique unknown)	1	2-10 times	2	2	escape	0	1	0	0	0	0	0	g	2	18,0	Paint horse	Warmblood	1	350	3	cooperativeness	2	group	2	unrestricted pasture	2	unrestricted contact	2	unrestrict roughage	
315	gen.Engl.	quest	1	2342,0	open fence	ponie takes venyl fence down to escape when hay is empty	1	11-20 times	3	2	escape	0	1	0	0	0	0	0	g	2	9,0	Pony	Pony	2	100	5	robustness	2	group	2	unrestricted pasture	2	unrestricted contact	1	restricted roughage	
316	gen.Engl.	quest	1	2315,0	open door	opens and clothes doors, (see video), does not let others out, stays in the stable	1	more than 20 times	4	5	play	0	0	0	0	0	0	1	g	2	10,0	Tennessee Walking	Warmblood	1	350	3	cooperativeness	1	single	2	unrestricted pasture	2	unrestricted contact	1	restricted roughage	https://www.youtube.com/watch?v=qmJ9wbpPS
317	gen.Engl.	quest	1	2428,0	open door	open door	1	2-10 times	2	2	escape	0	1	0	0	0	0	0	g	2	4,0	Miniture horse	Pony	2	100	5	robustness	2	group	2	unrestricted pasture	2	unrestricted contact	2	unrestrict roughage	
318	gen.Engl.	quest	1	2439,0	open door	open gate	1	dayly	5	2	escape	0	1	0	0	0	0	0	g	2	4,0	Arabian	Arabian horse	3	4000	1	endurance	2	group	2	unrestricted pasture	2	unrestricted contact	2	unrestrict roughage	
319	gen.Engl.	quest	1	2444,0	manipulate things	takes rag down and throws it in fromt of persons feet when they enter the stable, person puts rag on	1	dayly	5	4	comfort	0	0	0	0	0	1	0	g	2	7,0	Thoroughbred	Thoroughbred	5	250	2	speed	1	single	2	unrestricted pasture	2	unrestricted contact	2	unrestrict roughage	
320	gen.Engl.	quest	1	2504,0	open fence	opens fence (technique unknown)	1	more than 20 times	4	2	escape	0	1	0	0	0	0	0	g	2	8,0	Arabian	Arabian horse	3	4000	1	endurance	2	group	2	unrestricted pasture	2	unrestricted contact	2	unrestrict roughage	
321	gen.Engl.	quest	1	2506,0	open door	breaks into feeder	1	2-10 times	2	1	feeding	1	0	0	0	0	0	0	m	1	13,0	Appaloosa	Warmblood	1	350	3	cooperativeness	2	group	2	unrestricted pasture	2	unrestricted contact	1	restricted roughage	
322	gen. French	quest	1	2502,0	feeding	soaks hay with water before feeding	1	more than 20 times	4	1	feeding	1	0	0	0	0	0	0	m	1	6,0	Selle Francais	Warmblood	1	350	3	cooperativeness	1	single	2	unrestricted pasture	2	unrestricted contact	1	restricted roughage	
323	gen. French	quest	1	2499,0	open door - gate	open door - gate	1	dayly	5	2	escape	0	1	0	0	0	0	0	g	2	18,0	Arabian	Arabian horse	3	4000	1	endurance	1	single	2	unrestricted pasture	2	unrestricted contact	2	unrestrict roughage	
324	gen. French	quest	1	2500,0	open door - gate	open door - gate	1	dayly	5	1	feeding	1	0	0	0	0	0	0	m	1	6,0	Connemara	Pony	2	100	5	robustness	1	single	2	unrestricted pasture	2	unrestricted contact	1	restricted roughage	
325	door_gate_Germ	video	3	2181,0	open door - gate	open door - gate	1	NA		2	escape	0	1	0	0	0	0	0	NA			NA	Warmblood	1	350	3	cooperativeness									https://www.youtube.com/results?q=clever+horse&sp=SCj
326	door_gate_Germ	video	3	2179,0	open door - gate	open door - gate, escapes	3	NA		2	escape	0	1	0	0	0	0	0	m	1		NA	Arabian horse	3	4000	1	endurance	1	single							https://www.youtube.com/watch?v=nDmEs_gRKgQ
327	door_gate_Germ	video	3	2179,0	open door - gate	open door - gate, enters other box and fee	3	NA		1	feeding	1	0	0	0	0	0	0	m	1		NA	Arabian horse	3	4000	1	endurance	1	single							https://www.youtube.com/watch?v=nDmEs_gRKgQ
328	door_gate_Germ	video	3	2179,0	open door - gate	open door - gate, frees other horse, enters	3	NA		3	social	0	0	0	1	1	0	0	m	1		NA	Arabian horse	3	4000	1	endurance	1	single							https://www.youtube.com/watch?v=nDmEs_gRKgQ
329	door_gate_Germ	video	3	2176,0	open door - gate	open door - gate, goes insight a box	1	NA		1	feeding	1	0	0	0	0	0	0	s	3		NA	Warmblood	1	350	3	cooperativeness	1	single							https://www.youtube.com/watch?v=LZ91lg3yZks
330	door_gate_Germ	video	3	2177,0	open door - gate	open door - gate	1	NA		2	escape	0	1	0	0	0	0	0	m	1		NA	Arabian horse	3	4000	1	endurance	1	single							https://www.youtube.com/watch?v=6pibfNWwVXg
331	door_gate_Germ	quest_doo	2	2184,0	open door - gate	open door - gate, escapes	3	more than 20 times	4	2	escape	0	1	0	0	0	0	0	g	2	14,0	NA	Draught horse	4	500	4	power	2	group	2	unrestricted pasture	2	unrestricted contact	1	restricted roughage	
332	door_gate_Germ	quest_doo	2	2184,0	open door - gate	open door - gate, enters other rooms	3	more than 20 times	4	1	feeding	1	0	0	0	0	0	0	g	2	14,0	NA	Draught horse	4	500	4	power	2	group	2	unrestricted pasture	2	unrestricted contact	1	restricted roughage	
333	door_gate_Germ	quest_doo	2	2184,0	unty_itself	unty_itself	3	more than 20 times	4	2	escape	0	1	0	0	0	0	0	g	2	14,0	NA	Draught horse	4	500	4	power	2	group	2	unrestricted pasture	2	unrestricted contact	1	restricted roughage	
334	door_gate_Germ	quest_doo	2	2187,0	open door - gate	open door - gate	1	more than 20 times	4	2	escape	0	1	0	0	0	0	0	g	2	5,0	NA	Warmblood	1	350	3	cooperativeness	1	single	2	unrestricted pasture	2	unrestricted contact	2	unrestrict roughage	
335	door_gate_Germ	quest_doo	2	2189,0	open door - gate	open door - gate, escapes	2	more than 20 times	4	2	escape	0	1	0	0	0	0	0	g	2	7,0	NA	Warmblood	1	350	3	cooperativeness	1	single	1	restricted pasture	2	unrestricted contact	2	unrestrict roughage	

1	sorce	video / quest	video_quest_num	sID	name behaviour	behaviour	nr reported behaviours	how often was the behaviour shown	behaviour frequency	behaviour category num	behaviour _category	feeding behaviour	escape behaviour	social behaviour human	social behaviour horse	social behaviour	comfort behaviour	unknown play	sex	sex num	age	breed	breed-type	breed type num	length of domestication	purpose of domestication	purpose of domestication	single group stabling	single group stabling	restricted unrestricted pasture	restricted unrestricted pasture	restricted unrestricted contact with horses	restricted unrestricted contact with horses	restricted unrestric. roughage	restricted unrestricted roughage	links
336	door_gate_Germ	quest_doo	2	2189,0	open door - gate	open door - gate, frees other horses	2	more than 20 times	4	3	social	0	0	0	1	1	0	0	g	2	7,0	NA	Warmblood	1	350	3	cooperativeness	1	single	1	restricted pasture	2	unrestricted contact	2	unrestrict roughage	
337	door_gate_Germ	quest_doo	2	2192,0	open door - gate	open door - gate	1	more than 20 times	4	2	escape	0	1	0	0	0	0	0	m	1	11,0	NA	Warmblood	1	350	3	cooperativeness	1	single	1	restricted pasture	1	restricted	1	restricted roughage	
338	door_gate_Germ	quest_doo	2	2195,0	open door - gate	open door - gate	1	more than 20 times	4	2	escape	0	1	0	0	0	0	0	g	2	24,0	NA	Warmblood	1	350	3	cooperativeness	2	group	1	restricted pasture	2	unrestricted contact	2	unrestrict roughage	
339	door_gate_Germ	quest_doo	2	2197,0	open door - gate	open door - gate	1	11-20 times	3	2	escape	0	1	0	0	0	0	0	s	3	24,0	NA	Warmblood	1	350	3	cooperativeness	1	single	1	restricted pasture	1	restricted	1	restricted roughage	
340	door_gate_Germ	quest_doo	2	2201,0	open door - gate	open door - gate	1	2-10 times	2	2	escape	0	1	0	0	0	0	0	g	2	5,0	NA	Warmblood	1	350	3	cooperativeness	1	single	1	restricted pasture	1	restricted	1	restricted roughage	
341	door_gate_Germ	quest_doo	2	2202,1	open door - gate	open door - gate, escapes	1	more than 20 times	4	2	escape	0	1	0	0	0	0	0	m	1	4,0	NA	Warmblood	1	350	3	cooperativeness	2	group	1	restricted pasture	2	unrestricted contact			
342	door_gate_Germ	video	3	2204,0	open door - gate	open door - gate	1	NA		2	escape	0	1	0	0	0	0	0	NA			NA	Warmblood	1	350	3	cooperativeness	1	single							https://www.youtube.com/watch?v=Y6580YcROyI
343	door_gate_Germ	video	3	2205,0	open door - gate	open door - gate	1	NA		1	feeding	1	0	0	0	0	0	0	NA			NA	Warmblood	1	350	3	cooperativeness									https://www.youtube.com/watch?v=lxMuQiFQN98
344	door_gate_Germ	quest_doo	2	2210,0	open door - gate	open door - gate	1	dayly	5	2	escape	0	1	0	0	0	0	0	g	2	18,0	NA	Pony	2	100	5	robustness	2	group	2	unrestricted pasture	2	unrestricted contact	2	unrestrict roughage	
345	door_gate_Germ	quest_doo	2	2212,0	open door - gate	open door - gate, escapes	3	more than 20 times	4	2	escape	0	1	0	0	0	0	0	g	2	3,0	NA	Warmblood	1	350	3	cooperativ	1	single	2	unrestricted	1	restricted	2	unrestrict	
346	door_gate_Germ	quest_doo	2	2212,0	open door - gate	open door - gate, frees other horses	3	more than 20 times	4	3	social	0	0	0	1	1	0	0	g	2	3,0	NA	Warmblood	1	350	3	cooperativ	1	single	2	unrestricted	1	restricted	2	unrestrict	
347	door_gate_Germ	quest_doo	2	2212,0	open door - gate	open door - gate, breakes into feed room	3	more than 20 times	4	1	feeding	1	0	0	0	0	0	0	g	2	3,0	NA	Warmblood	1	350	3	cooperativeness	1	single	2	unrestricted pasture	1	restricted	2	unrestrict roughage	
348	door_gate_Germ	quest_doo	2	2213,0	open door - gate	open door - gate	1	more than 20 times	4	2	escape	0	1	0	0	0	0	0	g	2	20,0	NA	Warmblood	1	350	3	cooperativeness	2	group	2	unrestricted pasture	2	unrestricted contact	2	unrestrict roughage	
349	door_gate_Germ	quest_doo	2	2215,0	open door - gate	open door - gate, escapes	4	11-20 times	3	2	escape	0	1	0	0	0	0	0	g	2	15,0	NA	Warmblood	1	350	3	cooperativeness	2	group	1	restricted pasture	2	unrestricted contact	2	unrestrict roughage	
350	door_gate_Germ	quest_doo	2	2215,0	open door - gate	open door - gate, lets others out	4	11-20 times	3	3	social	0	0	0	1	1	0	0	g	2	15,0	NA	Warmblood	1	350	3	cooperativeness	2	group	1	restricted pasture	2	unrestricted contact	2	unrestrict roughage	
351	door_gate_Germ	quest_doo	2	2215,0	open door - gate	open door - gate, opens lock(turning key)	4	11-20 times	3	5	play	0	0	0	0	0	0	1	g	2	15,0	NA	Warmblood	1	350	3	cooperativeness	2	group	1	restricted pasture	2	unrestricted contact	2	unrestrict roughage	
352	door_gate_Germ	quest_doo	2	2215,0	open door - gate	open gate, opens electric fence handles	4	11-20 times	3	2	escape	0	1	0	0	0	0	0	g	2	15,0	NA	Warmblood	1	350	3	cooperativeness	2	group	1	restricted pasture	2	unrestricted contact	2	unrestrict roughage	
353	door_gate_Germ	quest_doo	2	2224,0	open door - gate	open door - gate	2	more than 20 times	4	2	escape	0	1	0	0	0	0	0	m	1	10,0	NA	Warmblood	1	350	3	cooperativeness	1	single	1	restricted pasture	1	restricted	2	unrestrict roughage	
354	door_gate_Germ	quest_doo	2	2224,0	manipulate things	cose lock of trailor	2	more than 20 times	4	5	play	0	0	0	0	0	0	1	m	1	10,0	NA	Warmblood	1	350	3	cooperativeness	1	single	1	restricted pasture	1	restricted	2	unrestrict roughage	
355	door_gate_Germ	quest_doo	2	2227,0	open door - gate	open door - gate	1	11-20 times	3	2	escape	0	1	0	0	0	0	0	g	2	6,0	NA	Warmblood	1	350	3	cooperativeness	1	single	1	restricted pasture	1	restricted	1	restricted roughage	
356	door_gate_Germ	quest_doo	2	2228,0	open door - gate	open door - gate	1	11-20 times	3	2	escape	0	1	0	0	0	0	0	g	2	4,0	NA	Draught horse	4	500	4	power	1	single	1	restricted pasture	1	restricted	2	unrestrict roughage	
357	door_gate_Germ	quest_doo	2	2230,0	open door - gate	open door - gate, own door , stall door an	2	dayly	5	2	escape	0	1	0	0	0	0	0	m	1	10,0	NA	Warmblood	1	350	3	cooperativeness	2	group	2	unrestricted pasture	2	unrestricted contact	1	restricted roughage	
358	door_gate_Germ	quest_doo	2	2230,0	open door - gate	open door - gate, feed room door	2	dayly	5	1	feeding	1	0	0	0	0	0	0	m	1	10,0	NA	Warmblood	1	350	3	cooperativeness	2	group	2	unrestricted pasture	2	unrestricted contact	1	restricted roughage	
359	door_gate_Germ	quest_doo	2	2232,0	open door - gate	open door - gate, own door , stall door an	2	more than 20 times	4	1	feeding	1	0	0	0	0	0	0	m	1	18,0	NA	Warmblood	1	350	3	cooperativeness	2	group	2	unrestricted pasture	2	unrestricted contact	1	restricted roughage	
360	door_gate_Germ	quest_doo	2	2232,0	open door - gate	open door - gate, feed room door	2	more than 20 times	4	2	escape	0	1	0	0	0	0	0	m	1	18,0	NA	Warmblood	1	350	3	cooperativeness	2	group	2	unrestricted pasture	2	unrestricted contact	1	restricted roughage	
361	door_gate_Germ	quest_doo	2	2234,0	open door - gate	open door - gate	1	2-10 times	2	2	escape	0	1	0	0	0	0	0	g	2	1,0	NA	Warmblood	1	350	3	cooperativeness	2	group	1	restricted pasture	2	unrestricted contact	2	unrestrict roughage	
362	door_gate_Germ	quest_doo	2	2236,0	open door - gate	open door - gate	1	NA		2	escape	0	1	0	0	0	0	0	g	2	14,0	NA	Warmblood	1	350	3	cooperativeness	1	single	2	unrestricted pasture	1	restricted	1	restricted roughage	
363	door_gate_Germ	quest_doo	2	2237,0	open door - gate	open door - gate, own door , stall door an	2	dayly	5	2	escape	0	1	0	0	0	0	0	g	2	17,0	NA	Warmblood	1	350	3	cooperativeness	1	single	2	unrestricted pasture	1	restricted	1	restricted roughage	
364	door_gate_Germ	quest_doo	2	2237,0	open door - gate	open door - gate, frees other horses	2	dayly	5	3	social	0	0	0	1	1	0	0	g	2	17,0	NA	Warmblood	1	350	3	cooperativeness	1	single	2	unrestricted pasture	1	restricted	1	restricted roughage	
365	door_gate_Germ	quest_doo	2	2239,0	open door - gate	open door - gate	1	more than 20 times	4	2	escape	0	1	0	0	0	0	0	g	2	8,0	NA	Warmblood	1	350	3	cooperativeness	1	single	2	unrestricted pasture	1	restricted	2	unrestrict roughage	
366	door_gate_Germ	quest_doo	2	2240,0	open door - gate	open door - gate	1	more than 20 times	4	2	escape	0	1	0	0	0	0	0	g	2	7,0	NA	Pony	2	100	5	robustnes	2	group			2	unrestricted	1	restricted	
367	door_gate_Germ	quest_doo	2	2241,0	open door - gate	open door - gate	1	more than 20 times	4	2	escape	0	1	0	0	0	0	0	m	1	19,0	NA	Warmblood	1	350	3	cooperativeness	1	single	2	unrestricted pasture	1	restricted	1	restricted roughage	
368	door_gate_Germ	quest_doo	2	2244,0	open door - gate	open door - gate, own door , stall door an	3	more than 20 times	4	2	escape	0	1	0	0	0	0	0	m	1	21,0	NA	Warmblood	1	350	3	cooperativeness	1	single	2	unrestricted pasture	1	restricted	1	restricted roughage	
369	door_gate_Germ	quest_doo	2	2244,0	open door - gate	open door - gate, feed room door	3	more than 20 times	4	1	feeding	1	0	0	0	0	0	0	m	1	21,0	NA	Warmblood	1	350	3	cooperativeness	1	single	2	unrestricted pasture	1	restricted	1	restricted roughage	
370	door_gate_Germ	quest_doo	2	2244,0	open door - gate	open door - gate, frees other horses	3	more than 20 times	4	3	social	0	0	0	1	1	0	0	m	1	21,0	NA	Warmblood	1	350	3	cooperativeness	1	single	2	unrestricted pasture	1	restricted	1	restricted roughage	

1	sorce	video / quest	video_quest_num	sID	name behaviour	behaviour	nr reported behaviours	how often was the behaviour shown	behaviour frequency	behaviour category num	behaviour_category	feeding behaviour	escape behaviour	social behaviour human	social behaviour horse	social behaviour	comfort behaviour	unknown play	sex	sex num	age	breed	breed-type	breed type num	length of domestication	purpose of domestication	purpose of domestication	single group stabling	single group stabling	restric unrest d past	[illegible]	[illegible]	[illegible]	[illegible]	[illegible]	links
371	door_gate_Germ	quest_doo	2	2246,0	open door - gate	open door - gate	1	more than 20 times	4	2	escape	0	1	0	0	0	0	0	g	2	6,0	NA	Warmblood	1	350	3	cooperativeness	2	group	[illegible]	[illegible]	[illegible]	[illegible]	[illegible]	[illegible]	
372	door_gate_Germ	quest_doo	2	2249,0	open door - gate	open door - gate	1	2-10 times	2	2	escape	0	1	0	0	0	0	0	m	1	17,0	NA	Warmblood	1	350	3	cooperativeness	2	group	[illegible]	[illegible]	[illegible]	[illegible]	[illegible]	[illegible]	
373	door_gate_Germ	quest_doo	2	2252,0	open door - gate	open door - gate	1	dayly	5	2	escape	0	1	0	0	0	0	0	g	2	5,0	NA	Warmblood	1	350	3	cooperativeness	2	group	[illegible]	[illegible]	[illegible]	[illegible]	[illegible]	[illegible]	
374	door_gate_Germ	quest_doo	2	2255,0	open door - gate	open door - gate	1	2-10 times	2	5	play	0	0	0	0	0	0	1	g	2	7,0	NA	Warmblood	1	350	3	cooperativeness	1	single	[illegible]	[illegible]	[illegible]	[illegible]	[illegible]	[illegible]	
375	door_gate_Germ	quest_doo	2	2256,0	open door - gate	open door - gate	1	more than 20 times	4	2	escape	0	1	0	0	0	0	0	s	3	3,0	NA	Draught horse	4	500	4	power	2	group	2	[illegible] pasture	2	[illegible] contact	2	[illegible] roughage	
376	door_gate_Germ	quest_doo	2	2257,0	open door - gate	open door - gate	1	NA		2	escape	0	1	0	0	0	0	0	g	2	5,0	NA	Warmblood	1	350	3	cooperativeness	2	group	2	unrestricted pasture	2	unrestricted contact	2	unrestrict roughage	
377	door_gate_Germ	quest_doo	2	2264,0	open door - gate	open door - gate, own door , stall door an	2	dayly	5	2	escape	0	1	0	0	0	0	0	m	1	4,0	NA	Arabian horse	3	4000	1	endurance	2	group	2	unrestricted pasture	2	unrestricted contact	1	restricted roughage	
378	door_gate_Germ	quest_doo	2	2264,0	open door - gate	open door - gate, feed room door	2	dayly	5	1	feeding	1	0	0	0	0	0	0	m	1	4,0	NA	Arabian horse	3	4000	1	endurance	2	group	2	unrestricted pasture	2	unrestricted contact	1	restricted roughage	
379	door_gate_Germ	quest_doo	2	2280,0	open door - gate	open door - gate, own door , stall door an	3	more than 20 times	4	2	escape	0	1	0	0	0	0	0	g	2	12,0	NA	Warmblood	1	350	3	cooperativ	2	group	2	unrestricted	2	unrestricted	2	unrestrict	
380	door_gate_Germ	quest_doo	2	2280,0	open door - gate	open door - gate, feed room door	3	more than 20 times	4	1	feeding	1	0	0	0	0	0	0	g	2	12,0	NA	Warmblood	1	350	3	cooperativeness	2	group	2	unrestricted pasture	2	unrestricted contact	2	unrestrict roughage	
381	door_gate_Germ	quest_doo	2	2280,0	open door - gate	open door - gate, frees other horses	3	more than 20 times	4	3	social	0	0	0	1	1	0	0	g	2	12,0	NA	Warmblood	1	350	3	cooperativeness	2	group	2	unrestricted pasture	2	unrestricted contact	2	unrestrict roughage	
382	door_gate_Germ	quest_doo	2	2284,0	open door - gate	open door - gate	1	2-10 times	2	2	escape	0	1	0	0	0	0	0	g	2	11,0	NA	Warmblood	1	350	3	cooperativeness	1	single	2	unrestricted pasture	1	restricted	1	restricted roughage	
383	door_gate_Germ	quest_doo	2	2286,0	open door - gate	open door - gate	1	dayly	5	2	escape	0	1	0	0	0	0	0	g	2	10,0	NA	Warmblood	1	350	3	cooperativeness	2	group	2	unrestricted pasture	2	unrestricted contact	1	restricted roughage	
384	door_gate_Germ	quest_doo	2	2287,0	open door - gate	open door - gate	1	more than 20 times	4	2	escape	0	1	0	0	0	0	0	g	2	10,0	NA	Pony	2	100	5	robustness	2	group	2	unrestricted pasture	2	unrestricted contact	1	restricted roughage	
385	door_gate_Germ	quest_doo	2	2289,0	open door - gate	open door - gate, own door , stall door an	2	more than 20 times	4	2	escape	0	1	0	0	0	0	0	g	2	8,0	NA	Draught horse	4	500	4	power	1	single			1	restricted	2	unrestrict roughage	
386	door_gate_Germ	quest_doo	2	2289,0	open door - gate	open door - gate, frees other horses	2	more than 20 times	4	3	social	0	0	0	1	1	0	0	g	2	8,0	NA	Draught horse	4	500	4	power	1	single			1	restricted	2	unrestrict roughage	
387	door_gate_Germ	quest_doo	2	2290,0	open door - gate	open door - gate, own door , stall door an	3	more than 20 times	4	2	escape	0	1	0	0	0	0	0	g	2	12,0	NA	Warmblood	1	350	3	cooperativeness	1	single	1	restricted pasture	1	restricted	2	unrestrict roughage	
388	door_gate_Germ	quest_doo	2	2290,0	open door - gate	open door - gate, frees other horses	3	more than 20 times	4	3	social	0	0	0	1	1	0	0	g	2	12,0	NA	Warmblood	1	350	3	cooperativeness	1	single	1	restricted pasture	1	restricted	2	unrestrict roughage	
389	door_gate_Germ	quest_doo	2	2290,0	manipulate_things	turn light switch	3	more than 20 times	4	5	play	0	0	0	0	0	0	1	g	2	12,0	NA	Warmblood	1	350	3	cooperativeness	1	single	1	restricted pasture	1	restricted	2	unrestrict roughage	
390	door_gate_Germ	quest_doo	2	2291,0	open door - gate	open door - gate	1	more than 20 times	4	2	escape	0	1	0	0	0	0	0	g	2	28,0	NA	Warmblood	1	350	3	cooperativeness	2	group	2	unrestricted pasture	2	unrestricted contact	1	restricted roughage	
391	door_gate_Germ	quest_doo	2	2292,0	open door - gate	open door - gate	2	2-10 times	2	2	escape	0	1	0	0	0	0	0	g	2	12,0	NA	Warmblood	1	350	3	cooperativeness	2	group	2	unrestricted pasture	2	unrestricted contact	2	unrestrict roughage	
392	door_gate_Germ	quest_doo	2	2292,0	unty itself	unty itself	2	2-10 times	2	2	escape	0	1	0	0	0	0	0	g	2	12,0	NA	Warmblood	1	350	3	cooperativeness	2	group	2	unrestricted pasture	2	unrestricted contact	2	unrestrict roughage	
393	door_gate_Germ	quest_doo	2	2293,0	open door - gate	open door - gate	2	11-20 times	3	5	play	0	0	0	0	0	0	1	g	2	16,0	NA	Warmblood	1	350	3	cooperativeness	1	single	1	restricted pasture	1	restricted	1	restricted roughage	
394	door_gate_Germ	quest_doo	2	2293,0	manipulate_things	open fence	2	11-20 times	3	2	escape	0	1	0	0	0	0	0	g	2	16,0	NA	Warmblood	1	350	3	cooperativeness	1	single	1	restricted pasture	1	restricted	1	restricted roughage	
395	door_gate_Germ	quest_doo	2	2294,0	open door - gate	open door - gate, own door , stall door an	2	2-10 times	2	2	escape	0	1	0	0	0	0	0	g	2	10,0	NA	NA					1	single	1	restricted pasture	1	restricted	1	restricted roughage	
396	door_gate_Germ	quest_doo	2	2294,0	open door - gate	open door - gate, frees other horses	2	2-10 times	2	3	social	0	0	0	1	1	0	0	g	2	10,0	NA	NA					1	single	1	restricted pasture	1	restricted	1	restricted roughage	
397	door_gate_Germ	quest_doo	2	2295,1	open door - gate	open door - gate, own door , stall door an	2	more than 20 times	4	2	escape	0	1	0	0	0	0	0	g	2	11,0	NA	Warmblood	1	350	3	cooperativeness	1	single	1	restricted pasture	1	restricted	1	restricted roughage	
398	door_gate_Germ	quest_doo	2	2295,1	open door - gate	open door - gate, frees other horses	2	more than 20 times	4	3	social	0	0	0	1	1	0	0	g	2	11,0	NA	Warmblood	1	350	3	cooperativeness	1	single	1	restricted pasture	1	restricted	1	restricted roughage	
399	door_gate_Germ	quest_doo	2	2296,0	open door - gate	open door - gate	1	2-10 times	2	2	escape	0	1	0	0	0	0	0	m	1	5,0	NA	Warmblood	1	350	3	cooperativeness	2	group	1	restricted pasture	2	unrestricted contact	1	restricted roughage	
400	door_gate_Germ	quest_doo	2	2298,0	open door - gate	open door - gate	1	dayly	5	2	escape	0	1	0	0	0	0	0	g	2	4,0	NA	Warmblood	1	350	3	cooperativeness	2	group	2	unrestricted pasture	2	unrestricted contact	1	restricted roughage	
401	door_gate_Germ	quest_doo	2	2301,0	open door - gate	open door - gate	1	more than 20 times	4	2	escape	0	1	0	0	0	0	0	g	2	15,0	NA	Arabian horse	3	4000	1	endurance	2	group	1	restricted	2	unrestricted	1	restricted	
402	door_gate_Germ	quest_doo	2	2306,0	open door - gate	open door - gate	1	2-10 times	2	2	escape	0	1	0	0	0	0	0	g	2	8,0	NA	Draught horse	4	500	4	power	2	group	2	unrestricted pasture	2	unrestricted contact	1	restricted roughage	
403	door_gate_Germ	quest_doo	2	2307,0	open door - gate	open door - gate, own door , stall door an	3	2-10 times	2	2	escape	0	1	0	0	0	0	0	g	2	15,0	NA	Pony	2	100	5	robustness	2	group	2	unrestricted pasture	2	unrestricted contact	2	unrestrict roughage	
404	door_gate_Germ	quest_doo	2	2307,0	open door - gate	open door - gate, feed room door	3	2-10 times	2	1	feeding	1	0	0	0	0	0	0	g	2	15,0	NA	Pony	2	100	5	robustness	2	group	2	unrestricted pasture	2	unrestricted contact	2	unrestrict roughage	
405	door_gate_Germ	quest_doo	2	2307,0	manipulate things	open fence	3	2-10 times	2	2	escape	0	1	0	0	0	0	0	g	2	15,0	NA	Pony	2	100	5	robustness	2	group	2	unrestricted pasture	2	unrestricted contact	2	unrestrict roughage	

in der Arbeitsmappe löschen.

Info: Wenn Sie gleichzeitig mehrere Zeilen oder Spalten löschen möchten, wählen Sie diese Zeilen oder Spalten im Blatt aus, und klicken Sie dann auf "Löschen".

1	sorce	video / quest	video_quest_num	sID	name behaviour	behaviour	nr reported behaviours	how often was the behaviour shown	behaviour frequency	behaviour category num	behaviour _category	feeding behaviour	escape behaviour	social behaviour human	social behaviour horse	social behaviour	comfort behaviour	unknown play	sex	sex num	age	breed	breed-type	breed type num	length of domestication	purpose of domestication	purpose of domestication	single group stabling	single group stabling	restricted unrestricted pasture	restricted unrestricted pasture	restricted unrestricted contact with horses	restricted unrestricted contact with horses	restricted unrestric. roughage	restricted unrestricted roughage	links
406	door_gate_Germ	quest_doo	2	2309,0	open door - gate	open door - gate, own door , stall door an	2	more than 20 times	4	2	escape	0	1	0	0	0	0	0	m	1	10,0	NA	Warmblood	1	350	3	cooperativeness	1	single			1	restricted	2	unrestrict roughage	
407	door_gate_Germ	quest_doo	2	2309,0	open door - gate	open door - gate, feed room door	2	more than 20 times	4	1	feeding	1	0	0	0	0	0	0	m	1	10,0	NA	Warmblood	1	350	3	cooperativeness	1	single			1	restricted	2	unrestrict roughage	
408	door_gate_Germ	quest_doo	2	2310,0	open door - gate	open door - gate, opens own stable door t	2	dayly	5	1	feeding	1	0	0	0	0	0	0	m	1	2,0	NA	Warmblood	1	350	3	cooperativeness	2	group	2	unrestricted pasture	2	unrestricted contact	2	unrestrict roughage	
409	door_gate_Germ	quest_doo	2	2310,0	open door - gate	open door - gate, fress other horses	2	dayly	5	3	social	0	0	0	1	1	0	0	m	1	2,0	NA	Warmblood	1	350	3	cooperativeness	2	group	2	unrestricted pasture	2	unrestricted contact	2	unrestrict roughage	
410	door_gate_Germ	quest_doo	2	2314,0	open door - gate	open door - gate, own door , stall door an	6	2-10 times	2	2	escape	0	1	0	0	0	0	0	g	2	3,0	NA	Pony	2	100	5	robustness	2	group	2	unrestricted pasture	1	restricted	2	unrestrict roughage	
411	door_gate_Germ	quest_doo	2	2314,0	open door - gate	open door - gate, feed room door	6	2-10 times	2	1	feeding	1	0	0	0	0	0	0	g	2	3,0	NA	Pony	2	100	5	robustness	2	group	2	unrestricted pasture	1	restricted	2	unrestrict roughage	
412	door_gate_Germ	quest_doo	2	2314,0	open door - gate	open door - gate, free other horses	6	2-10 times	2	3	social	0	0	0	1	1	0	0	g	2	3,0	NA	Pony	2	100	5	robustness	2	group	2	unrestricted pasture	1	restricted	2	unrestrict roughage	
413	door_gate_Germ	quest_doo	2	2314,0	manipulate_things	unty itself	6	2-10 times	2	2	escape	0	1	0	0	0	0	0	g	2	3,0	NA	Pony	2	100	5	robustness	2	group	2	unrestricted pasture	1	restricted	2	unrestrict roughage	
414	door_gate_Germ	quest_doo	2	2314,0	manipulate_things	unty others	6	2-10 times	2	3	social	0	0	0	1	1	0	0	g	2	3,0	NA	Pony	2	100	5	robustness	2	group	2	unrestricted pasture	1	restricted	2	unrestrict roughage	
415	door_gate_Germ	quest_doo	2	2314,0	manipulate_things	carries objects	6	2-10 times	2	5	play	0	0	0	0	0	0	1	g	2	3,0	NA	Pony	2	100	5	robustness	2	group	2	unrestricted pasture	1	restricted	2	unrestrict roughage	
416	door_gate_Germ	quest_doo	2	2316,0	open door - gate	open door - gate	1	more than 20 times	4	2	escape	0	1	0	0	0	0	0	g	2	10,0	NA	Draught horse	4	500	4	power	2	group	2	unrestricted pasture	2	unrestricted contact	1	restricted roughage	
417	door_gate_Germ	quest_doo	2	2318,0	open door - gate	open door - gate	1	2-10 times	2	2	escape	0	1	0	0	0	0	0	g	2	10,0	NA	Warmblood	1	350	3	cooperativeness	1	single	1	restricted pasture	1	restricted	1	restricted roughage	
418	door_gate_Germ	quest_doo	2	2319,0	open door - gate	open door - gate	1	dayly	5	2	escape	0	1	0	0	0	0	0	g	2	4,0	NA	Warmblood	1	350	3	cooperativeness	1	single	1	restricted pasture	1	restricted	2	unrestrict roughage	
419	door_gate_Germ	quest_doo	2	2320,0	open door - gate	open door - gate	1	more than 20 times	4	2	escape	0	1	0	0	0	0	0	s	3	27,0	NA	NA					2	group	1	restricted	2	unrestricted	1	restricted	
420	door_gate_Germ	quest_doo	2	2322,0	open door - gate	open door - gate	2	2-10 times	2	2	escape	0	1	0	0	0	0	0	g	2	6,0	NA	Warmblood	1	350	3	cooperativeness	2	group	2	unrestricted pasture	2	unrestricted contact	1	restricted roughage	
421	door_gate_Germ	quest_doo	2	2322,0	manipuilate things	turn water clock	2	2-10 times	2	1	feeding	1	0	0	0	0	0	0	g	2	6,0	NA	Warmblood	1	350	3	cooperativeness	2	group	2	unrestricted pasture	2	unrestricted contact	1	restricted roughage	
422	door_gate_Germ	quest_doo	2	2323,0	open door - gate	open door - gate	3	11-20 times	3	5	play	0	0	0	0	0	0	1	m	1	9,0	NA	Warmblood	1	350	3	cooperativeness	1	single	1	restricted pasture	1	restricted	1	restricted roughage	
423	door_gate_Germ	quest_doo	2	2323,0	manipulate things	open water clock	3	11-20 times	3	1	feeding	1	0	0	0	0	0	0	m	1	9,0	NA	Warmblood	1	350	3	cooperativeness	1	single	1	restricted pasture	1	restricted	1	restricted roughage	
424	door_gate_Germ	quest_doo	2	2323,0	manipulate things	unty itself	3	11-20 times	3	2	escape	0	1	0	0	0	0	0	m	1	9,0	NA	Warmblood	1	350	3	cooperativeness	1	single	1	restricted pasture	1	restricted	1	restricted roughage	
425	door_gate_Germ	quest_doo	2	2324,0	open door - gate	open door - gate	1	11-20 times	3	2	escape	0	1	0	0	0	0	0	g	2	10,0	NA	Thoroughbred	5	250	2	speed	1	single	1	restricted pasture	1	restricted			
426	door_gate_Germ	quest_doo	2	2329,0	open door - gate	open door - gate	1	dayly	5	2	escape	0	1	0	0	0	0	0	g	2	15,0	NA	Warmblood	1	350	3	cooperativeness	2	group	1	restricted pasture	2	unrestricted contact	1	restricted roughage	
427	door_gate_Germ	quest_doo	2	2332,0	open door - gate	open door - gate, own door , stall door an	2	2-10 times	2	2	escape	0	1	0	0	0	0	0	m	1	14,0	NA	NA					1	single	2	unrestricted pasture	1	restricted	1	restricted roughage	
428	door_gate_Germ	quest_doo	2	2332,0	open door - gate	open door - gate, feed room door	2	2-10 times	2	1	feeding	1	0	0	0	0	0	0	m	1	14,0	NA	NA					1	single	2	unrestricted pasture	1	restricted	1	restricted roughage	
429	door_gate_Germ	quest_doo	2	2333,0	open door - gate	open door - gate	1	NA		1	feeding	1	0	0	0	0	0	0	g	2	10,0	NA	Pony	2	100	5	robustness	2	group	2	unrestricted pasture	2	unrestricted contact	1	restricted roughage	
430	door_gate_Germ	quest_doo	2	2334,0	open door - gate	open door - gate, own door , stall door an	3	2-10 times	2	2	escape	0	1	0	0	0	0	0	m	1	11,0	NA	Warmblood	1	350	3	cooperativeness	1	single	1	restricted pasture	1	restricted	2	unrestrict roughage	
431	door_gate_Germ	quest_doo	2	2334,0	open door - gate	open door - gate, feed room door	3	2-10 times	2	1	feeding	1	0	0	0	0	0	0	m	1	11,0	NA	Warmblood	1	350	3	cooperativeness	1	single	1	restricted pasture	1	restricted	2	unrestrict roughage	
432	door_gate_Germ	quest_doo	2	2334,0	open door - gate	open door - gate, frees other horses	3	2-10 times	2	3	social	0	0	0	1	1	0	0	m	1	11,0	NA	Warmblood	1	350	3	cooperativeness	1	single	1	restricted pasture	1	restricted	2	unrestrict roughage	
433	door_gate_Germ	quest_doo	2	2335,0	open door - gate	open door - gate	1	more than 20 times	4	2	escape	0	1	0	0	0	0	0	m	1	5,0	NA	Draught horse	4	500	4	power	1	single	2	unrestricted pasture	1	restricted	1	restricted roughage	
434	door_gate_Germ	quest_doo	2	2336,0	open door - gate	open door - gate	2	2-10 times	2	2	escape	0	1	0	0	0	0	0	s	3	4,0	NA	Warmblood	1	350	3	cooperativeness	2	group	2	unrestricted pasture	2	unrestricted contact	2	unrestrict roughage	
435	door_gate_Germ	quest_doo	2	2336,0	maniplate things	unty itself	2	2-10 times	2	2	escape	0	1	0	0	0	0	0	s	3	4,0	NA	Warmblood	1	350	3	cooperativeness	2	group	2	unrestricted pasture	2	unrestricted contact	2	unrestrict roughage	
436	door_gate_Germ	quest_doo	2	2338,0	open door - gate	open door - gate	1	2-10 times	2	2	escape	0	1	0	0	0	0	0	g	2	7,0	NA	Warmblood	1	350	3	cooperativeness	1	single	1	restricted pasture	1	restricted	2	unrestrict roughage	
437	door_gate_Germ	quest_doo	2	2341,0	open door - gate	open door - gate, own door , stall door an	2	dayly	5	2	escape	0	1	0	0	0	0	0	m	1	6,0	NA	Warmblood	1	350	3	cooperativeness	2	group	2	unrestricted pasture	2	unrestricted contact	2	unrestrict roughage	
438	door_gate_Germ	quest_doo	2	2341,0	open door - gate	open door - gate, frees other horses	2	dayly	5	3	social	0	0	0	1	1	0	0	m	1	6,0	NA	Warmblood	1	350	3	cooperativeness	2	group	2	unrestricted pasture	2	unrestricted contact	2	unrestrict roughage	
439	door_gate_Germ	quest_doo	2	2343,0	open door - gate	open door - gate	1	2-10 times	2	2	escape	0	1	0	0	0	0	0	s	3	22,0	NA	Warmblood	1	350	3	cooperativeness	1	single	1	restricted pasture	1	restricted	2	unrestrict roughage	
440	door_gate_Germ	quest_doo	2	2340,0	open door - gate	open door - gate	2	more than 20 times	4	2	escape	0	1	0	0	0	0	0	g	2	3,0	NA	Warmblood	1	350	3	cooperativeness	2	group	2	unrestricted pasture	2	unrestricted contact	2	unrestrict roughage	

1	sorce	video / quest	video_quest_num	sID	name behaviour	behaviour	nr reported behaviours	how often was the behaviour shown	behaviour frequency	behaviour category num	behaviour_category	feeding behaviour	escape behaviour	social behaviour human	social behaviour horse	social behaviour	comfort behaviour	unknown play	sex	sex num	age	breed	breed-type	breed type num	length of domestication	purpose of domestication	purpose of domestication	single group stabling	single group stabling	restricted unrestricted pasture	restricted unrestricted pasture	restricted unrestricted contact with horses	restricted unrestricted contact with horses	restricted unrestric. roughage	restricted unrestricted roughage	links
441	door_gate_Germ	quest_doo	2	2340,0	manipulate things	open water clock	2	more than 20 times	4	1	feeding	1	0	0	0	0	0	0	g	2	3,0	NA	Warmblood	1	350	3	cooperativeness	2	group	2	unrestricted pasture	2	unrestricted contact	2	unrestrict roughage	
442	door_gate_Germ	quest_doo	2	2344,0	open door - gate	open door - gate	2	2-10 times	2	2	escape	0	1	0	0	0	0	0	g	2	6,0	NA	Warmblood	1	350	3	cooperativeness	1	single	1	restricted pasture	1	restricted	2	unrestrict roughage	
443	door_gate_Germ	quest_doo	2	2344,0	unty itself	unty itself	2	2-10 times	2	2	escape	0	1	0	0	0	0	0	g	2	6,0	NA	Warmblood	1	350	3	cooperativeness	1	single	1	restricted pasture	1	restricted	2	unrestrict roughage	
444	door_gate_Germ	quest_doo	2	2345,0	open door - gate	open door - gate, own door , stall door an	2	dayly	5	2	escape	0	1	0	0	0	0	0	m	1	10,0	NA	Warmblood	1	350	3	cooperativeness	2	group	2	unrestricted pasture	2	unrestricted contact	1	restricted roughage	
445	door_gate_Germ	quest_doo	2	2345,0	open door - gate	open door - gate, feed room door	2	dayly	5	1	feeding	1	0	0	0	0	0	0	m	1	10,0	NA	Warmblood	1	350	3	cooperativeness	2	group	2	unrestricted pasture	2	unrestricted contact	1	restricted roughage	
446	door_gate_Germ	quest_doo	2	2346,0	open door - gate	open door - gate, own door , stall door an	2	more than 20 times	4	2	escape	0	1	0	0	0	0	0	g	2	18,0	NA	Warmblood	1	350	3	cooperativeness	2	group	2	unrestricted pasture	2	unrestricted contact	2	unrestrict roughage	
447	door_gate_Germ	quest_doo	2	2346,0	open door - gate	open door - gate, frees other horses	2	more than 20 times	4	3	social	0	0	0	1	1	0	0	g	2	18,0	NA	Warmblood	1	350	3	cooperativeness	2	group	2	unrestricted pasture	2	unrestricted contact	2	unrestrict roughage	
448	door_gate_Germ	quest_doo	2	2350,0	open door - gate	open door - gate, own door , stall door an	3	2-10 times	2	2	escape	0	1	0	0	0	0	0	g	2	21,0	NA	NA					1	single	1	restricted pasture	1	restricted	1	restricted roughage	
449	door_gate_Germ	quest_doo	2	2350,0	open door - gate	open door - gate, frees other horses	3	2-10 times	2	3	social	0	0	0	1	1	0	0	g	2	21,0	NA	NA					1	single	1	restricted pasture	1	restricted	1	restricted roughage	
450	door_gate_Germ	quest_doo	2	2350,0	unty itself	unty itself	3	2-10 times	2	2	escape	0	1	0	0	0	0	0	g	2	21,0	NA	NA					1	single	1	restricted pasture	1	restricted	1	restricted roughage	
451	door_gate_Germ	quest_doo	2	2356,0	open door - gate	open door - gate	1	2-10 times	2	2	escape	0	1	0	0	0	0	0	m	1	18,0	NA	Pony	2	100	5	robustnes	2	group	1	restricted	2	unrestricted	1	restricted	
452	door_gate_Germ	quest_doo	2	2357,0	open door - gate	open door - gate, own door , stall door an	3	2-10 times	2	2	escape	0	1	0	0	0	0	0	g	2	9,0	NA	Warmblood	1	350	3	cooperativeness	2	group	2	unrestricted pasture	2	unrestricted contact	1	restricted roughage	
453	door_gate_Germ	quest_doo	2	2357,0	open door - gate	open door - gate, feed room door	3	2-10 times	2	1	feeding	1	0	0	0	0	0	0	g	2	9,0	NA	Warmblood	1	350	3	cooperativeness	2	group	2	unrestricted pasture	2	unrestricted contact	1	restricted roughage	
454	door_gate_Germ	quest_doo	2	2357,0	open door - gate	open door - gate, frees other horses	3	2-10 times	2	3	social	0	0	0	1	1	0	0	g	2	9,0	NA	Warmblood	1	350	3	cooperativeness	2	group	2	unrestricted pasture	2	unrestricted contact	1	restricted roughage	
455	door_gate_Germ	quest_doo	2	2358,0	open door - gate	open door - gate, own door , stall door an	2	11-20 times	3	2	escape	0	1	0	0	0	0	0	g	2	4,0	NA	Draught horse	4	500	4	power	1	single	1	restricted pasture	1	restricted	1	restricted roughage	
456	door_gate_Germ	quest_doo	2	2358,0	open door - gate	open door - gate, feed room door	2	11-20 times	3	1	feeding	1	0	0	0	0	0	0	g	2	4,0	NA	Draught horse	4	500	4	power	1	single	1	restricted pasture	1	restricted	1	restricted roughage	
457	door_gate_Germ	quest_doo	2	2361,0	open door - gate	open door - gate, own door , stall door an	3	11-20 times	3	2	escape	0	1	0	0	0	0	0	g	2	5,0	NA	Draught horse	4	500	4	power	2	group	2	unrestricted pasture	2	unrestricted contact	2	unrestrict roughage	
458	door_gate_Germ	quest_doo	2	2361,0	open door - gate	open door - gate, feed room door	3	11-20 times	3	1	feeding	1	0	0	0	0	0	0	g	2	5,0	NA	Draught horse	4	500	4	power	2	group	2	unrestricted pasture	2	unrestricted contact	2	unrestrict roughage	
459	door_gate_Germ	quest_doo	2	2361,0	open door - gate	open door - gate, opens and closes doors	3	11-20 times	3	5	play	0	0	0	0	0	0	1	g	2	5,0	NA	Draught horse	4	500	4	power	2	group	2	unrestricted pasture	2	unrestricted contact	2	unrestrict roughage	
460	door_gate_Germ	quest_doo	2	2363,0	open door - gate	open door - gate	1	more than 20 times	4	2	escape	0	1	0	0	0	0	0	g	2	10,0	NA	Warmblood	1	350	3	cooperativeness	1	single	1	restricted pasture	1	restricted	1	restricted roughage	
461	door_gate_Germ	quest_doo	2	2364,0	open door - gate	open door - gate, own door , stall door an	3	dayly	5	2	escape	0	1	0	0	0	0	0	g	2	7,0	NA	Warmblood	1	350	3	cooperativeness	2	group	2	unrestricted pasture	2	unrestricted contact	2	unrestrict roughage	
462	door_gate_Germ	quest_doo	2	2364,0	open door - gate	open door - gate, feed room door	3	dayly	5	1	feeding	1	0	0	0	0	0	0	g	2	7,0	NA	Warmblood	1	350	3	cooperativeness	2	group	2	unrestricted pasture	2	unrestricted contact	2	unrestrict roughage	
463	door_gate_Germ	quest_doo	2	2364,0	open door - gate	open door - gate, frees other horses	3	dayly	5	3	social	0	0	0	1	1	0	0	g	2	7,0	NA	Warmblood	1	350	3	cooperativeness	2	group	2	unrestricted pasture	2	unrestricted contact	2	unrestrict roughage	
464	door_gate_Germ	quest_doo	2	2365,0	open door - gate	open door - gate	1	more than 20 times	4	2	escape	0	1	0	0	0	0	0	g	2	4,0	NA	Arabian horse	3	4000	1	endurance	2	group	1	restricted	2	unrestricted	1	restricted	
465	door_gate_Germ	quest_doo	2	2366,0	open door - gate	open door - gate, own door , stall door an	2	2-10 times	2	2	escape	0	1	0	0	0	0	0	m	1	9,0	NA	Warmblood	1	350	3	cooperativeness	1	single	2	unrestricted pasture	1	restricted	2	unrestrict roughage	
466	door_gate_Germ	quest_doo	2	2366,0	open door - gate	open door - gate, frees other horses	2	2-10 times	2	3	social	0	0	0	1	1	0	0	m	1	9,0	NA	Warmblood	1	350	3	cooperativeness	1	single	2	unrestricted pasture	1	restricted	2	unrestrict roughage	
467	door_gate_Germ	quest_doo	2	2367,0	open door - gate	open door - gate, own door , stall door an	2	2-10 times	2	2	escape	0	1	0	0	0	0	0	m	1	8,0	NA	NA					1	single	1	restricted	1	restricted			
468	door_gate_Germ	quest_doo	2	2367,0	open door - gate	open door - gate, frees other horses	2	2-10 times	2	3	social	0	0	0	1	1	0	0	m	1	8,0	NA	NA					1	single	1	restricted	1	restricted			
469	door_gate_Germ	quest_doo	2	2368,0	open door - gate	open door - gate, own door , stall door an	3	more than 20 times	4	2	escape	0	1	0	0	0	0	0	m	1	21,0	NA	Donkey	7	6000	4	power	2	group	2	unrestricted pasture	2	unrestricted contact	1	restricted roughage	
470	door_gate_Germ	quest_doo	2	2368,0	open door - gate	open door - gate, feed room door	3	more than 20 times	4	1	feeding	1	0	0	0	0	0	0	m	1	21,0	NA	Donkey	7	6000	4	power	2	group	2	unrestricted pasture	2	unrestricted contact	1	restricted roughage	
471	door_gate_Germ	quest_doo	2	2368,0	open door - gate	open door - gate, frees other horses	3	more than 20 times	4	3	social	0	0	0	1	1	0	0	m	1	21,0	NA	Donkey	7	6000	4	power	2	group	2	unrestricted pasture	2	unrestricted contact	1	restricted roughage	
472	door_gate_Germ	quest_doo	2	2370,0	open door - gate	open door - gate	1	2-10 times	2	1	feeding	1	0	0	0	0	0	0	g	2	5,0	NA	Pony	2	100	5	robustness	2	group	2	unrestricted pasture	2	unrestricted contact	2	unrestrict roughage	
473	door_gate_Germ	quest_doo	2	2371,0	open door - gate	open door - gate	1	NA		5	play	0	0	0	0	0	0	1	g	2	8,0	NA	Warmblood	1	350	3	cooperativeness	2	group	2	unrestricted pasture	2	unrestricted contact	1	restricted roughage	
474	door_gate_Germ	quest_doo	2	2373,0	open door - gate	open door - gate, own door , stall door an	3	2-10 times	2	2	escape	0	1	0	0	0	0	0	g	2	11,0	NA	Draught horse	4	500	4	power	2	group	2	unrestricted pasture	2	unrestricted contact	2	unrestrict roughage	
475	door_gate_Germ	quest_doo	2	2373,0	open door - gate	open door - gate, feed room door	3	2-10 times	2	1	feeding	1	0	0	0	0	0	0	g	2	11,0	NA	Draught horse	4	500	4	power	2	group	2	unrestricted pasture	2	unrestricted contact	2	unrestrict roughage	

1	sorce	video / quest	video_quest_num	sID	name behaviour	behaviour	nr reported behaviours	how often was the behaviour shown	behaviour frequency	behaviour category num	behaviour_category	feeding behaviour	escape behaviour	social behaviour human	social behaviour horse	social behaviour	comfort behaviour	unknown play	sex	sex num	age	breed	breed-type	breed type num	length of domestication	purpose of domestication	purpose of domestication	single group stabling	single group stabling	restricted unrestricted pasture	restricted unrestricted pasture	restricted unrestricted contact with horses	restricted unrestricted contact with horses	restricted unrestric. roughage	restricted unrestricted roughage	links
476	door_gate_Germ	quest_doo	2	2373,0	open door - gate	open door - gate, frees other horses	3	2-10 times	2	3	social	0	0	0	1	1	0	0	g	2	11,0	NA	Draught horse	4	500	4	power	2	group	2	unrestricted pasture	2	unrestricted contact	2	unrestrict roughage	
477	door_gate_Germ	quest_doo	2	2374,0	open door - gate	open door - gate	2	2-10 times	2	2	escape	0	1	0	0	0	0	0	g	2	8,0	NA	Warmblood	1	350	3	cooperativeness	1	single	1	restricted pasture	1	restricted	1	restricted roughage	
478	door_gate_Germ	quest_doo	2	2374,0	unty itself	unty itself	2	2-10 times	2	2	escape	0	1	0	0	0	0	0	g	2	8,0	NA	Warmblood	1	350	3	cooperativeness	1	single	1	restricted pasture	1	restricted	1	restricted roughage	
479	door_gate_Germ	quest_doo	2	2379,0	open door - gate	open door - gate, own door , stall door an	2	2-10 times	2	2	escape	0	1	0	0	0	0	0	m	1	22,0	NA	Pony	2	100	5	robustness	2	group	2	unrestricted pasture	2	unrestricted contact	1	restricted roughage	
480	door_gate_Germ	quest_doo	2	2379,0	open door - gate	open door - gate, feed room door	2	2-10 times	2	1	feeding	1	0	0	0	0	0	0	m	1	22,0	NA	Pony	2	100	5	robustness	2	group	2	unrestricted pasture	2	unrestricted contact	1	restricted roughage	
481	door_gate_Germ	quest_doo	2	2379,0	open door - gate	open door - gate, own door , stall door an	2	2-10 times	2	2	escape	0	1	0	0	0	0	0	NA		1,0	NA	Mule	7	3000	4	power	2	group	2	unrestricted pasture	2	unrestricted contact	1	restricted roughage	
482	door_gate_Germ	quest_doo	2	2379,0	open door - gate	open door - gate, feed room door	2	2-10 times	2	1	feeding	1	0	0	0	0	0	0	NA		1,0	NA	Mule	7	3000	4	power	2	group	2	unrestricted pasture	2	unrestricted contact	1	restricted roughage	
483	door_gate_Germ	quest_doo	2	2381,0	open door - gate	open door - gate, own door , stall door an	3	11-20 times	3	2	escape	0	1	0	0	0	0	0	g	2	27,0	NA	Pony	2	100	5	robustness	2	group	2	unrestricted pasture	2	unrestricted contact	1	restricted roughage	
484	door_gate_Germ	quest_doo	2	2381,0	open door - gate	open door - gate, feed room door	3	11-20 times	3	1	feeding	1	0	0	0	0	0	0	g	2	27,0	NA	Pony	2	100	5	robustness	2	group	2	unrestricted pasture	2	unrestricted contact	1	restricted roughage	
485	door_gate_Germ	quest_doo	2	2381,0	open door - gate	open door - gate, frees other horses	3	11-20 times	3	3	social	0	0	0	1	1	0	0	g	2	27,0	NA	Pony	2	100	5	robustness	2	group	2	unrestricted pasture	2	unrestricted contact	1	restricted roughage	
486	door_gate_Germ	quest_doo	2	2383,0	open door - gate	open door - gate, own door , stall door an	2	2-10 times	2	2	escape	0	1	0	0	0	0	0	g	2	16,0	NA	Pony	2	100	5	robustness	2	group	2	unrestricted pasture	2	unrestricted contact	1	restricted roughage	
487	door_gate_Germ	quest_doo	2	2383,0	open door - gate	open door - gate, feed room door	2	2-10 times	2	1	feeding	1	0	0	0	0	0	0	g	2	16,0	NA	Pony	2	100	5	robustness	2	group	2	unrestricted pasture	2	unrestricted contact	1	restricted roughage	
488	door_gate_Germ	quest_doo	2	2385,0	open door - gate	open door - gate	1	more than 20 times	4	5	play	0	0	0	0	0	0	1	g	2	2,0	NA	Warmblood	1	350	3	cooperativeness	1	single	2	unrestricted pasture	1	restricted	1	restricted roughage	
489	door_gate_Germ	quest_doo	2	2389,0	open door - gate	open door - gate	1	more than 20 times	4	2	escape	0	1	0	0	0	0	0	g	2	7,0	NA	Warmblood	1	350	3	cooperativeness	2	group	2	unrestricted pasture	2	unrestricted contact	2	unrestrict roughage	
490	door_gate_Germ	quest_doo	2	2390,0	open door - gate	open door - gate, own door , stall door an	5	11-20 times	3	2	escape	0	1	0	0	0	0	0	g	2	9,0	NA	Pony	2	100	5	robustness	1	single	1	restricted pasture	1	restricted	2	unrestrict roughage	
491	door_gate_Germ	quest_doo	2	2390,0	open door - gate	open door - gate, feed room door	5	11-20 times	3	1	feeding	1	0	0	0	0	0	0	g	2	9,0	NA	Pony	2	100	5	robustness	1	single	1	restricted pasture	1	restricted	2	unrestrict roughage	
492	door_gate_Germ	quest_doo	2	2390,0	open door - gate	open door - gate, openes and closes doo	5	11-20 times	3	5	play	0	0	0	0	0	0	1	g	2	9,0	NA	Pony	2	100	5	robustness	1	single	1	restricted pasture	1	restricted	2	unrestrict roughage	
493	door_gate_Germ	quest_doo	2	2390,0	unty itself	unty itself, open carabiner	5	11-20 times	3	2	escape	0	1	0	0	0	0	0	g	2	9,0	NA	Pony	2	100	5	robustness	1	single	1	restricted pasture	1	restricted	2	unrestrict roughage	
494	door_gate_Germ	quest_doo	2	2390,0	unty itself	unty itself, open knots	5	11-20 times	3	2	escape	0	1	0	0	0	0	0	g	2	9,0	NA	Pony	2	100	5	robustness	1	single	1	restricted pasture	1	restricted	2	unrestrict roughage	
495	door_gate_Germ	quest_doo	2	2391,0	open door - gate	open door - gate, own door , stall door an	2	dayly	5	2	escape	0	1	0	0	0	0	0	g	2	13,0	NA	Draught horse	4	500	4	power	2	group	2	unrestricted pasture	2	unrestricted contact	2	unrestrict roughage	
496	door_gate_Germ	quest_doo	2	2391,0	open door - gate	open door - gate, feed room door	2	dayly	5	1	feeding	1	0	0	0	0	0	0	g	2	13,0	NA	Draught horse	4	500	4	power	2	group	2	unrestricted pasture	2	unrestricted contact	2	unrestrict roughage	
497	door_gate_Germ	quest_doo	2	2393,0	open door - gate	open door - gate	1	more than 20 times	4	2	escape	0	1	0	0	0	0	0	g	2	3,0	NA	Warmblood	1	350	3	cooperativeness	1	single	1	restricted pasture	1	restricted	1	restricted roughage	
498	door_gate_Germ	quest_doo	2	2396,0	open door - gate	open door - gate	1	2-10 times	2	2	escape	0	1	0	0	0	0	0	g	2	8,0	NA	Warmblood	1	350	3	cooperativeness	1	single	1	restricted pasture	1	restricted	1	restricted roughage	
499	door_gate_Germ	quest_doo	2	2397,0	open door - gate	open door - gate, own door , stall door an	3	more than 20 times	4	2	escape	0	1	0	0	0	0	0	m	1	5,0	NA	Pony	2	100	5	robustness	2	group	2	unrestricted pasture	2	unrestricted contact	1	restricted roughage	
500	door_gate_Germ	quest_doo	2	2397,0	open door - gate	open door - gate, feed room door	3	more than 20 times	4	1	feeding	1	0	0	0	0	0	0	m	1	5,0	NA	Pony	2	100	5	robustness	2	group	2	unrestricted pasture	2	unrestricted contact	1	restricted roughage	
501	door_gate_Germ	quest_doo	2	2397,0	open door - gate	open door - gate, goes to other horse	3	more than 20 times	4	3	social	0	0	0	1	1	0	0	m	1	5,0	NA	Pony	2	100	5	robustness	2	group	2	unrestricted pasture	2	unrestricted contact	1	restricted roughage	
502	door_gate_Germ	quest_doo	2	2408,0	open door - gate	open door - gate	1	2-10 times	2	5	play	0	0	0	0	0	0	1	m	1	7,0	NA	Arabian horse	3	4000	1	endurance	1	single	1	restricted pasture	1	restricted	1	restricted roughage	
503	door_gate_Germ	quest_doo	2	2409,0	open door - gate	open door - gate	1	2-10 times	2	5	play	0	0	0	0	0	0	1	g	2	7,0	NA	Warmblood	1	350	3	cooperativeness	1	single	2	unrestricted pasture	2	unrestricted contact	2	unrestrict roughage	
504	door_gate_Germ	quest_doo	2	2410,0	open door - gate	open door - gate	1	11-20 times	3	2	escape	0	1	0	0	0	0	0	m	1	10,0	NA	Warmblood	1	350	3	cooperativeness	1	single	1	restricted pasture	1	restricted	1	restricted roughage	
505	door_gate_Germ	quest_doo	2	2411,0	open door - gate	open door - gate	1	2-10 times	2	2	escape	0	1	0	0	0	0	0	g	2	5,0	NA	Arabian horse	3	4000	1	endurance	2	group	2	unrestricted pasture	2	unrestricted contact	2	unrestrict roughage	
506	door_gate_Germ	quest_doo	2	2418,0	open door - gate	open door - gate	1	2-10 times	2	5	play	0	0	0	0	0	0	1	g	2	12,0	NA	Warmblood	1	350	3	cooperativeness	2	group	2	unrestricted pasture	2	unrestricted contact	2	unrestrict roughage	
507	door_gate_Germ	quest_doo	2	2419,0	open door - gate	open door - gate	1	2-10 times	2	2	escape	0	1	0	0	0	0	0	m	1	8,0	NA	Warmblood	1	350	3	cooperativeness	1	single	1	restricted pasture	1	restricted	1	restricted roughage	
508	door_gate_Germ	quest_doo	2	2420,0	open door - gate	open door - gate	5	NA		2	escape	0	1	0	0	0	0	0	g	2	11,0	NA	Warmblood	1	350	3	cooperativeness	2	group	1	restricted pasture	2	unrestricted contact	2	unrestrict roughage	
509	door_gate_Germ	quest_doo	2	2420,0	manipulate things	open boots	5	NA		4	comfort	0	0	0	0	0	1	0	g	2	11,0	NA	Warmblood	1	350	3	cooperativeness	2	group	1	restricted pasture	2	unrestricted contact	2	unrestrict roughage	
510	door_gate_Germ	quest_doo	2	2420,0	manipulate things	unty itself	5	NA		2	escape	0	1	0	0	0	0	0	g	2	11,0	NA	Warmblood	1	350	3	cooperativeness	2	group	1	restricted pasture	2	unrestricted contact	2	unrestrict roughage	

1	sorce	video / quest	video_quest_num	sID	name behaviour	behaviour	nr reported behaviours	how often was the behaviour shown	behaviour frequency	behaviour category num	behaviour _category	feeding behaviour	escape behaviour	social behaviour human	social behaviour horse	social behaviour	comfort behaviour	unknown play	sex	sex num	age	breed	breed-type	breed type num	length of domestication	purpose of domestication	purpose of domestication	single group stabling	single group stabling	restricted unrestricted pasture	restricted unrestricted pasture	restricted unrestricted contact with horses	restricted unrestricted contact with horses	restricted unrestric. roughage	restricted unrestricted roughage	links
511	door_gate_Germ	quest_doo	2	2420,0	manipulate things	take off rugs	5	NA		4	comfort	0	0	0	0	0	1	0	g	2	11,0	NA	Warmblood	1	350	3	cooperativeness	2	group	1	restricted pasture	2	unrestricted contact	2	unrestrict roughage	
512	door_gate_Germ	quest_doo	2	2420,0	manipulate things	apport things	5	NA		5	play	0	0	0	0	0	0	1	g	2	11,0	NA	Warmblood	1	350	3	cooperativeness	2	group	1	restricted pasture	2	unrestricted contact	2	unrestrict roughage	
513	door_gate_Germ	quest_doo	2	2421,0	open door - gate	open door - gate, own door , stall door an	3	more than 20 times	4	2	escape	0	1	0	0	0	0	0	g	2	22,0	NA	Warmblood	1	350	3	cooperativeness	2	group	2	unrestricted pasture	2	unrestricted contact	1	restricted roughage	
514	door_gate_Germ	quest_doo	2	2421,0	open door - gate	open door - gate, feed room door	3	more than 20 times	4	1	feeding	1	0	0	0	0	0	0	g	2	22,0	NA	Warmblood	1	350	3	cooperativeness	2	group	2	unrestricted pasture	2	unrestricted contact	1	restricted roughage	
515	door_gate_Germ	quest_doo	2	2421,0	open door - gate	open door - gate, frees other horses	3	more than 20 times	4	3	social	0	0	0	1	1	0	0	g	2	22,0	NA	Warmblood	1	350	3	cooperativeness	2	group	2	unrestricted pasture	2	unrestricted contact	1	restricted roughage	
516	door_gate_Germ	quest_doo	2	2422,0	open door - gate	open door - gate	1	2-10 times	2	2	escape	0	1	0	0	0	0	0	g	2	7,0	NA	Pony	2	100	5	robustness	2	group	2	unrestricted pasture	2	unrestricted contact	2	unrestrict roughage	
517	door_gate_Germ	quest_doo	2	2425,0	open door - gate	open door - gate	2	11-20 times	3	5	play	0	0	0	0	0	0	1	g	2	3,0	NA	Warmblood	1	350	3	cooperativeness	1	single	1	restricted pasture	1	restricted	1	restricted roughage	
518	door_gate_Germ	quest_doo	2	2425,0	manipulate things	shut water tap	2	11-20 times	3	1	feeding	1	0	0	0	0	0	0	g	2	3,0	NA	Warmblood	1	350	3	cooperativeness	1	single	1	restricted pasture	1	restricted	1	restricted roughage	
519	door_gate_Germ	quest_doo	2	2427,0	open door - gate	open door - gate	1	more than 20 times	4	2	escape	0	1	0	0	0	0	0	s	3	5,0	NA	Warmblood	1	350	3	cooperativeness	2	group	1	restricted pasture	2	unrestricted contact	2	unrestrict roughage	
520	door_gate_Germ	quest_doo	2	2432,0	open door - gate	open door - gate	1	2-10 times	2	2	escape	0	1	0	0	0	0	0	g	2	13,0	NA	Pony	2	100	5	robustness	2	group	2	unrestricted pasture	2	unrestricted contact	1	restricted roughage	
521	door_gate_Germ	quest_doo	2	2434,0	open door - gate	open door - gate, own door , stall door an	2	dayly	5	2	escape	0	1	0	0	0	0	0	g	2	13,0	NA	Warmblood	1	350	3	cooperativeness	2	group	2	unrestricted pasture	2	unrestricted contact	1	restricted roughage	
522	door_gate_Germ	quest_doo	2	2434,0	open door - gate	open door - gate, feed room door	2	dayly	5	1	feeding	1	0	0	0	0	0	0	g	2	13,0	NA	Warmblood	1	350	3	cooperativeness	2	group	2	unrestricted pasture	2	unrestricted contact	1	restricted roughage	
523	door_gate_Germ	quest_doo	2	2435,0	open door - gate	open door - gate, frees other horses	2	2-10 times	2	3	social	0	0	0	1	1	0	0	g	2	2,0	NA	Warmblood	1	350	3	cooperativeness	1	single	1	restricted pasture	1	restricted	2	unrestrict roughage	
524	door_gate_Germ	quest_doo	2	2435,0	open door - gate	open door - gate, opens doors from the ou	2	2-10 times	2	5	play	0	0	0	0	0	0	1	g	2	2,0	NA	Warmblood	1	350	3	cooperativeness	1	single	1	restricted pasture	1	restricted	2	unrestrict roughage	
525	door_gate_Germ	quest_doo	2	2436,0	open door - gate	open door - gate, own door , stall door an	2	more than 20 times	4	2	escape	0	1	0	0	0	0	0	m	1	11,0	NA	Warmblood	1	350	3	cooperativeness	2	group	1	restricted pasture	2	unrestricted contact	2	unrestrict roughage	
526	door_gate_Germ	quest_doo	2	2436,0	open door - gate	open door - gate, frees other horses	2	more than 20 times	4	3	social	0	0	0	1	1	0	0	m	1	11,0	NA	Warmblood	1	350	3	cooperativeness	2	group	1	restricted pasture	2	unrestricted contact	2	unrestrict roughage	
527	door_gate_Germ	quest_doo	2	2437,0	open door - gate	open door - gate, own door , stall door an	3	dayly	5	2	escape	0	1	0	0	0	0	0	m	1	5,0	NA	Warmblood	1	350	3	cooperativeness	1	single	1	restricted pasture	1	restricted	1	restricted roughage	
528	door_gate_Germ	quest_doo	2	2437,0	open door - gate	open door - gate, feed room door	3	dayly	5	1	feeding	1	0	0	0	0	0	0	m	1	5,0	NA	Warmblood	1	350	3	cooperativeness	1	single	1	restricted pasture	1	restricted	1	restricted roughage	
529	door_gate_Germ	quest_doo	2	2437,0	open door - gate	open door - gate, frees other horses	3	dayly	5	3	social	0	0	0	1	1	0	0	m	1	5,0	NA	Warmblood	1	350	3	cooperativeness	1	single	1	restricted pasture	1	restricted	1	restricted roughage	
530	door_gate_Germ	quest_doo	2	2443,0	open door - gate	open door - gate	1	2-10 times	2	2	escape	0	1	0	0	0	0	0	s	3	20,0	NA	Warmblood	1	350	3	cooperativeness	1	single	1	restricted pasture	1	restricted	1	restricted roughage	
531	door_gate_Germ	quest_doo	2	2446,0	open door - gate	open door - gate	1	more than 20 times	4	5	play	0	0	0	0	0	0	1	m	1	26,0	NA	Warmblood	1	350	3	cooperativeness	1	single	1	restricted pasture	1	restricted	1	restricted roughage	
532	door_gate_Germ	quest_doo	2	2448,0	open door - gate	open door - gate	1	2-10 times	2	5	play	0	0	0	0	0	0	1	g	2	5,0	NA	Pony	2	100	5	robustnes	2	group	1	restricted	2	unrestricted	1	restricted	
533	door_gate_Germ	quest_doo	2	2449,0	open door - gate	open door - gate, own door , stall door an	2	2-10 times	2	2	escape	0	1	0	0	0	0	0	g	2	13,0	NA	Pony	2	100	5	robustnes	2	group	1	restricted	2	unrestricted	1	restricted	
534	door_gate_Germ	quest_doo	2	2449,0	open door - gate	open door - gate, frees other horses	2	2-10 times	2	3	social	0	0	0	1	1	0	0	g	2	13,0	NA	Pony	2	100	5	robustnes	2	group	1	restricted	2	unrestricted	1	restricted	
535	door_gate_Germ	quest_doo	2	2451,0	open door - gate	open door - gate, own door , stall door an	3	more than 20 times	4	2	escape	0	1	0	0	0	0	0	m	1	5,0	NA	Warmblood	1	350	3	cooperativeness	2	group	2	unrestricted pasture	2	unrestricted contact	1	restricted roughage	
536	door_gate_Germ	quest_doo	2	2451,0	open door - gate	open door - gate, feed room door	3	more than 20 times	4	1	feeding	1	0	0	0	0	0	0	m	1	5,0	NA	Warmblood	1	350	3	cooperativeness	2	group	2	unrestricted pasture	2	unrestricted contact	1	restricted roughage	
537	door_gate_Germ	quest_doo	2	2451,0	open door - gate	open door - gate, frees other horses	3	more than 20 times	4	3	social	0	0	0	1	1	0	0	m	1	5,0	NA	Warmblood	1	350	3	cooperativeness	2	group	2	unrestricted pasture	2	unrestricted contact	1	restricted roughage	
538	door_gate_Germ	quest_doo	2	2454,0	open door - gate	open door - gate	1	more than 20 times	4	2	escape	0	1	0	0	0	0	0	g	2	3,0	NA	Arabian horse	3	4000	1	endurance	2	group	2	unrestricted pasture	2	unrestricted contact	2	unrestrict roughage	
539	door_gate_Germ	quest_doo	2	2509,0	open door - gate	open door - gate	1	11-20 times	3	2	escape	0	1	0	0	0	0	0	m	1	0,5	NA	Arabian horse	3	4000	1	endurance	2	group	2	unrestricted pasture	2	unrestricted contact	1	restricted roughage	
540	door_gate_Germ	video	3	2510,0	open door - gate	open door - gate	1	more than 20 times	4	1	feeding	1	0	0	0	0	0	0	NA			NA	Donkey	7	6000	4	power	2	group	2	unrestricted pasture	2	unrestricted contact	1	restricted roughage	https://www.youtube.com/watch?v=sa5cgpMO0xA
541	door_gate_Engl.	video	3	2206,0	open door - gate	open door - gate	1	NA		2	escape	0	1	0	0	0	0	0	g	2		NA	Warmblood	1	350	3	cooperativeness	1	single							https://www.youtube.com/watch?v=ez6nOMYfclc
542	door_gate_Engl.	video	3	2207,0	open door - gate	open door - gate	1	NA		2	escape	0	1	0	0	0	0	0	g	2		NA	Warmblood	1	350	3	cooperativeness	1	single							https://www.youtube.com/watch?v=owjvgT8H4g4
543	door_gate_Engl.	video	3	2208,0	open door - gate	open door - gate	1	NA		2	escape	0	1	0	0	0	0	0	NA			NA	Warmblood	1	350	3	cooperativeness	1	single							https://www.youtube.com/watch?v=d_xe1lNWkq0
544	door_gate_Engl.	video	3	2209,0	open door - gate	open door - gate	1	NA		2	escape	0	1	0	0	0	0	0	m	1		NA	Arabian horse	3	4000	1	endurance	1	single							https://www.youtube.com/watch?v=5XUu068bbh0
545	door_gate_Engl.	quest_doo	2	2245,0	open door - gate	open door - gate, own door , stall door an	2	more than 20 times	4	2	escape	0	1	0	0	0	0	0	g	2	8,0	NA	Pony	2	100	5	robustness	2	group	2	unrestricted pasture	2	unrestricted contact	2	unrestrict roughage	
																											robustnes				unrestricted		unrestricted		unrestrict	

1	sorce	video / quest	video_quest_num	sID	name behaviour	behaviour	nr reported behaviours	how often was the behaviour shown	behaviour frequency	behaviour category num	behaviour_category	feeding behaviour	escape behaviour	social behaviour human	social behaviour horse	social behaviour	comfort behaviour	unknown play	sex	sex num	age	breed	breed-type	breed type num	length of domestication	purpose of domestication	purpose of domestication	single group stabling	single group stabling	restricted unrestricted pasture	restricted unrestricted pasture	restricted unrestricted contact with horses	restricted unrestricted contact with horses	restricted unrestric. roughage	restricted unrestricted roughage	links
546	door_gate_Engl.	quest_doo	2	2245,0	open door - gate	open door - gate, feed room door	2	more than 20 times	4	1	feeding	1	0	0	0	0	0	0	g	2	8,0	NA	Pony	2	100	5	robustness	2	group	2	unrestricted pasture	2	unrestricted contact	2	unrestrict roughage	
547	door_gate_Engl.	quest_doo	2	2259,0	open door - gate	open door - gate	2	11-20 times	3	2	escape	0	1	0	0	0	0	0	g	2	5,0	NA	Warmblood	1	350	3	cooperativeness	1	single	1	restricted pasture	1	restricted	2	unrestrict roughage	
548	door_gate_Engl.	quest_doo	2	2259,0	manipulate things	take of fly protection (head)	2	11-20 times	3	4	comfort	0	0	0	0	0	1	0	g	2	5,0	NA	Warmblood	1	350	3	cooperativeness	1	single	1	restricted pasture	1	restricted	2	unrestrict roughage	
549	door_gate_Engl.	quest_doo	2	2263,0	open door - gate	crawls through fence, to escape	2	dayly	5	2	escape	0	1	0	0	0	0	0	m	1	7,0	NA	Warmblood	1	350	3	cooperativeness	2	group	2	unrestricted pasture	2	unrestricted contact	2	unrestrict roughage	
550	door_gate_Engl.	quest_doo	2	2263,0	open door - gate	crawls through fence, to reach feed	2	dayly	5	1	feeding	1	0	0	0	0	0	0	m	1	7,0	NA	Warmblood	1	350	3	cooperativeness	2	group	2	unrestricted pasture	2	unrestricted contact	2	unrestrict roughage	
551	door_gate_Engl.	quest_doo	2	2266,0	open door - gate	open door - gate, own door , stall door an	2	11-20 times	3	2	escape	0	1	0	0	0	0	0	g	2	7,0	NA	Warmblood	1	350	3	cooperativeness	1	single	1	restricted pasture	1	restricted	2	unrestrict roughage	
552	door_gate_Engl.	quest_doo	2	2266,0	open door - gate	open door - gate, feed room door	2	11-20 times	3	1	feeding	1	0	0	0	0	0	0	g	2	7,0	NA	Warmblood	1	350	3	cooperativeness	1	single	1	restricted pasture	1	restricted	2	unrestrict roughage	
553	door_gate_Engl.	quest_doo	2	2267,0	open door - gate	open door - gate, opens doors and closes	2	11-20 times	3	5	play	0	0	0	0	0	0	1	m	1	20,0	NA	Warmblood	1	350	3	cooperativeness	2	group	2	unrestricted pasture	2	unrestricted contact	1	restricted roughage	
554	door_gate_Engl.	quest_doo	2	2267,0	open door - gate	open door - gate, feed room door	2	11-20 times	3	1	feeding	1	0	0	0	0	0	0	m	1	20,0	NA	Warmblood	1	350	3	cooperativeness	2	group	2	unrestricted pasture	2	unrestricted contact	1	restricted roughage	
555	door_gate_Engl.	quest_doo	2	2269,0	open door - gate	open door - gate, own door , stall door an	3	dayly	5	2	escape	0	1	0	0	0	0	0	m	1	5,0	NA	Warmblood	1	350	3	cooperativeness	1	single	1	restricted pasture	1	restricted	2	unrestrict roughage	
556	door_gate_Engl.	quest_doo	2	2269,0	open door - gate	open door - gate, frees other horses	3	dayly	5	3	social	0	0	0	1	1	0	0	m	1	5,0	NA	Warmblood	1	350	3	cooperativeness	1	single	1	restricted pasture	1	restricted	2	unrestrict roughage	
557	door_gate_Engl.	quest_doo	2	2269,0	open door - gate	manipulate things, selcts item son cupbord	3	dayly	5	5	play	0	0	0	0	0	0	1	m	1	5,0	NA	Warmblood	1	350	3	cooperativeness	1	single	1	restricted pasture	1	restricted	2	unrestrict roughage	
558	door_gate_Engl.	quest_doo	2	2271,0	open door - gate	open door - gate, own door , stall door an	6	dayly	5	2	escape	0	1	0	0	0	0	0	g	2	10,0	NA	Warmblood	1	350	3	cooperativeness	1	single	1	restricted pasture	1	restricted	1	restricted roughage	
559	door_gate_Engl.	quest_doo	2	2271,0	open door - gate	open door - gate, feed room door	6	dayly	5	1	feeding	1	0	0	0	0	0	0	g	2	10,0	NA	Warmblood	1	350	3	cooperativeness	1	single	1	restricted pasture	1	restricted	1	restricted roughage	
560	door_gate_Engl.	quest_doo	2	2271,0	open door - gate	open door - gate, fress other horses	6	dayly	5	3	social	0	0	0	1	1	0	0	g	2	10,0	NA	Warmblood	1	350	3	cooperativeness	1	single	1	restricted pasture	1	restricted	1	restricted roughage	
561	door_gate_Engl.	quest_doo	2	2271,0	open door - gate	open door - gate, open and closedes door	6	dayly	5	5	play	0	0	0	0	0	0	1	g	2	10,0	NA	Warmblood	1	350	3	cooperativeness	1	single	1	restricted pasture	1	restricted	1	restricted roughage	
562	door_gate_Engl.	quest_doo	2	2271,0	manipulate things	unty itself	6	dayly	5	2	escape	0	1	0	0	0	0	0	g	2	10,0	NA	Warmblood	1	350	3	cooperativeness	1	single	1	restricted pasture	1	restricted	1	restricted roughage	
563	door_gate_Engl.	quest_doo	2	2271,0	manipulate things	take off halter	6	dayly	5	2	escape	0	1	0	0	0	0	0	g	2	10,0	NA	Warmblood	1	350	3	cooperativeness	1	single	1	restricted pasture	1	restricted	1	restricted roughage	
564	door_gate_Engl.	quest_doo	2	2273,0	open door - gate	open door - gate, own door , stall door an	2	more than 20 times	4	2	escape	0	1	0	0	0	0	0	g	2	9,0	NA	Warmblood	1	350	3	cooperativeness	1	single	2	unrestricted pasture	1	restricted	2	unrestrict roughage	
565	door_gate_Engl.	quest_doo	2	2273,0	open door - gate	open door - gate, frees other horses	2	more than 20 times	4	3	social	0	0	0	1	1	0	0	g	2	9,0	NA	Warmblood	1	350	3	cooperativeness	1	single	2	unrestricted pasture	1	restricted	2	unrestrict roughage	
566	door_gate_Engl.	quest_doo	2	2274,0	open door - gate	open door - gate, own door , stall door an	2	NA		2	escape	0	1	0	0	0	0	0	m	1	7,0	NA	Arabian horse	3	4000	1	endurance	1	single	1	restricted pasture	1	restricted	1	restricted roughage	
567	door_gate_Engl.	quest_doo	2	2274,0	open door - gate	open door - gate, feed room door	2	NA		1	feeding	1	0	0	0	0	0	0	m	1	7,0	NA	Arabian horse	3	4000	1	endurance	1	single	1	restricted pasture	1	restricted	1	restricted roughage	
568	door_gate_Engl.	quest_doo	2	2277,0	open door - gate	open door - gate	1	2-10 times	2	2	escape	0	1	0	0	0	0	0	m	1	4,0	NA	Warmblood	1	350	3	cooperativeness	2	group	2	unrestricted pasture	2	unrestricted contact	1	restricted roughage	
569	door_gate_Engl.	quest_doo	2	2278,0	open door - gate	open door - gate, own door , stall door an	3	more than 20 times	4	2	escape	0	1	0	0	0	0	0	m	1	4,0	NA	Warmblood	1	350	3	cooperativeness	2	group	2	unrestricted pasture	2	unrestricted contact	2	unrestrict roughage	
570	door_gate_Engl.	quest_doo	2	2278,0	open door - gate	open door - gate, frees other horses	3	more than 20 times	4	3	social	0	0	0	1	1	0	0	m	1	4,0	NA	Warmblood	1	350	3	cooperativeness	2	group	2	unrestricted pasture	2	unrestricted contact	2	unrestrict roughage	
571	door_gate_Engl.	quest_doo	2	2278,0	open door - gate	open door - gate, opens doors but does n	3	more than 20 times	4	5	play	0	0	0	0	0	0	1	m	1	4,0	NA	Warmblood	1	350	3	cooperativeness	2	group	2	unrestricted pasture	2	unrestricted contact	2	unrestrict roughage	
572	door_gate_Engl.	quest_doo	2	2317,0	open door - gate	open door - gate, own door , stall door an	4	11-20 times	3	2	escape	0	1	0	0	0	0	0	g	2	7,0	NA	Draught horse	4	500	4	power	2	group	2	unrestricted pasture	2	unrestricted contact	1	restricted roughage	
573	door_gate_Engl.	quest_doo	2	2317,0	open door - gate	open door - gate, feed room door	4	11-20 times	3	1	feeding	1	0	0	0	0	0	0	g	2	7,0	NA	Draught horse	4	500	4	power	2	group	2	unrestricted pasture	2	unrestricted contact	1	restricted roughage	
574	door_gate_Engl.	quest_doo	2	2317,0	open door - gate	open door - gate, frees other horses	4	11-20 times	3	3	social	0	0	0	1	1	0	0	g	2	7,0	NA	Draught horse	4	500	4	power	2	group	2	unrestricted pasture	2	unrestricted contact	1	restricted roughage	
575	door_gate_Engl.	quest_doo	2	2317,0	open door - gate	open door - gate, opens doors but stays	4	11-20 times	3	5	play	0	0	0	0	0	0	1	g	2	7,0	NA	Draught horse	4	500	4	power	2	group	2	unrestricted pasture	2	unrestricted contact	1	restricted roughage	
576	door_gate_Engl.	quest_doo	2	2321,0	open door - gate	open door - gate	1	more than 20 times	4	2	escape	0	1	0	0	0	0	0	NA			NA	NA					1	single	2	unrestricted pasture	1	restricted	2	unrestrict roughage	
577	door_gate_Engl.	quest_doo	2	2330,0	open door - gate	open door - gate, own door , stall door an	4	11-20 times	3	2	escape	0	1	0	0	0	0	0	g	2	5,0	NA	Warmblood	1	350	3	cooperativeness	1	single	1	restricted pasture	1	restricted	2	unrestrict roughage	
578	door_gate_Engl.	quest_doo	2	2330,0	open door - gate	open door - gate, feed room door	4	11-20 times	3	1	feeding	1	0	0	0	0	0	0	g	2	5,0	NA	Warmblood	1	350	3	cooperativeness	1	single	1	restricted pasture	1	restricted	2	unrestrict roughage	
579	door_gate_Engl.	quest_doo	2	2330,0	open door - gate	open door - gate, frees other horses	4	11-20 times	3	3	social	0	0	0	1	1	0	0	g	2	5,0	NA	Warmblood	1	350	3	cooperativeness	1	single	1	restricted pasture	1	restricted	2	unrestrict roughage	
580	door_gate_Engl.	quest_doo	2	2330,0	open door - gate	manipulate things, remove paddock pannel	4	11-20 times	3	5	play	0	0	0	0	0	0	1	g	2	5,0	NA	Warmblood	1	350	3	cooperativeness	1	single	1	restricted pasture	1	restricted	2	unrestrict roughage	

1	sorce	video / quest	video_quest_num	sID	name behaviour	behaviour	nr reported behaviours	how often was the behaviour shown	behaviour frequency	behaviour category num	behaviour_category	feeding behaviour	escape behaviour	social behaviour human	social behaviour horse	social behaviour	comfort behaviour	unknown play	sex	sex num	age	breed	breed-type	breed type num	length of domestication	purpose of domestication	purpose of domestication	single group stabling	single group stabling	restricted unrestricted pasture	restricted unrestricted pasture	restricted unrestricted contact with horses	restricted unrestricted contact with horses	restricted unrestric. roughage	restricted unrestricted roughage	links
581	door_gate_Engl.	quest_doo	2	2330,1	open door - gate	open door - gate, feed room door	2	11-20 times	3	1	feeding	1	0	0	0	0	0	0	NA			NA	Pony	2	100	5	robustness	1	single	1	restricted pasture	1	restricted	2	unrestrict roughage	
582	door_gate_Engl.	quest_doo	2	2330,1	open door - gate	open door - gate, open and closedes door	2	11-20 times	3	5	play	0	0	0	0	0	0	1	NA			NA	Pony	2	100	5	robustness	1	single	1	restricted pasture	1	restricted	2	unrestrict roughage	
583	door_gate_Engl.	quest_doo	2	2339,0	open door - gate	open door - gate	1	11-20 times	3	2	escape	0	1	0	0	0	0	0	m	1	25,0	NA	Pony	2	100	5	robustness	1	single	1	restricted pasture	1	restricted	1	restricted roughage	
584	door_gate_Engl.	quest_doo	2	2347,0	open door - gate	open door - gate, own door , stall door an	2	dayly	5	2	escape	0	1	0	0	0	0	0	g	2	10,0	NA	Warmblood	1	350	3	cooperativeness	1	single	2	unrestricted pasture	1	restricted	2	unrestrict roughage	
585	door_gate_Engl.	quest_doo	2	2347,0	open door - gate	open door - gate, frees other horses	2	dayly	5	3	social	0	0	0	1	1	0	0	g	2	10,0	NA	Warmblood	1	350	3	cooperativeness	1	single	2	unrestricted pasture	1	restricted	2	unrestrict roughage	
586	door_gate_Engl.	quest_doo	2	2353,0	open door - gate	open door - gate, own door , stall door an	3	more than 20 times	4	2	escape	0	1	0	0	0	0	0	g	2	28,0	NA	Warmblood	1	350	3	cooperativeness	1	single	1	restricted pasture	1	restricted	2	unrestrict roughage	
587	door_gate_Engl.	quest_doo	2	2353,0	open door - gate	open door - gate, feed room door	3	more than 20 times	4	1	feeding	1	0	0	0	0	0	0	g	2	28,0	NA	Warmblood	1	350	3	cooperativeness	1	single	1	restricted pasture	1	restricted	2	unrestrict roughage	
588	door_gate_Engl.	quest_doo	2	2353,0	open door - gate	open door - gate, frees other horses	3	more than 20 times	4	3	social	0	0	0	1	1	0	0	g	2	28,0	NA	Warmblood	1	350	3	cooperativeness	1	single	1	restricted pasture	1	restricted	2	unrestrict roughage	
589	door_gate_Engl.	quest_doo	2	2355,0	open door - gate	open door - gate	1	2-10 times	2	2	escape	0	1	0	0	0	0	0	m	1	7,0	NA	Thoroughbred	5	250	2	speed	1	single	1	restricted pasture	1	restricted	2	unrestrict roughage	
590	door_gate_Engl.	quest_doo	2	2360,0	open door - gate	open door - gate, own door , stall door an	2	NA		2	escape	0	1	0	0	0	0	0	g	2	13,0	NA	Warmblood	1	350	3	cooperativeness	1	single	1	restricted pasture	1	restricted	1	restricted roughage	
591	door_gate_Engl.	quest_doo	2	2360,0	open door - gate	open door - gate, feed room-barn	2	NA		1	feeding	1	0	0	0	0	0	0	g	2	13,0	NA	Warmblood	1	350	3	cooperativeness	1	single	1	restricted pasture	1	restricted	1	restricted roughage	
592	door_gate_Engl.	quest_doo	2	2362,0	open door - gate	open door - gate	1	2-10 times	2	2	escape	0	1	0	0	0	0	0	g	2	10,0	NA	Warmblood	1	350	3	cooperativeness	1	single	1	restricted pasture	1	restricted	2	unrestrict roughage	
593	door_gate_Engl.	quest_doo	2	2369,0	open door - gate	open door - gate, own door , stall door an	3	dayly	5	2	escape	0	1	0	0	0	0	0	g	2	6,0	NA	Warmblood	1	350	3	cooperativeness	2	group	1	restricted pasture	2	unrestricted contact	1	restricted roughage	
594	door_gate_Engl.	quest_doo	2	2369,0	open door - gate	open door - gate, feeding area	3	dayly	5	1	feeding	1	0	0	0	0	0	0	g	2	6,0	NA	Warmblood	1	350	3	cooperativeness	2	group	1	restricted pasture	2	unrestricted contact	1	restricted roughage	
595	door_gate_Engl.	quest_doo	2	2369,0	open door - gate	open door - gate, frees other horses	3	dayly	5	3	social	0	0	0	1	1	0	0	g	2	6,0	NA	Warmblood	1	350	3	cooperativeness	2	group	1	restricted pasture	2	unrestricted contact	1	restricted roughage	
596	door_gate_Engl.	quest_doo	2	2380,0	open door - gate	open door - gate, own door , stall door an	4	more than 20 times	4	2	escape	0	1	0	0	0	0	0	m	1	18,0	NA	Thoroughbred	5	250	2	speed	1	single	1	restricted pasture	1	restricted	2	unrestrict roughage	
597	door_gate_Engl.	quest_doo	2	2380,0	open door - gate	open door - gate, feed room	4	more than 20 times	4	1	feeding	1	0	0	0	0	0	0	m	1	18,0	NA	Thoroughbred	5	250	2	speed	1	single	1	restricted pasture	1	restricted	2	unrestrict roughage	
598	door_gate_Engl.	quest_doo	2	2380,0	open door - gate	open door - gate, frees other horses	4	more than 20 times	4	3	social	0	0	0	1	1	0	0	m	1	18,0	NA	Thoroughbred	5	250	2	speed	1	single	1	restricted pasture	1	restricted	2	unrestrict roughage	
599	door_gate_Engl.	quest_doo	2	2380,0	open door - gate	open door - gate, opens doors but does n	4	more than 20 times	4	5	play	0	0	0	0	0	0	1	m	1	18,0	NA	Thoroughbred	5	250	2	speed	1	single	1	restricted pasture	1	restricted	2	unrestrict roughage	
600	door_gate_Engl.	quest_doo	2	2395,0	open door - gate	open door - gate, own door , stall door an	3	more than 20 times	4	2	escape	0	1	0	0	0	0	0	m	1	16,0	NA	Warmblood	1	350	3	cooperativeness	2	group	2	unrestricted pasture	2	unrestricted contact	2	unrestrict roughage	
601	door_gate_Engl.	quest_doo	2	2395,0	open door - gate	open door - gate, feed room door	3	more than 20 times	4	1	feeding	1	0	0	0	0	0	0	m	1	16,0	NA	Warmblood	1	350	3	cooperativeness	2	group	2	unrestricted pasture	2	unrestricted contact	2	unrestrict roughage	
602	door_gate_Engl.	quest_doo	2	2395,0	open door - gate	open door - gate, frees other horses	3	more than 20 times	4	3	social	0	0	0	1	1	0	0	m	1	16,0	NA	Warmblood	1	350	3	cooperativeness	2	group	2	unrestricted pasture	2	unrestricted contact	2	unrestrict roughage	
603	door_gate_Engl.	quest_doo	2	2398,0	open door - gate	open door - gate	1	more than 20 times	4	2	escape	0	1	0	0	0	0	0	m	1	3,0	NA	Pony	2	100	5	robustness	1	single	1	restricted pasture	1	restricted	1	restricted roughage	
604	door_gate_Engl.	quest_doo	2	2403,0	open door - gate	open door - gate	1	2-10 times	2	2	escape	0	1	0	0	0	0	0	g	2	19,0	NA	Warmblood	1	350	3	cooperativeness	1	single	2	unrestricted pasture	1	restricted	2	unrestrict roughage	
605	door_gate_Engl.	quest_doo	2	2414,0	open door - gate	open door - gate	1	2-10 times	2	5	play	0	0	0	0	0	0	1	s	3	21,0	NA	Arabian horse	3	4000	1	endurance	1	single	1	restricted pasture	1	restricted	2	unrestrict roughage	
606	door_gate_Engl.	quest_doo	2	2423,0	open door - gate	open door - gate, own door , stall door an	3	2-10 times	2	2	escape	0	1	0	0	0	0	0	m	1	13,0	NA	Draught horse	4	500	4	power	2	group	2	unrestricted pasture	2	unrestricted contact	2	unrestrict roughage	
607	door_gate_Engl.	quest_doo	2	2423,0	open door - gate	open door - gate, feed room door	3	2-10 times	2	1	feeding	1	0	0	0	0	0	0	m	1	13,0	NA	Draught horse	4	500	4	power	2	group	2	unrestricted pasture	2	unrestricted contact	2	unrestrict roughage	
608	door_gate_Engl.	quest_doo	2	2423,0	open door - gate	open door - gate, frees other horses	3	2-10 times	2	3	social	0	0	0	1	1	0	0	m	1	13,0	NA	Draught horse	4	500	4	power	2	group	2	unrestricted pasture	2	unrestricted contact	2	unrestrict roughage	
609	door_gate_Engl.	quest_doo	2	2423,1	open door - gate	open door - gate, own door , stall door an	2	2-10 times	2	2	escape	0	1	0	0	0	0	0	g	2		NA	Warmblood	1	350	3	cooperativeness	2	group	2	unrestricted pasture	2	unrestricted contact	2	unrestrict roughage	
610	door_gate_Engl.	quest_doo	2	2423,1	open door - gate	open door - gate, frees perferred horses	2	2-10 times	2	3	social	0	0	0	1	1	0	0	g	2		NA	Warmblood	1	350	3	cooperativeness	2	group	2	unrestricted pasture	2	unrestricted contact	2	unrestrict roughage	
611	door_gate_Engl.	quest_doo	2	2424,0	open door - gate	open door - gate, own door , stall door an	2	2-10 times	2	2	escape	0	1	0	0	0	0	0	g	2	8,0	NA	Warmblood	1	350	3	cooperativeness	1	single	2	unrestricted pasture	1	restricted	2	unrestrict roughage	
612	door_gate_Engl.	quest_doo	2	2424,0	open door - gate	open door - gate, feed room door	2	2-10 times	2	1	feeding	1	0	0	0	0	0	0	g	2	8,0	NA	Warmblood	1	350	3	cooperativeness	1	single	2	unrestricted pasture	1	restricted	2	unrestrict roughage	
613	door_gate_Engl.	quest_doo	2	2429,0	open door - gate	open door - gate	1	more than 20 times	4	2	escape	0	1	0	0	0	0	0	g	2	5,0	NA	Warmblood	1	350	3	cooperativeness	1	single	1	restricted pasture	1	restricted	2	unrestrict roughage	
614	door_gate_Engl.	quest_doo	2	2430,0	open door - gate	open door - gate	1	2-10 times	2	2	escape	0	1	0	0	0	0	0	g	2	4,0	NA	Pony	2	100	5	robustnes	2	group	1	restricted	2	unrestricted	1	restricted	
615	door_gate_Engl.	quest_doo	2	2438,0	open door - gate	open door - gate	1	2-10 times	2	2	escape	0	1	0	0	0	0	0	m	1	2,0	NA	Arabian horse	3	4000	1	endurance	2	group	2	unrestricted pasture	2	unrestricted contact	1	restricted roughage	

1	sorce	video / quest	video_quest_num	sID	name behaviour	behaviour	nr reported behaviours	how often was the behaviour shown	behaviour frequency	behaviour category num	behaviour_category	feeding behaviour	escape behaviour	social behaviour human	social behaviour horse	social behaviour	comfort behaviour	unknown play	sex	sex num	age	breed	breed-type	breed type num	length of domestication	purpose of domestication	purpose of domestication	single group stabling	single group stabling	restricted unrestricted pasture	restricted unrestricted pasture	restricted unrestricted contact with horses	restricted unrestricted contact with horses	restricted unrestric. roughage	restricted unrestricted roughage	links
616	door_gate_Engl.	quest_doo	2	2440,0	open door - gate	open door - gate, own door , stall door an	4	dayly	5	2	escape	0	1	0	0	0	0	0	g	2	6,0	NA	Warmblood	1	350	3	cooperativeness	2	group	2	unrestricted pasture	2	unrestricted contact	2	unrestrict roughage	
617	door_gate_Engl.	quest_doo	2	2440,0	open door - gate	open door - gate, feed room door	4	dayly	5	1	feeding	1	0	0	0	0	0	0	g	2	6,0	NA	Warmblood	1	350	3	cooperativeness	2	group	2	unrestricted pasture	2	unrestricted contact	2	unrestrict roughage	
618	door_gate_Engl.	quest_doo	2	2440,0	open door - gate	open door - gate, frees other horses	4	dayly	5	3	social	0	0	0	1	1	0	0	g	2	6,0	NA	Warmblood	1	350	3	cooperativeness	2	group	2	unrestricted pasture	2	unrestricted contact	2	unrestrict roughage	
619	door_gate_Engl.	quest_doo	2	2440,0	unty itself	unty itself	4	dayly	5	2	escape	0	1	0	0	0	0	0	g	2	6,0	NA	Warmblood	1	350	3	cooperativeness	2	group	2	unrestricted pasture	2	unrestricted contact	2	unrestrict roughage	
620	door_gate_Engl.	quest_doo	2	2458,0	open door - gate	open door - gate, own door , stall door an	4	more than 20 times	4	2	escape	0	1	0	0	0	0	0	g	2	30,0	NA	Warmblood	1	350	3	cooperativeness	2	group	2	unrestricted pasture	2	unrestricted contact	2	unrestrict roughage	
621	door_gate_Engl.	quest_doo	2	2458,0	open door - gate	open door - gate, frees other horses	4	more than 20 times	4	3	social	0	0	0	1	1	0	0	g	2	30,0	NA	Warmblood	1	350	3	cooperativeness	2	group	2	unrestricted pasture	2	unrestricted contact	2	unrestrict roughage	
622	door_gate_Engl.	quest_doo	2	2458,0	unty itself	unty itself	4	more than 20 times	4	2	escape	0	1	0	0	0	0	0	g	2	30,0	NA	Warmblood	1	350	3	cooperativeness	2	group	2	unrestricted pasture	2	unrestricted contact	2	unrestrict roughage	
623	door_gate_Engl.	quest_doo	2	2458,0	manipulate things	remove fence, caps	4	more than 20 times	4	5	play	0	0	0	0	0	0	1	g	2	30,0	NA	Warmblood	1	350	3	cooperativeness	2	group	2	unrestricted pasture	2	unrestricted contact	2	unrestrict roughage	
624	door_gate_Engl.	quest_doo	2	2478,0	open door - gate	open door - gate, own door , stall door an	4	dayly	5	2	escape	0	1	0	0	0	0	0	g	2	14,0	NA	Arabian horse	3	4000	1	endurance	1	single	1	restricted pasture	1	restricted	2	unrestrict roughage	
625	door_gate_Engl.	quest_doo	2	2478,0	open door - gate	open door - gate, feed room door	4	dayly	5	1	feeding	1	0	0	0	0	0	0	g	2	14,0	NA	Arabian horse	3	4000	1	endurance	1	single	1	restricted pasture	1	restricted	2	unrestrict roughage	
626	door_gate_Engl.	quest_doo	2	2478,0	open door - gate	open door - gate, frees other horses	4	dayly	5	3	social	0	0	0	1	1	0	0	g	2	14,0	NA	Arabian horse	3	4000	1	endurance	1	single	1	restricted pasture	1	restricted	2	unrestrict roughage	
627	door_gate_Engl.	quest_doo	2	2478,0	open door - gate	manipulate things, carreies objects around	4	dayly	5	5	play	0	0	0	0	0	0	1	g	2	14,0	NA	Arabian horse	3	4000	1	endurance	1	single	1	restricted pasture	1	restricted	2	unrestrict roughage	
628	door_gate_Engl.	quest_doo	2	2478,1	open door - gate	open door - gate, own door , stall door an	1	dayly	5	2	escape	0	1	0	0	0	0	0	m	1		NA	Arabian horse	3	4000	1	endurance	1	single	1	restricted pasture	1	restricted	2	unrestrict roughage	
629	door_gate_Engl.	quest_doo	2	2505,0	open door - gate	open door - gate, own door , stall door an	12	more than 20 times	4	2	escape	0	1	0	0	0	0	0	g	2	2,0	NA	Arabian horse	3	4000	1	endurance	2	group	2	unrestricted pasture	2	unrestricted contact	1	restricted roughage	
630	door_gate_Engl.	quest_doo	2	2505,0	open door - gate	open door - gate, feed room door	12	more than 20 times	4	1	feeding	1	0	0	0	0	0	0	g	2	2,0	NA	Arabian horse	3	4000	1	endurance	2	group	2	unrestricted pasture	2	unrestricted contact	1	restricted roughage	
631	door_gate_Engl.	quest_doo	2	2505,0	open door - gate	open door - gate, frees other horses	12	more than 20 times	4	3	social	0	0	0	1	1	0	0	g	2	2,0	NA	Arabian horse	3	4000	1	endurance	2	group	2	unrestricted pasture	2	unrestricted contact	1	restricted roughage	
632	door_gate_Engl.	quest_doo	2	2505,0	open door - gate	open door - gate, opens doors but does n	12	more than 20 times	4	5	play	0	0	0	0	0	0	1	g	2	2,0	NA	Arabian horse	3	4000	1	endurance	2	group	2	unrestricted pasture	2	unrestricted contact	1	restricted roughage	
633	door_gate_Engl.	quest_doo	2	2505,0	manipulate things	untied halter knots	12	more than 20 times	4	2	escape	0	1	0	0	0	0	0	g	2	2,0	NA	Arabian horse	3	4000	1	endurance	2	group	2	unrestricted pasture	2	unrestricted contact	1	restricted roughage	
634	door_gate_Engl.	quest_doo	2	2505,0	manipulates horses	picks up brush and tries to brush other hor	12	more than 20 times	4	3	social	0	0	0	1	1	0	0	g	2	2,0	NA	Arabian horse	3	4000	1	endurance	2	group	2	unrestricted pasture	2	unrestricted contact	1	restricted roughage	
635	door_gate_Engl.	quest_doo	2	2505,0	manipulate things	removes hoses that are filling water tanks	12	more than 20 times	4	5	play	0	0	0	0	0	0	1	g	2	2,0	NA	Arabian horse	3	4000	1	endurance	2	group	2	unrestricted pasture	2	unrestricted contact	1	restricted roughage	
636	door_gate_Engl.	quest_doo	2	2505,0	manipulate things	picks up jump poles and staggers around c	12	more than 20 times	4	5	play	0	0	0	0	0	0	1	g	2	2,0	NA	Arabian horse	3	4000	1	endurance	2	group	2	unrestricted pasture	2	unrestricted contact	1	restricted roughage	
637	door_gate_Engl.	quest_doo	2	2505,0	manipulate things	Escapes plastic covered t post electric fen	12	more than 20 times	4	2	escape	0	1	0	0	0	0	0	g	2	2,0	NA	Arabian horse	3	4000	1	endurance	2	group	2	unrestricted pasture	2	unrestricted contact	1	restricted roughage	
638	door_gate_Engl.	quest_doo	2	2505,0	manipulate things	picked up feed bins and bailed water out	12	more than 20 times	4	5	play	0	0	0	0	0	0	1	g	2	2,0	NA	Arabian horse	3	4000	1	endurance	2	group	2	unrestricted pasture	2	unrestricted contact	1	restricted roughage	
639	door_gate_Engl.	quest_doo	2	2505,0	manipulate persons	picked up a stick and took a swing at pers	12	more than 20 times	4	3	social	0	0	1	0	1	0	0	g	2	2,0	NA	Arabian horse	3	4000	1	endurance	2	group	2	unrestricted pasture	2	unrestricted contact	1	restricted roughage	
640	door_gate_Engl.	quest_doo	2	2505,0	manipulate things	Removes his jackets with all fasteners still	12	more than 20 times	4	4	comfort	0	0	0	0	0	1	0	g	2	2,0	NA	Arabian horse	3	4000	1	endurance	2	group	2	unrestricted pasture	2	unrestricted contact	1	restricted roughage	
641	door_gate_Engl.	video	3	2507,0	open door - gate	open door - gate, own door , stall door an	2	NA		2	escape	0	1	0	0	0	0	0	NA			NA	Donkey	7	6000	4	power	2	group	1	restricted	2	unrestricted			https://www.youtube.com/watch?v=AN6I0Ehu7O0
642	door_gate_Engl.	video	3	2507,0	open door - gate	open door - gate, frees other donkey	2	NA		3	social	0	0	0	1	1	0	0	NA			NA	Donkey	7	6000	4	power	2	group	1	restricted	2	unrestricted			https://www.youtube.com/watch?v=AN6I0Ehu7O0
643	door_gate_Engl.	video	3	2508,0	open door - gate	open door - gate	1	NA		2	escape	0	1	0	0	0	0	0	NA			NA	Donkey	7	6000	4	power	1	single	2	unrestricted pasture	1	restricted			https://www.youtube.com/watch?v=xEfotAYK_TE
644	door_gate_Engl.	video	3	2511,0	open door - gate	open door - gate	1	NA		2	escape	0	1	0	0	0	0	0	g	2		NA	Mule	7	3000	4	power	1	single	1	restricted pasture	1	restricted	1	restricted roughage	https://www.youtube.com/watch?v=QiEYv4fA9vE
645	door_gate_Frens	video	3	2459,0	open door - gate	open door - gate	1	dayly	5	2	escape	0	1	0	0	0	0	0	g	2	7,0	NA	Warmblood	1	350	3	cooperativeness	1	single	1	restricted pasture	1	restricted	1	restricted roughage	
646	door_gate_Frens	video	3	2471,0	open door - gate	open door - gate, own door , stall door an	2	dayly	5	2	escape	0	1	0	0	0	0	0	s	3	6,0	NA	NA					1	single	1	restricted pasture	1	restricted	1	restricted roughage	
647	door_gate_Frens	video	3	2471,0	open door - gate	open door - gate, frees other horses	2	dayly	5	3	social	0	0	0	1	1	0	0	s	3	6,0	NA	NA					1	single	1	restricted pasture	1	restricted	1	restricted roughage	
648	door_gate_Frens	quest_doo	2	2469,0	open door - gate	open door - gate, breakes into box for fee	2	2-10 times	2	1	feeding	1	0	0	0	0	0	0	g	2	17,0	NA	Pony	2	100	5	robustness	1	single	1	restricted pasture	1	restricted	1	restricted roughage	
649	door_gate_Frens	quest_doo	2	2469,0	open door - gate	open door - gate, frees other horses	2	2-10 times	2	3	social	0	0	0	1	1	0	0	g	2	17,0	NA	Pony	2	100	5	robustness	1	single	1	restricted pasture	1	restricted	1	restricted roughage	
650	door_gate_Frens	quest_doo	2	2467,0	open door - gate	open door - gate, own door , stall door an	4	more than 20 times	4	2	escape	0	1	0	0	0	0	0	g	2	2,0	NA	Warmblood	1	350	3	cooperativeness	1	single	1	restricted pasture	1	restricted	1	restricted roughage	

1	sorce	video / quest	video_quest_num	sID	name behaviour	behaviour	nr reported behaviours	how often was the behaviour shown	behaviour frequency	behaviour category num	behaviour _category	feeding behaviour	escape behaviour	social behaviour human	social behaviour horse	social behaviour	comfort behaviour	unknown play	sex	sex num	age	breed	breed-type	breed type num	length of domestication	purpose of domestication	purpose of domestication	single group stabling	single group stabling	restricted unrestricted pasture	restricted unrestricted pasture	restricted unrestricted contact with horses	restricted unrestricted contact with horses	restricted unrestric. roughage	restricted unrestricted roughage	links
651	door_gate_Frens	quest_doo	2	2467,0	open door - gate	open door - gate, feed room door	4	more than 20 times	4	1	feeding	1	0	0	0	0	0	0	g	2	2,0	NA	Warmblood	1	350	3	cooperativeness	1	single	1	restricted pasture	1	restricted	1	restricted roughage	
652	door_gate_Frens	quest_doo	2	2467,0	open door - gate	open door - gate, frees other horses	4	more than 20 times	4	3	social	0	0	0	1	1	0	0	g	2	2,0	NA	Warmblood	1	350	3	cooperativeness	1	single	1	restricted pasture	1	restricted	1	restricted roughage	
653	door_gate_Frens	quest_doo	2	2467,0	open door - gate	open door - gate, opens door but does no	4	more than 20 times	4	5	play	0	0	0	0	0	0	1	g	2	2,0	NA	Warmblood	1	350	3	cooperativeness	1	single	1	restricted pasture	1	restricted	1	restricted roughage	
654	door_gate_Frens	quest_doo	2	2466,1	open door - gate	open door - gate	1	11-20 times	3	2	escape	0	1	0	0	0	0	0	m	1	2,0	NA	Arabian horse	3	4000	1	endurance	1	single	1	restricted pasture	1	restricted	1	restricted roughage	
655	door_gate_Frens	quest_doo	2	2465,0	open door - gate	open door - gate, own door , stall door an	2	2-10 times	2	2	escape	0	1	0	0	0	0	0	g	2	14,0	NA	Warmblood	1	350	3	cooperativeness	1	single	1	restricted pasture	1	restricted	2	unrestrict roughage	
656	door_gate_Frens	quest_doo	2	2465,0	open door - gate	open door - gate, feed room door	2	2-10 times	2	1	feeding	1	0	0	0	0	0	0	g	2	14,0	NA	Warmblood	1	350	3	cooperativeness	1	single	1	restricted pasture	1	restricted	2	unrestrict roughage	
657	door_gate_Frens	quest_doo	2	2463,0	open door - gate	open door - gate, own door , stall door an	2	11-20 times	3	2	escape	0	1	0	0	0	0	0	g	2	28,0	NA	NA					2	group	2	unrestricted pasture	2	unrestricted contact	1	restricted roughage	
658	door_gate_Frens	quest_doo	2	2463,0	open door - gate	open door - gate, feed room door	2	11-20 times	3	1	feeding	1	0	0	0	0	0	0	g	2	28,0	NA	NA					2	group	2	unrestricted pasture	2	unrestricted contact	1	restricted roughage	
659	door_gate_Frens	quest_doo	2	2462,0	open door - gate	open door - gate	1	2-10 times	2	2	escape	0	1	0	0	0	0	0	g	2	13,0	NA	Warmblood	1	350	3	cooperativeness	1	single	1	restricted pasture	1	restricted	2	unrestrict roughage	
660	door_gate_Frens	quest_doo	2	2461,0	open door - gate	open door - gate, own door , stall door an	2	dayly	5	2	escape	0	1	0	0	0	0	0	g	2	26,0	NA	Warmblood	1	350	3	cooperativeness	1	single	1	restricted pasture	1	restricted	1	restricted roughage	
661	door_gate_Frens	quest_doo	2	2461,0	open door - gate	open door - gate, feed room door	2	dayly	5	1	feeding	1	0	0	0	0	0	0	g	2	26,0	NA	Warmblood	1	350	3	cooperativeness	1	single	1	restricted pasture	1	restricted	1	restricted roughage	
662	door_gate_Frens	quest_doo	2	2460,0	open door - gate	open door - gate, own door , stall door an	2	more than 20 times	4	2	escape	0	1	0	0	0	0	0	g	2	8,0	NA	NA					1	single	1	restricted pasture	1	restricted	1	restricted roughage	
663	door_gate_Frens	quest_doo	2	2460,0	open door - gate	open door - gate, feed room door	2	more than 20 times	4	1	feeding	1	0	0	0	0	0	0	g	2	8,0	NA	NA					1	single	1	restricted pasture	1	restricted	1	restricted roughage	
664	door_gate_Frens	quest_doo	2	2472,0	open door - gate	open door - gate	1	dayly	5	5	play	0	0	0	0	0	0	1	m	1	8,0	NA	Pony	2	100	5	robustness	1	single	1	restricted pasture	1	restricted	2	unrestrict roughage	
665	door_gate_Frens	quest_doo	2	2473,0	open door - gate	open door - gate	1	dayly	5	2	escape	0	1	0	0	0	0	0	m	1		NA	Arabian horse	3	4000	1	endurance	1	single	2	unrestricted pasture	1	restricted	1	restricted roughage	
666	door_gate_Frens	quest_doo	2	2476,0	open door - gate	open door - gate, own door , stall door an	2	NA		2	escape	0	1	0	0	0	0	0	g	2	18,0	NA	Arabian horse	3	4000	1	endurance	2	group	2	unrestricted pasture	2	unrestricted contact	2	unrestrict roughage	
667	door_gate_Frens	quest_doo	2	2476,0	open door - gate	open door - gate, gate to pasture	2	NA		1	feeding	1	0	0	0	0	0	0	g	2	18,0	NA	Arabian horse	3	4000	1	endurance	2	group	2	unrestricted pasture	2	unrestricted contact	2	unrestrict roughage	
668	door_gate_Frens	quest_doo	2	2477,0	open door - gate	open door - gate, own door , stall door an	2	NA		2	escape	0	1	0	0	0	0	0	g	2	7,0	NA	Warmblood	1	350	3	cooperativeness	1	single	1	restricted pasture	1	restricted	1	restricted roughage	
669	door_gate_Frens	quest_doo	2	2477,0	open door - gate	open door - gate, feed room door	2	NA		1	feeding	1	0	0	0	0	0	0	g	2	7,0	NA	Warmblood	1	350	3	cooperativeness	1	single	1	restricted pasture	1	restricted	1	restricted roughage	
670	door_gate_Frens	quest_doo	2	2479,0	open door - gate	open door - gate	1	dayly	5	2	escape	0	1	0	0	0	0	0	g	2	13,0	NA	Warmblood	1	350	3	cooperativeness	1	single	1	restricted pasture	1	restricted	1	restricted roughage	
671	door_gate_Frens	quest_doo	2	2481,0	open door - gate	open door - gate, own door , stall door an	2	11-20 times	3	2	escape	0	1	0	0	0	0	0	g	2	11,0	NA	Warmblood	1	350	3	cooperativeness	1	single	1	restricted pasture	1	restricted	1	restricted roughage	
672	door_gate_Frens	quest_doo	2	2481,0	open door - gate	open door - gate, frees other horses	2	11-20 times	3	3	social	0	0	0	1	1	0	0	g	2	11,0	NA	Warmblood	1	350	3	cooperativeness	1	single	1	restricted pasture	1	restricted	1	restricted roughage	
673	door_gate_Frens	quest_doo	2	2483,0	open door - gate	open door - gate	1	dayly	5	2	escape	0	1	0	0	0	0	0	m	1	5,0	NA	NA					2	group	1	restricted	2	unrestricted	1	restricted	
674	door_gate_Frens	quest_doo	2	2485,0	open door - gate	open door - gate, own door , stall door an	2	2-10 times	2	2	escape	0	1	0	0	0	0	0	g	2	12,0	NA	Arabian horse	3	4000	1	endurance	1	single	1	restricted pasture	1	restricted	1	restricted roughage	
675	door_gate_Frens	quest_doo	2	2485,0	open door - gate	open door - gate, gate to pasture	2	2-10 times	2	1	feeding	1	0	0	0	0	0	0	g	2	12,0	NA	Arabian horse	3	4000	1	endurance	1	single	1	restricted pasture	1	restricted	1	restricted roughage	
676	door_gate_Frens	quest_doo	2	2486,0	open door - gate	open door - gate, own door , stall door an	3	dayly	5	2	escape	0	1	0	0	0	0	0	g	2	4,0	NA	Warmblood	1	350	3	cooperativeness	1	single	1	restricted pasture	1	restricted	2	unrestrict roughage	
677	door_gate_Frens	quest_doo	2	2486,0	open door - gate	open door - gate, feed room door	3	dayly	5	1	feeding	1	0	0	0	0	0	0	g	2	4,0	NA	Warmblood	1	350	3	cooperativeness	1	single	1	restricted pasture	1	restricted	2	unrestrict roughage	
678	door_gate_Frens	quest_doo	2	2486,0	open door - gate	open door - gate, frees other horses	3	dayly	5	3	social	0	0	0	1	1	0	0	g	2	4,0	NA	Warmblood	1	350	3	cooperativeness	1	single	1	restricted pasture	1	restricted	2	unrestrict roughage	
679	door_gate_Frens	quest_doo	2	2486,1	open door - gate	open door - gate	1	dayly	5	2	escape	0	1	0	0	0	0	0	m	1		NA	Warmblood	1	350	3	cooperativeness	1	single	1	restricted pasture	1	restricted	2	unrestrict roughage	
680	door_gate_Frens	quest_doo	2	2487,0	open door - gate	open door - gate, own door , stall door an	2	2-10 times	2	2	escape	0	1	0	0	0	0	0	g	2	10,0	NA	Warmblood	1	350	3	cooperativeness	1	single	1	restricted pasture	1	restricted	1	restricted roughage	
681	door_gate_Frens	quest_doo	2	2487,0	open door - gate	open door - gate, feed room door	2	2-10 times	2	1	feeding	1	0	0	0	0	0	0	g	2	10,0	NA	Warmblood	1	350	3	cooperativeness	1	single	1	restricted pasture	1	restricted	1	restricted roughage	
682	door_gate_Frens	quest_doo	2	2488,0	open door - gate	open door - gate, own door , stall door an	2	dayly	5	2	escape	0	1	0	0	0	0	0	m	1	20,0	NA	Warmblood	1	350	3	cooperativeness	1	single	1	restricted	1	restricted	1	restricted	
683	door_gate_Frens	quest_doo	2	2488,0	open door - gate	open door - gate, frees other horses	2	dayly	5	3	social	0	0	0	1	1	0	0	m	1	20,0	NA	Warmblood	1	350	3	cooperativeness	1	single	1	restricted	1	restricted	1	restricted	
684	door_gate_Frens	quest_doo	2	2489,0	open door - gate	open door - gate, feed room door	3	more than 20 times	4	1	feeding	1	0	0	0	0	0	0	m	1	16,0	NA	Warmblood	1	350	3	cooperativeness	2	group	2	unrestricted	2	unrestricted	2	unrestrict	
685	door_gate_Frens	quest_doo	2	2489,0	open door - gate	open door - gate, opens door but does ot	3	more than 20 times	4	5	play	0	0	0	0	0	0	1	m	1	16,0	NA	Warmblood	1	350	3	cooperativeness	2	group	2	unrestricted	2	unrestricted	2	unrestrict	

1	sorce	video / quest	video_quest_num	sID	name behaviour	behaviour	nr reported behaviours	how often was the behaviour shown	behaviour frequency	behaviour category num	behaviour_category	feeding behaviour	escape behaviour	social behaviour human	social behaviour horse	social behaviour	comfort behaviour	unknown play	sex
686	door_gate_Frens	quest_doo	2	2489,0	manipulate things	goes through electric fence	3	more than 20 times	4	2	escape	0	1	0	0	0	0	0	m
687	door_gate_Frens	quest_doo	2	2494,0	open door - gate	open door - gate, own door , stall door an	2	2-10 times	2	2	escape	0	1	0	0	0	0	0	m
688	door_gate_Frens	quest_doo	2	2494,0	open door - gate	open door - gate, feed room door	2	2-10 times	2	1	feeding	1	0	0	0	0	0	0	m
689	door_gate_Frens	quest_doo	2	2495,0	open door - gate	open door - gate, own door , stall door an	2	2-10 times	2	2	escape	0	1	0	0	0	0	0	g
690	door_gate_Frens	quest_doo	2	2495,0	open door - gate	open door - gate, feed room door	2	2-10 times	2	1	feeding	1	0	0	0	0	0	0	g
691	door_gate_Frens	quest_doo	2	2496,0	open door - gate	open door - gate, own door , stall door an	3	dayly	5	2	escape	0	1	0	0	0	0	0	g
692	door_gate_Frens	quest_doo	2	2496,0	open door - gate	open door - gate, feed room door	3	dayly	5	1	feeding	1	0	0	0	0	0	0	g
693	door_gate_Frens	quest_doo	2	2496,0	open door - gate	open door - gate, opens doors but does n	3	dayly	5	5	play	0	0	0	0	0	0	1	g
694	door_gate_Frens	quest_doo	2	2497,0	open door - gate	open door - gate, feed room door	2	more than 20 times	4	1	feeding	1	0	0	0	0	0	0	g
695	door_gate_Frens	quest_doo	2	2497,0	open door - gate	open door - gate, opens door but does nc	2	more than 20 times	4	5	play	0	0	0	0	0	0	1	g
696	door_gate_Frens	quest_doo	2	2501,0	open door - gate	open door - gate, own door , stall door an	2	more than 20 times	4	2	escape	0	1	0	0	0	0	0	m
697	door_gate_Frens	quest_doo	2	2501,0	open door - gate	open door - gate, feed room door	2	more than 20 times	4	1	feeding	1	0	0	0	0	0	0	m
698	door_gate_Frens	quest_doo	2	2503,0	open door - gate	open door - gate, own door , stall door an	2	more than 20 times	4	2	escape	0	1	0	0	0	0	0	g
699	door_gate_Frens	quest_doo	2	2503,0	open door - gate	open door - gate, frees other horses	2	more than 20 times	4	3	social	0	0	0	1	1	0	0	g
700	door_gate_Germ	video	3	2512,0	open door - gate	open door - gate, own door , stall door an	2	NA		2	escape	0	1	0	0	0	0	0	NA
701	door_gate_Germ	video	3	2512,0	open door - gate	open door - gate, frees other horses	2	NA		3	social	0	0	0	1	1	0	0	NA
702	door_gate_Germ	video	3	2513,0	open door - gate	open door - gate	1	NA		2	escape	0	1	0	0	0	0	0	NA
703	door_gate_Germ	video	3	2514,0	open door - gate	open door - gate, own door , stall door an	2	NA		2	escape	0	1	0	0	0	0	0	g
704	door_gate_Germ	video	3	2514,0	open door - gate	open door - gate, frees other horses	2	NA		3	social	0	0	0	1	1	0	0	g
705	door_gate_Germ	video	3	2515,0	open door - gate	open door - gate	1	NA		2	escape	0	1	0	0	0	0	0	g
706	door_gate_Germ	video	3	2516,0	open door - gate	open door - gate	1	NA		2	escape	0	1	0	0	0	0	0	g
707	door_gate_Germ	video	3	2517,0	open door - gate	open door - gate	1	NA		2	escape	0	1	0	0	0	0	0	NA
708	door_gate_Germ	video	3	2518,0	open door - gate	open door - gate, feed room door	2	NA		2	escape	0	1	0	0	0	0	0	NA
709	door_gate_Germ	video	3	2518,0	open door - gate	open door - gate, frees other horses	2	NA		3	social	0	0	0	1	1	0	0	NA
710	door_gate_Germ	video	3	2518,0	open door - gate	open door - gate, opens doors but does n	1	NA		5	play	0	0	0	0	0	0	1	NA
711	door_gate_Germ	video	3	2538,0	open door - gate	open door - gate	1	NA		2	escape	0	1	0	0	0	0	0	NA
712	door_gate_Germ	video	3	2539,0	open door - gate	open door - gate	1	NA		2	escape	0	1	0	0	0	0	0	m
713	door_gate_Germ	video	3	2519,0	open door - gate	open door - gate, frees other horses	2	NA		3	social	0	0	0	1	1	0	0	NA
714	door_gate_Germ	video	3	2519,0	open door - gate	open door - gate, opens doors but does n	2	NA		5	play	0	0	0	0	0	0	1	NA
715	door_gate_Germ	video	3	2520,0	open door - gate	open door - gate	1	NA		2	escape	0	1	0	0	0	0	0	NA
716	door_gate_Germ	video	3	2521,0	open door - gate	open door - gate	1	NA		2	escape	0	1	0	0	0	0	0	NA
717	door_gate_Germ	video	3	2522,0	open door - gate	open door - gate	1	NA		2	escape	0	1	0	0	0	0	0	NA
718	door_gate_Germ	video	3	2523,0	open door - gate	open door - gate	1	NA		2	escape	0	1	0	0	0	0	0	NA
719	door_gate_Germ	video	3	2524,0	open door - gate	open door - gate	1	NA		2	escape	0	1	0	0	0	0	0	NA
720	door_gate_Germ	video	3	2525,0	open door - gate	open door - gate	1	NA		2	escape	0	1	0	0	0	0	0	NA
721	door_gate_Germ	video	3	2526,0	open door - gate	open door - gate	1	NA		2	escape	0	1	0	0	0	0	0	NA
722	door_gate_Germ	video	3	2527,0	open door - gate	open door - gate	1	NA		2	escape	0	1	0	0	0	0	0	g
723	door_gate_Germ	video	3	2528,0	open door - gate	open door - gate	1	NA		2	escape	0	1	0	0	0	0	0	NA
724	door_gate_Germ	video	3	2529,0	open door - gate	open door - gate	1	NA		2	escape	0	1	0	0	0	0	0	NA
725	door_gate_Germ	video	3	2530,0	open door - gate	open door - gate	1	NA		2	escape	0	1	0	0	0	0	0	g

1	sex num	age	breed	breed-type	breed type num	length of domestication	purpose of domestication	purpose of domestication	single group stabling	single group stabling	restricted unrestricted pasture	restricted unrestricted pasture	restricted unrestricted contact with horses	restricted unrestricted contact with horses	restricted unrestric. roughage	restricted unrestricted roughage	links
686	1	16,0	NA	Warmblood	1	350	3	cooperativeness	2	group	2	unrestricted	2	unrestricted	2	unrestrict	
687	1	8,0	NA	Pony	2	100	5	robustnes	2	group	2	unrestricted	2	unrestricted	2	unrestrict	
688	1	8,0	NA	Pony	2	100	5	robustnes	2	group	2	unrestricted	2	unrestricted	2	unrestrict	
689	2	9,0	NA	Warmblood	1	350	3	cooperativeness	1	single	1	restricted	1	restricted	1	restricted	
690	2	9,0	NA	Warmblood	1	350	3	cooperativeness	1	single	1	restricted	1	restricted	1	restricted	
691	2	12,0	NA	Warmblood	1	350	3	cooperativeness	1	single	1	restricted	1	restricted	1	restricted	
692	2	12,0	NA	Warmblood	1	350	3	cooperativeness	1	single	1	restricted	1	restricted	1	restricted	
693	2	12,0	NA	Warmblood	1	350	3	cooperativeness	1	single	1	restricted	1	restricted	1	restricted	
694	2	6,0	NA	Arabian horse	3	4000	1	endurance	2	group	2	unrestricted	2	unrestricted	2	unrestrict	
695	2	6,0	NA	Arabian horse	3	4000	1	endurance	2	group	2	unrestricted	2	unrestricted	2	unrestrict	
696	1	14,0	NA	Pony	2	100	5	robustnes	2	group	2	unrestricted	2	unrestricted	2	unrestrict	
697	1	14,0	NA	Pony	2	100	5	robustnes	2	group	2	unrestricted	2	unrestricted	2	unrestrict	
698	2	8,0	NA	Warmblood	1	350	3	cooperativeness	2	group	1	restricted	2	unrestricted	1	restricted	
699	2	8,0	NA	Warmblood	1	350	3	cooperativeness	2	group	1	restricted	2	unrestricted	1	restricted	
700			NA	Warmblood	1	350	3	cooperativeness	1	single	1	restricted	1	restricted	1	restricted	https://www.youtube.com/watch?v=IXebjTbLT4M
701			NA	Warmblood	1	350	3	cooperativeness	1	single	1	restricted	1	restricted	1	restricted	https://www.youtube.com/watch?v=IXebjTbLT4M
702			NA	Warmblood	1	350	3	cooperativeness	1	single	1	restricted	1	restricted			https://www.youtube.com/watch?v=-R_e-zd5zr0
703	2		NA	Warmblood	1	350	3	cooperativeness	1	single	1	restricted	1	restricted	1	restricted	https://www.youtube.com/watch?v=krnNwRdlkJo
704	2		NA	Warmblood	1	350	3	cooperativeness	1	single	1	restricted	1	restricted	1	restricted	https://www.youtube.com/watch?v=krnNwRdlkJo
705	2	6,0	NA	Arabian horse	3	4000	1	endurance	1	single	1	restricted	1	restricted	1	restricted	https://www.youtube.com/watch?v=bo60IBvt5jw
706	2		NA	Warmblood	1	350	3	cooperativeness	1	single	1	restricted	1	restricted	1	restricted	https://www.youtube.com/watch?v=qBjlyI96E3g
707			NA	Pony	2	100	5	robustnes									https://www.youtube.com/watch?v=rZLbI5xcV9E
708			NA	Warmblood	1	350	3	cooperativeness	1	single	1	restricted	1	restricted	1	restricted	https://www.youtube.com/watch?v=W3KnTX0RI5s
709			NA	Warmblood	1	350	3	cooperativeness	1	single	1	restricted	1	restricted	1	restricted	https://www.youtube.com/watch?v=W3KnTX0RI5s
710			NA	Warmblood	1	350	3	cooperativeness	1	single	1	restricted	1	restricted	1	restricted	https://www.youtube.com/watch?v=W3KnTX0RI5s
711			NA	Warmblood	1	350	3	cooperativeness	1	single	1	restricted	1	restricted	1	restricted	https://www.youtube.com/watch?v=8IE1HHGuJ-E
712	1		NA	Warmblood	1	350	3	cooperativeness	1	single	1	restricted	1	restricted			https://www.youtube.com/watch?v=QnVISUkqMAo
713			NA	Warmblood	1	350	3	cooperativeness	1	single	1	restricted	1	restricted	1	restricted	https://www.youtube.com/watch?v=0qUFsYN8-Ec
714			NA	Warmblood	1	350	3	cooperativeness	1	single	1	restricted	1	restricted	1	restricted	https://www.youtube.com/watch?v=0qUFsYN8-Ec
715			NA	Warmblood	1	350	3	cooperativeness	1	single	1	restricted	1	restricted			https://www.youtube.com/watch?v=ul-u7-_wA94
716			NA	Warmblood	1	350	3	cooperativeness	1	single	1	restricted	1	restricted	1	restricted	https://www.youtube.com/watch?v=A9V-ePJZfWo
717			NA	Pony	2	100	5	robustnes	1	single	1	restricted	1	restricted	1	restricted	https://www.youtube.com/watch?v=m3cIQNIGBQ4
718		0,5	NA	NA													https://www.youtube.com/watch?v=YUc9gmUVQ6U
719			NA	Warmblood	1	350	3	cooperativeness	1	single	1	restricted	1	restricted	1	restricted	https://www.youtube.com/watch?v=Fg-wViXmc1Y
720			NA	Warmblood	1	350	3	cooperativeness	2	group			2	unrestricted			https://www.youtube.com/watch?v=VqYcJrye6VU
721			NA	Warmblood	1	350	3	cooperativeness	1	single	1	restricted	1	restricted			https://www.youtube.com/watch?v=g_DzG4vkmHc
722	2		NA	Warmblood	1	350	3	cooperativeness	1	single	1	restricted	1	restricted			https://www.youtube.com/watch?v=zwsm07q3y7M
723			NA	Pony	2	100	5	robustnes									https://www.youtube.com/watch?v=EKAROqJvYDs
724			NA	Pony	2	100	5	robustnes									https://www.youtube.com/watch?v=lu8LwlvZqvk
725	2		NA	Warmblood	1	350	3	cooperativeness									https://www.youtube.com/watch?v=rDU4BIJFj9c

1	sorce	video / quest	video_quest_num	sID	name behaviour	behaviour	nr reported behaviours	how often was the behaviour shown	behaviour frequency	behaviour category num	behaviour _category	feeding behaviour	escape behaviour	social behaviour human	social behaviour horse	social behaviour	comfort behaviour	unknown play
726	door_gate_Germ	video	3	2531,0	open door - gate	open door - gate	1	NA		2	escape	0	1	0	0	0	0	0
727	door_gate_Germ	video	3	2532,0	open door - gate	open door - gate	1	NA		2	escape	0	1	0	0	0	0	0
728	door_gate_Germ	video	3	2533,0	open door - gate	open door - gate	1	NA		2	escape	0	1	0	0	0	0	0
729	door_gate_Germ	video	3	2534,0	open door - gate	open door - gate	1	NA		2	escape	0	1	0	0	0	0	0
730	door_gate_Germ	video	3	2535,0	open door - gate	open door - gate	1	NA		2	escape	0	1	0	0	0	0	0
731	door_gate_Germ	video	3	2536,0	open door - gate	open door - gate	1	NA		2	escape	0	1	0	0	0	0	0
732	door_gate_Germ	video	3	2537,0	open door - gate	open door - gate	1	NA		2	escape	0	1	0	0	0	0	0
733	door_gate_Germ	video	3	2540,0	open door - gate	open door - gate	1	NA		2	escape	0	1	0	0	0	0	0
734	gen.Germ.	quest	1	2552,0	tool use	throws feed bowl in front of person when he wants to be fed	4	more than 20 times	4	3	social	0	0	1	0	1	0	0
735	gen.Germ.	quest	1	2552,0	borederline tool use	steps on bowl to collect water	4	more than 20 times	4	1	feeding	1	0	0	0	0	0	0
736	gen.Germ.	quest	1	2552,0	manipulate things	extinguish fire by stepping on smoldering	4	more than 20 times	4	5	play	0	0	0	0	0	0	1
737	gen.Germ.	quest	1	2552,0	manipulate things	climbs on bucket	4	more than 20 times	4	5	play	0	0	0	0	0	0	1
738	gen.Engl.	quest	1	2543,0	manipulate things	turning ligt on and off	1	2 - 10 times	2	5	play	0	0	0	0	0	0	1
739	gen.Engl.	quest	1	2550,0	open door	opens gate to stay closer to neighbouring horse	1	more than 20 times	4	2	escape	0	1	0	0	0	0	0
740	door_gate_Ger	quest_do	2	2542,0	open door	open own door	2	2 - 10 times	2	2	escape	0	1	0	0	0	0	0
741	door_gate_Ger	quest_do	2	2542,0	open door	open door of feed room	2	2 - 10 times	2	1	feeding	1	0	0	0	0	0	0
742	door_gate_Germ.	quest_door	2	2545,0	open door	opens gate for access to ad libitum feeding area	1	more than 20 times	4	1	feeding	1	0	0	0	0	0	0
743	door_gate_Germ.	quest_door	2	2545,1	open door	opens carabines by twisting chain attached to the carabiner, does not run	1	more than 20 times	4	5	play	0	0	0	0	0	0	1
744	door_gate_Germ.	quest_door	2	2551,0	open door	open door	1	more than 20 times	4	2	escape	0	1	0	0	0	0	0
745	gen Germ	video	3	2556,0	roll snow balls	feeding behaviour	1	NA		1	feeding	1	0	0	0	0	0	0
746	gen Germ	video	3	2555,0	lies underneath fence and eats grass	feeding behaviour	1	NA		1	feeding	1	0	0	0	0	0	0
747	gen.Germ.	quest	1	2554,0	open sliding door	Escape behaviour	1	NA		2	escape	0	1	0	0	0	0	0

1	sex	sex num	age	breed	breed-type	breed type num	length of domestication	purpose of domestication	purpose of domestication	single group stabling	single group stabling	restricted unrestricted pasture	restricted unrestricted pasture	restricted unrestricted contact with horses	restricted unrestricted contact with horses	restricted unrestric. roughage	restricted unrestricted roughage	links
726	NA			NA	Warmblood	1	350	3	cooperativeness	1	single	1	restricted	1	restricted			https://www.youtube.com/watch?v=AUGKbhZGTJw
727	m	1		NA	Pony	2	100	5	robustnes									https://www.youtube.com/watch?v=8JlrxgDxyZ0
728	NA			NA	Pony	2	100	5	robustnes									https://www.youtube.com/watch?v=G1rqmvbJcrs
729	g	2		NA	Warmblood	1	350	3	cooperativeness	1	single	1	restricted	1	restricted	1	restricted	https://www.youtube.com/watch?v=IlwN-PxdTrw
730	NA			NA	Pony	2	100	5	robustnes									https://www.youtube.com/watch?v=3iJMVjydtcM
731	NA			NA	Warmblood	1	350	3	cooperativeness	1	single	1	restricted	1	restricted	1	restricted	https://www.youtube.com/watch?v=F-ZWQt7Htfw
732	NA			NA	Warmblood	1	350	3	cooperativeness									https://www.youtube.com/watch?v=rDMYQbe5Mwk
733	NA			NA	warmblood	1	350	3	cooperativeness									https://www.youtube.com/watch?v=-nndf7Mhc_o
734	g	2	10,0	unknown						1	single	2	unrestricted	1	restricted	2	unrestrict	
735	g	2	10,0	unknown						1	single	2	unrestricted	1	restricted	2	unrestrict	
736	g	2	10,0	unknown						1	single	2	unrestricted	1	restricted	2	unrestrict	
737	g	2	10,0	unknown						1	single	2	unrestricted	1	restricted	2	unrestrict	
738	g	2	23,0	Quater horse	Warmblood	1	350	3	cooperativeness	1	single	2	unrestricted	2	unrestricted	2	unrestrict	
739	g	2	5,0	Quater horse	Warmblood	1	350	3	cooperativeness	2	group	2	unrestricted	2	unrestricted	2	unrestrict	
740	g	2	12,0	Islandic horse	Pony	2	100	5	robustnes	2	group	2	unrestricted	2	unrestricted	1	restricted	
741	g	2	12,0	Islandic horse	Pony	2	100	5	robustnes	2	group	2	unrestricted	2	unrestricted	1	restricted	
742	s	3	23,0	Quater mix	Warmblood	1	350	3	cooperativeness	2	group	2	unrestricted	2	unrestricted	2	unrestrict	
743	g	2		Arabmix	Arabian horse	3	4000	1	endurance	2	group	2	unrestricted	2	unrestricted	2	unrestrict	
744	m	1	12,0	Haflinger	Warmblood	1	350	3	cooperativeness	2	group	2	unrestricted	2	unrestricted	2	unrestrict	
745				NA	Warmblood	1	350	3	cooperativeness									video at: https://m.facebook.com/story.php?story_fbid=10211088627913819&id=1299768134
746				NA	Warmblood	1	350	3	cooperativeness									https://www.youtube.com/watch?v=sJeLi7OqHJQ
747	m	1	19,0	Hessian Warmblood	Warmblood	1	350	3	cooperativeness	1	single	1	restricted	1	restricted	2	unrestrict	
